# Global, regional and national burden of gastroesophageal reflux disease, 1990–2019: update from the GBD 2019 study

**DOI:** 10.1080/07853890.2022.2074535

**Published:** 2022-05-17

**Authors:** Decai Zhang, Shaojun Liu, Zhaoqi Li, Rui Wang

**Affiliations:** a Department of Gastroenterology, The Third Xiangya Hospital of Central South University, Changsha, China; b Hunan Key Laboratory of Nonresolving Inflammation and Cancer, Changsha, China

**Keywords:** Gastroesophageal reflux disease, global burden of disease, prevalence, epidemiology

## Abstract

**Background:**

Because trends in the epidemiology and burden of gastroesophageal reflux disease (GERD) are changing, reinvestigating the geographical differences and trend changes is essential. Here we evaluated the latest epidemiologic patterns and trends for GERD, using data from Global Burden of Diseases, Injuries, and Risk Factors Study (GBD) 2019.

**Methods:**

Annual case numbers, age-standardized rates of prevalence, incidence, and years of life lived with disability (YLDs), and their estimated annual percentage changes (EAPCs) for GERD between 1990 and 2019 were derived from the GBD 2019 study. Association between GERD burden and socio-demographic index (SDI) was also investigated.

**Results:**

In 2019, there were 783.95 million cases of GERD globally. Between 1990 and 2019, the total number of prevalent cases, incident cases, and YLDs increased by 77.53%, 74.79%, and 77.19%, respectively. The global age-standardized incidence rate (ASIR) and age-standardized YLD rate (ASYR) increased during this period (EAPC = 0.06 and 0.05, respectively). Tropical Latin America and East Asia had the highest and lowest age-standardiZed prevalence rate (ASPR), ASIR, and ASYR in 2019, respectively. From 1990 to 2019, prevalent cases, incident cases, YLDs, and their corresponding age-standardized rates of GERD were higher in females than males in all years. Higher SDI was associated with lower ASPR, ASIR, and ASYR of GERD in 2019.

**Conclusions:**

GERD will continue to be a major public health burden due to increasing numbers of prevalent cases, incident cases, and YLDs. In order to tackle this troublesome disease, it is crucial to understand the changes in both global and regional trends in epidemiology and the burden for policymakers and other stakeholders.

**Key messages**
- This is the most updated estimate on GERD epidemiology globally, including 204 countries, some of which were not assessed before.
- The overall burden of GERD continued to worsen with the prevalent cases increasing by 77.53% from 441.57 million in 1990 to 783.95 million in 2019.
- GERD is likely to remain a common reason for consultation in primary care, and our data may allow for health service provision planning.

## Introduction

Gastroesophageal reflux disease (GERD) is a common condition in which the reflux of gastric contents into the oesophagus results in symptoms and/or complications [1]. Typical GERD symptoms include heartburn and acid reflux. Histological changes in the oesophageal mucosa can be categorized into non-erosive reflux disease, reflux esophagitis, and Barrett's oesophagus [2]. GERD can also cause a variety of extraesophageal manifestations including asthma, cough, sore throat, throat clearing, and unexplained chest pain [3]. Due to its recurrent and troublesome symptoms and complications (i.e. oesophageal inflammation, stricture, ulceration, perforation, metaplasia, and oesophageal adenocarcinoma) [4], GERD seriously lowers patients' health-related quality of life. Besides, due to its chronic nature and high prevalence, GERD has imposed a significant economic burden on patients and their families, health services, and society [5]. The clinical management of GERD influences the lives of many individuals and is responsible for the substantial consumption of health care and societal resources [2].

Previous systematic reviews have presented the prevalence and incidence of GERD in the past decade [4, 6]. Based on the Global Burden of Disease (GBD) 2017 Study, GBD 2017 Gastro-oesophageal Reflux Disease Collaborators comprehensively described the global burden of GERD [7]. However, the burden of GERD varies over time and between or within countries and territories. Clinical trial suggests similar remission rates with surgery or medication [8]. However, two-thirds to 100% of patients will relapse when PPIs are stopped [9]. The global prevalence of GERD is increasing [3]. And the incidence of GERD has been increasing yearly due to improvements in living standards and changes in lifestyle and dietary habits recently [10]. These significant changes highlight the need for a comparable, consistent and systematic analysis of disease burden and trends concerning GERD in different regions and countries, which is critical to creating strategies for global intervention.

The GBD 2019 Study, an extensive worldwide observational epidemiological study to date, assesses the incidence, prevalence, and disability of 369 diseases by age, sex, location, and year [11], which presents an opportunity to better understand the epidemiology of GERD. In this article, we aimed to describe the burden of GERD, by age, sex, and social-development index (SDI), in 204 countries and territories from 1990 to 2019. The availability of estimates of disease burden for GERD would provide a better understanding of the impact of GERD on population health and the need for appropriate preventive strategies and health resource allocations.

## Methods

The detailed description of original data and general methodology of GBD 2019 study has been described in previous publications [11–14]. The study protocol and statistical codes of the estimated GERD can be obtained from the website: http://ghdx.healthdata.org/gbd-2019/code/cod-4. In brief, incidence and prevalence of disease were estimated using a wide range of data from representative population. These data were derived from literature reviews and identified through research collaborations, which included published scientific reports of registries and cohorts, data from cohort and registry studies, administrative health data and reports, and population surveys [12]. DisMod-MR 2.1, an epidemiologic state-transition disease modelling software, together with MR-BRT, a Bayesian meta-regression software, were used to produce consistent disease estimates [12]. Uncertainty intervals (UIs) were produced for every metric using the 25th and 975th ordered 1000 draw values of the posterior distribution [12].

Crude and age-standardized estimates of various measures of the burden of GERD in 204 countries and territories from 1990 to 2019 and the respective 95% UIs were extracted from the GBD database via http://ghdx.healthdata.org/gbd-results-tool and no specific permissions were required to access data. For the GBD 2019 assessment, GERD was claimed by the following codes according to the 10th revision of the International Classification of Diseases (ICD-10): K21-K21.9, K22.7, and R12 (Supplementary Table 1). The variables obtained from the database included incident cases, prevalent cases, YLDs numbers, and their corresponding age-standardized rates (ASRs) at the global, regional, and national levels. These data were stratified by age (5–9, every 5-year age group up to 95 years, and 95 years and older), calendar year (1990–2019), region, and country (or territory). Geographically, the world was divided into 21 GBD regions. Moreover, the 204 countries and territories were categorized into five groups in terms of their socio-demographic index (SDI): high, high-middle, middle, low-middle, and low SDI quintile [12].

Estimated average percentage change (EAPC) was computed to depict the secular trend in ASRs of GERD burden based on a regression model by fitting the natural logarithm of ASR with the calendar year, namely, *y* = *α* + *βx*+*ɛ*, where *y* = ln (rate), *x* = calendar year, and *ε* = error term. In this formula, *β* represents the positive or negative ASR trends. EAPC and its 95% confidence interval (CI) were calculated from the formula of 100 × (exp (*β*) −1) [15]. The age-standardized indicator was recognized to be in an increasing trend if the value of EAPC and the lower boundary of 95% CI were both greater than 0, to be a decreasing trend if EAPC value and the upper boundary of 95% CI were both less than 0, and to be a constant trend when 95% CI of EAPC included 0. All statistical analyses and visualizations were conducted using R statistical software program (version 4.1.0). *P*-value <.05 was considered statistically significant.

Because the study was based on publicly available dataset, this study was exempted by the ethics committee of the Third Xiangya Hospital of Central South University. Each step used to analyse the GBD database in the current study followed the guideline of cross-sectional study described in the Guidelines for Accurate and Transparent Health Estimates Reporting (GATHER) [16].

## Results

### Global burden and temporal trend in GERD

**Figure 1.**
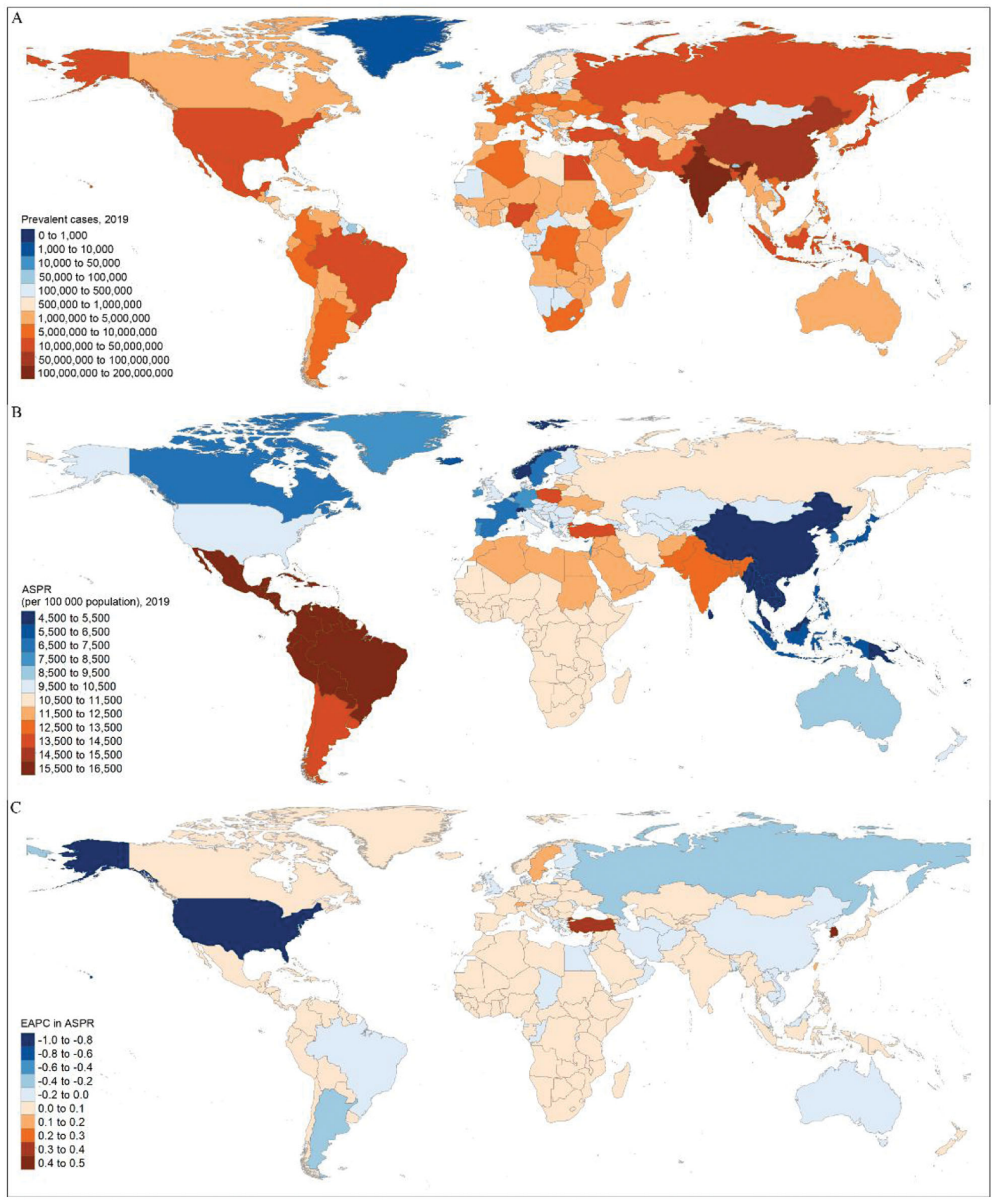
The global prevalence burden of GERD in 204 countries and territories. (a) The absolute number of GERD prevalent cases in 2019. (b) The ASPR (per 100,000 population) of GERD in 2019. (c) The EAPC of ASPR for GERD between 1990 and 2019. ASPR: age-standardized prevalence rate; EAPC: estimated annual percentage change; GERD: gastroesophageal reflux disease.

Globally, the number of prevalent cases of GERD increased by 77.53% from 441.57 million (95% UI 383.48– 496.84) in 1990 to 783.95 million (689.55–876.53) in 2019 (Supplementary Table 2 and Figure 1(a)). Besides, the age-standardized prevalence rate (ASPR) of GERD was 9344.52 (8213.69–10456.84) per 100,000 population in 1990 and 9574.45 (8416.42–10,698.38) per 100,000 population in 2019, with the EAPC being 0.04 (95% CI 0.00–0.08) (Table 1, Figure 1(b)). Meanwhile, the global incident cases of GERD were 309.38 million (272.53–349.51) in 2019, increasing from 177.00 million (154.83–201.15) in 1990, with an increasing EAPC in age-standardized incidence rate (ASIR) of 0.06 (0.02–0.10) from 3687.27 (3256.92–4165.86) in 1990–3792.79 (3341.66–4280.02) in 2019 per 100,000 population (Table 1 and Supplementary Table 2). Globally, there were 6.03 million (3.10–10.82) YLDs caused by GERD in 2019, which increased by 77.19% from 3.40 million (1.76–6.09) in 1990. The age-standardized YLD rate (ASYR) increased from 71.68 (36.95–128.64) in 1990 to 73.63 (38.03–132.08) in 2019 per 100,000 population with the EAPC being 0.05 (0.01–0.09) (Table 1 and Supplementary Table 2). Meanwhile, from 2015 to 2019, ASPR, ASIR, and ASYR all increased globally with the EAPC being 0.56 (0.30–0.82), 0.54 (0.30–0.79), and 0.56 (0.30–0.82), respectively (Supplementary Table 3).

**Table 1.** Age-standardized prevalence, incidence, and YLD rates for gastroesophageal reflux disease (GERD) in 1990 and 2019 and their temporal trends from 1990 to 2019.

Characteristics	Age-standardized prevalence rate per 100,000 population			Age-standardized incidence rate per 100,000 population			Age-standardized YLD rate per 100,000 population		
	1990 No. (95%UI)	2019 No. (95%UI)	EAPC No. (95%CI)	1990 No. (95%UI)	2019 No. (95%UI)	EAPC No. (95%CI)	1990 No. (95%UI)	2019 No. (95%UI)	EAPC No. (95%CI)
Global	9344.52 (8213.69 to 10,456.84)	9574.45 (8416.42 to 10,698.38)	0.04 (0.00 to 0.08)	3687.27 (3256.92 to 4165.86)	3792.79 (3341.66 to 4280.02)	0.06 (0.02 to 0.10)	71.68 (36.95 to 128.64)	73.63 (38.03 to 132.08)	0.05 (0.01 to 0.09)
Sex									
Male	8965.06 (7872.25 to 10,037.60)	9202.85 (8087.69 to 10,290.43)	0.06 (0.02 to 0.09)	3548.43 (3129.86 to 4016.03)	3655.00 (3218.54 to 4142.05)	0.08 (0.04 to 0.11)	69.08 (35.62 to 124.33)	71.11 (36.71 to 127.76)	0.07 (0.04 to 0.10)
Female	9719.69 (8537.36 to 10,915.06)	9941.90 (8717.74 to 11,155.47)	0.02 (−0.03 to 0.06)	3825.60 (3378.20 to 4311.11)	3929.78 (3465.46 to 4430.41)	0.05 (0.01 to 0.08)	74.25 (38.28 to 132.56)	76.13 (39.35 to 135.76)	0.07 (0.04 to 0.10)
SDI quintile									
High	8512.56 (7442.29 to 9594.13)	8252.43 (7193.12 to 9291.2)	−0.24 (−0.32 to −0.15)	3388.61 (2968.37 to 3866.89)	3319.50 (2906.37 to 3810.77)	−0.17 (−0.24 to −0.11)	65.37 (33.27 to 118.08)	63.40 (32.17 to 114.26)	−0.23 (−0.32 to −0.15)
High-middle	9014.77 (7927.55 to 10,110.23)	8700.91 (7677.19 to 9701.44)	−0.21 (−0.27 to −0.14)	3524.13 (3116.29 to 3994.07)	3410.01 (3011.96 to 3854.01)	−0.19 (−0.24 to −0.13)	69.26 (35.58 to 124.07)	67.09 (34.59 to 120.40)	−0.19 (−0.25 to −0.13)
Middle	8082.43 (7099.76 to 9064.77)	8720.16 (7652.32 to 9769.90)	0.24 (0.21 to 0.28)	3203.91 (2827.63 to 3614.98)	3461.20 (3052.34 to 3901.86)	0.25 (0.22 to 0.28)	62.16 (32.01 to 111.64)	67.19 (34.68 to 120.62)	0.25 (0.22 to 0.29)
Low-middle	11,773.99 (10,401.16 to 13,164.30)	11,887.87 (10,505.45 to 13,282.26)	0.02 (0.01 to 0.03)	4603.90 (4087.96 to 5177.94)	4650.41 (4124.90 to 5226.35)	0.03 (0.02 to 0.04)	89.83 (46.6 to 161.42)	90.99 (47.14 to 163.13)	0.03 (0.02 to 0.05)
Low	12,085.16 (10,597.10 to 13,536.65)	12,054.64 (10,577.33 to 13,501.86)	0.00 (−0.01 to 0.00)	4758.96 (4219.43 to 5379.93)	4741.79 (4202.29 to 5361.43)	−0.01 (−0.01 to −0.01)	92.18 (47.98 to 165.24)	92.25 (48.02 to 165.73)	0.01 (0.01 to 0.02)
GBD region									
Andean Latin America	15,934.38 (14,135.36 to 17,780.23)	15,932.06 (14,135.41 to 17,774.71)	0.00 (0.00 to 0.00)	5976.14 (5315.06 to 6644.79)	5974.25 (5312.88 to 6641.29)	0.00 (0.00 to 0.00)	123.03 (63.86 to 217.88)	123.15 (64.00 to 218.31)	0.00 (0.00 to 0.01)
Australasia	8694.45 (7579.92 to 9896.39)	8679.81 (7566.16 to 9881.92)	−0.02 (−0.16 to 0.12)	3516.72 (3073.84 to 4048.01)	3511.78 (3070.46 to 4043.23)	−0.01 (−0.12 to 0.09)	66.71 (34.03 to 120.31)	66.66 (34.06 to 121.04)	−0.02 (−0.16 to 0.13)
Caribbean	15,937.21 (14,138.44 to 17,780.66)	15,935.32 (14,137.14 to 17,777.84)	0.00 (0.00 to 0.00)	5976.80 (5315.24 to 6644.09)	5975.49 (5313.40 to 6642.33)	0.00 (0.00 to 0.00)	122.99 (64.07 to 217.41)	122.78 (64.04 to 216.84)	0.00 (−0.01 to 0.00)
Central Asia	10,482.56 (9135.45 to 11,801.76)	10,471.50 (9125.15 to 11,787.88)	0.00 (0.00 to 0.00)	4171.74 (3663.06 to 4766.35)	4168.45 (3658.11 to 4764.72)	0.00 (0.00 to 0.00)	80.89 (41.35 to 146.25)	80.81 (41.30 to 145.70)	0.00 (0.00 to 0.00)
Central Europe	10,863.54 (9520.36 to 12,246.17)	10,987.32 (9631.12 to 12,376.53)	0.05 (0.04 to 0.05)	4262.46 (3756.68 to 4830.14)	4301.11 (3789.35 to 4872.82)	0.04 (0.03 to 0.04)	83.43 (42.78 to 148.19)	84.64 (43.44 to 150.20)	0.06 (0.05 to 0.06)
Central Latin America	15,940.70 (14,148.00 to 17,600.10)	15,949.53 (14,156.95 to 17,601.72)	0.00 (0.00 to 0.00)	6039.93 (5379.89 to 6694.89)	6040.22 (5378.11 to 6691.82)	0.00 (0.00 to 0.00)	122.69 (63.80 to 218.43)	122.92 (63.83 to 218.74)	0.00 (0.00 to 0.01)
Central Sub-Saharan Africa	11,036.02 (9606.30 to 12,450.75)	11,035.93 (9621.69 to 12,451.49)	0.00 (0.00 to 0.00)	4388.20 (3855.17 to 5009.03)	4388.34 (3853.19 to 5007.99)	0.00 (0.00 to 0.00)	84.13 (43.69 to 149.18)	84.58 (43.95 to 151.08)	0.02 (0.02 to 0.02)
East Asia	4542.27 (3937.00 to 5164.94)	4523.40 (3912.03 to 5149.59)	−0.14 (−0.29 to 0.00)	1853.82 (1617.20 to 2149.28)	1847.31 (1612.24 to 2139.60)	−0.13 (−0.26 to 0.01)	35.12 (17.82 to 63.31)	35.05 (17.78 to 63.23)	−0.13 (−0.28 to 0.01)
Eastern Europe	11,356.33 (9995.84 to 12,756.35)	11,331.34 (9974.89 to 12,728.49)	−0.21 (−0.33 to −0.10)	4487.10 (3948.05 to 5093.00)	4478.85 (3938.02 to 5080.20)	−0.17 (−0.26 to −0.08)	86.98 (44.47 to 157.52)	87.11 (44.45 to 157.69)	−0.20 (−0.31 to −0.08)
Eastern Sub-Saharan Africa	11,191.12 (9742.00 to 12,561.77)	11,199.13 (9750.44 to 12,572.49)	0.00 (0.00 to 0.00)	4451.93 (3906.53 to 5069.11)	4454.59 (3909.59 to 5068.92)	0.00 (0.00 to 0.00)	85.62 (44.37 to 152.89)	86.02 (44.60 to 154.04)	0.02 (0.02 to 0.02)
High-income Asia Pacific	6119.10 (5333.23 to 6997.03)	6225.69 (5431.16 to 7113.27)	0.25 (0.12 to 0.37)	2532.38 (2208.26 to 2926.00)	2572.68 (2244.20 to 2974.08)	0.20 (0.10 to 0.30)	47.29 (24.05 to 85.78)	48.25 (24.60 to 87.46)	0.25 (0.13 to 0.38)
High-income North America	10614.30 (9254.75 to 11,985.62)	9401.60 (8190.07 to 10,614.97)	−0.82 (−1.02 to −0.63)	4095.69 (3560.93 to 4654.85)	3727.97 (3253.14 to 4285.69)	−0.66 (−0.81 to −0.50)	81.07 (41.69 to 145.33)	71.62 (36.26 to 128.42)	−0.83 (−1.03 to −0.64)
North Africa and Middle East	12,240.02 (10,688.33 to 13,786.42)	12,341.45 (10,898.84 to 13,766.4)	0.03 (0.01 to 0.05)	4781.05 (4214.49 to 5396.43)	4799.43 (4259.75 to 5391.27)	0.01 (−0.01 to 0.03)	94.30 (48.44 to 170.89)	95.07 (48.91 to 170.82)	0.03 (0.01 to 0.05)
Oceania	5283.72 (4563.88 to 6047.55)	5284.59 (4563.20 to 6048.44)	0.00 (0.00 to 0.00)	2164.68 (1891.94 to 2493.36)	2165.05 (1892.06 to 2495.02)	0.00 (0.00 to 0.00)	40.54 (20.70 to 73.93)	40.48 (20.57 to 73.75)	0.00 (0.00 to 0.00)
South Asia	13,377.44 (11,777.46 to 14,977.74)	13,377.84 (11,769.10 to 14,984.49)	0.00 (−0.01 to 0.01)	5222.37 (4632.67 to 5883.96)	5223.34 (4636.37 to 5882.55)	0.00 (−0.01 to 0.01)	101.66 (52.54 to 183.28)	102.04 (52.78 to 183.85)	0.01 (0.00 to 0.03)
Southeast Asia	5432.59 (4716.44 to 6211.09)	5427.51 (4711.75 to 6203.74)	0.00 (0.00 to 0.00)	2213.29 (1932.35 to 2562.26)	2211.43 (1931.03 to 2560.16)	0.00 (0.00 to 0.00)	41.77 (21.23 to 75.63)	41.85 (21.26 to 76.10)	0.01 (0.01 to 0.01)
Southern Latin America	13,628.01 (11,862.59 to 15,357.49)	13,625.51 (11,860.68 to 15,353.48)	−0.15 (−0.21 to −0.09)	5003.17 (4406.68 to 5687.32)	5002.39 (4405.74 to 5686.77)	−0.09 (−0.13 to −0.06)	105.21 (54.14 to 189.23)	105.12 (54.12 to 187.46)	−0.15 (−0.21 to −0.09)
Southern Sub-Saharan Africa	11,369.36 (9899.33 to 12,811.40)	11,379.36 (9906.25 to 12,826.20)	0.00 (0.00 to 0.00)	4522.20 (3978.59 to 5137.46)	4524.95 (3983.00 to 5139.59)	0.00 (0.00 to 0.00)	87.11 (45.02 to 155.84)	86.82 (44.80 to 155.01)	−0.01 (−0.01 to −0.01)
Tropical Latin America	16,335.09 (14,509.64 to 18,040.17)	16,207.50 (14,319.22 to 17,944.65)	−0.12 (−0.16 to −0.08)	6164.75 (5485.23 to 6806.08)	6144.66 (5455.45 to 6787.85)	−0.07 (−0.09 to −0.04)	125.29 (65.30 to 221.80)	124.78 (64.60 to 222.07)	−0.11 (−0.15 to −0.07)
Western Europe	8218.38 (7188.66 to 9247.73)	8217.57 (7182.45 to 9250.17)	0.02 (0.01 to 0.03)	3304.06 (2909.15 to 3774.92)	3303.30 (2898.46 to 3775.10)	0.02 (0.00 to 0.03)	63.26 (32.24 to 113.81)	63.30 (32.30 to 114.14)	0.02 (0.01 to 0.03)
Western Sub-Saharan Africa	11,224.51 (9779.71 to 12,643.62)	11,236.58 (9782.82 to 12,626.62)	0.00 (0.00 to 0.01)	4465.94 (3929.26 to 5079.05)	4469.45 (3926.83 to 5086.96)	0.00 (0.00 to 0.00)	86.14 (44.53 to 154.02)	86.45 (44.83 to 154.98)	0.02 (0.01 to 0.02)

Abbreviations: CI: confidence interval; EAPC, estimated annual percentage change; GBD: Global Burden of Disease; SDI: socio-demographic index; UI: uncertainty interval; YLD: years of life lived with disability.

### Variation in GERD burden at regional level

**Figure 2.**
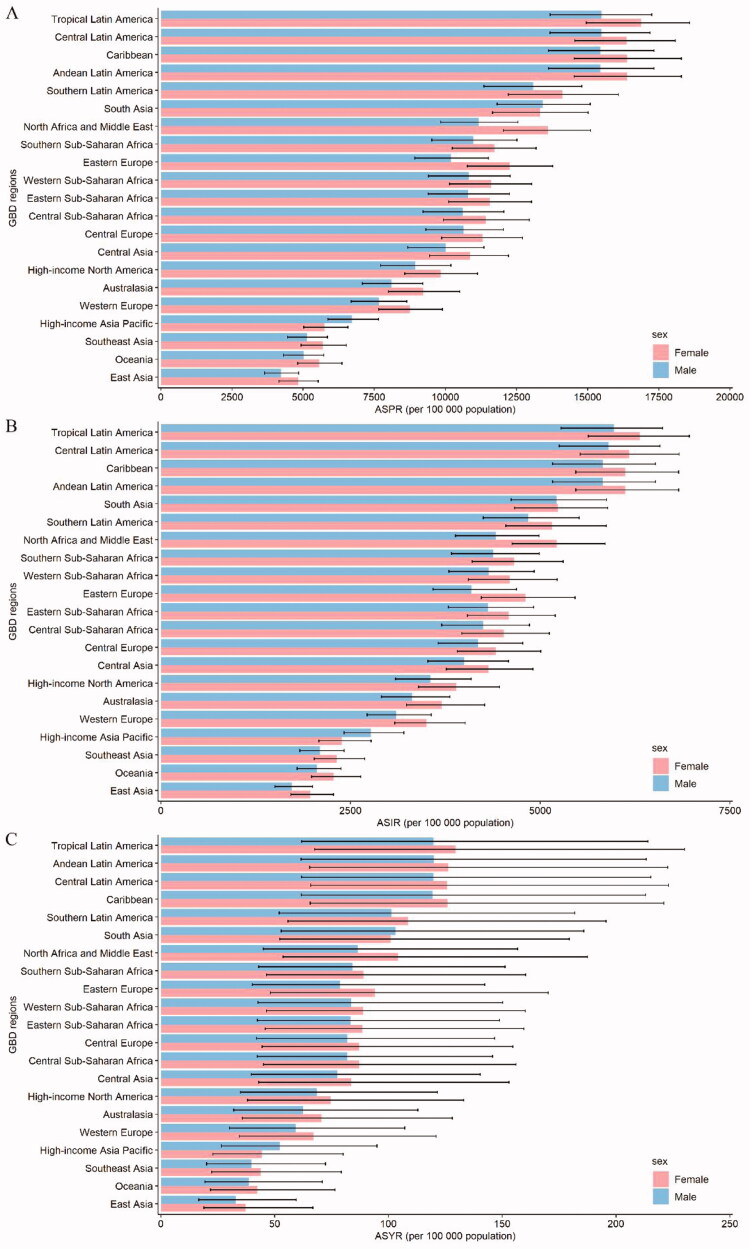
The ASPR (a), ASIR (b), and ASYR (c) due to GERD by sex, across 21 GBD regions, in 2019. Error bars indicate the 95% uncertainty interval (UI) for the age-standardized rates. ASIR: age-standardized incidence rate; ASPR: age-standardized prevalence rate; ASYR: age-standardized YLD rate; GBD: Global Burden of Disease; GERD: gastroesophageal reflux disease.

**Figure 3.**
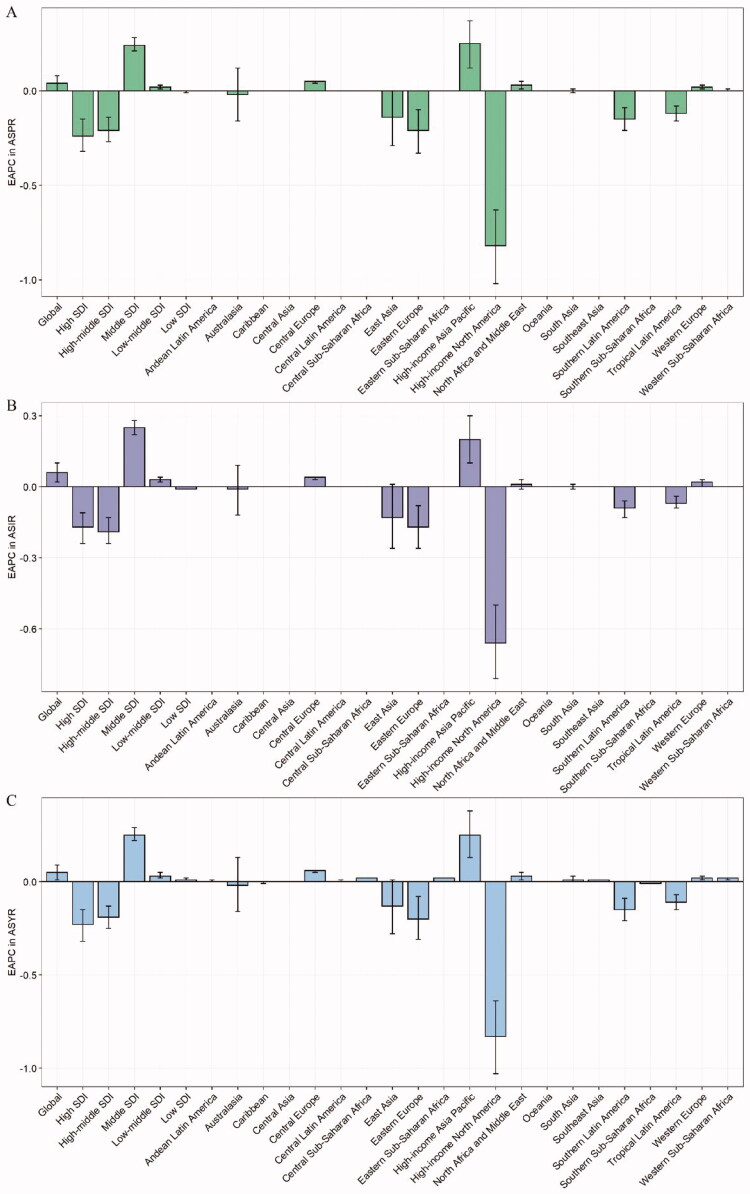
The EAPCs in ASPR (a), ASIR (b), and ASYR (c) due to GERD from 1990 to 2019, both sexes, by GBD region, and by SDI quintile. Error bars indicate the 95% confidence interval (CI) for the rates. ASIR: age-standardized incidence rate; ASPR: age-standardized prevalence rate; ASYR: age-standardized YLD rate; EAPC: estimated annual percentage change; GBD: Global Burden of Disease; GERD: gastroesophageal reflux disease; SDI: socio-demographic index.

Regarding GBD regions, South Asia, East Asia, and North Africa and Middle East were among the top three regions for the largest numbers of prevalent cases, incident cases, and YLDs in 2019 (Supplementary Table 2). On the contrary, the lowest numbers of prevalent cases, incident cases, and YLDs in 2019 were observed in Oceania, Australasia, and Caribbean. Tropical Latin America was the region with the highest ASPR [16,207.50 (95% UI 14,319.22–17,944.65)], ASIR [6144.66 (5455.45–6787.85)], and ASYR [124.78 (64.60–222.07)] in 2019 (Table 1 and Figure 2). East Asia had the lowest ASPR [4523.40 (3912.03–5149.59)], ASIR [1847.31 (1612.24–2139.60)], and ASYR [35.05 (17.78–63.23)] among regions in 2019. Moreover, ASPR and ASIR remained stable over 30 years in almost half of all the regions. However, total prevalent cases, incident cases, and YLDs increased across all GBD regions during the observation period (Supplementary Figures 1–3). The EAPCs in ASPR, ASIR, and ASYR were highest in High-income Asia Pacific (EAPC = 0.25, 0.20, and 0.25, respectively) and lowest in High-income North America (EAPC = −0.82, −0.66, and −0.83, respectively) from 1990 to 2019 (Figure 3). However, from 2015 to 2019, the EAPCs in ASPR, ASIR, and ASYR were highest in Eastern Europe (EAPC = 1.74, 1.41, and 1.73, respectively), and the EAPC in ASPR was lowest in North Africa and Middle East (EAPC = −0.07), the EAPCs in ASIR and ASYR were lowest in Western Europe (EAPC = −0.08 and −0.13, respectively) (Supplementary Table 3).

### Variation in GERD burden at national and territorial level

At the national and territorial level in 2019, India and China had the highest number of prevalent cases [181.55 million (158.49–204.38), 81.64 million (70.73–93.51), respectively], the highest number of incident cases [71.84 million (95% UI 63.58–81.35), 32.71 million (28.44–38.21), respectively], and the highest number of YLD cases [1.39 million (0.72–2.49), 0.63 million (0.32–1.15), respectively] (Supplementary Table 4, Figure 1, and Supplementary Figures 4 and 5). In 2019, the highest ASIR of GERD was observed in Brazil [6146.60 (5457.64–6790.57) per 100,000 population], and the highest ASPR and ASYR were found in Paraguay [16,310.51 (14,395.46–18,167.8) per 100,000 population, 125.91 (65.11–223.26) per 100,000 population, respectively] (Supplementary Table 5 and Supplementary Figures 6 and 7). In contrast, the lowest ASPR, ASIR, and ASYR were found in China [4509.32 (3899.11–5133.17) per 100,000 population, 1841.66 (1607.09–2133.51) per 100,000 population, and 34.94 (17.73–63.02) per 100,000 population, respectively]. Between 1990 and 2019, ASPR and ASIR remained stable in more than 60% countries or territories. Republic of Korea, Turkey, and Taiwan (Province of China) ranked in the top three countries or territories experienced a significant increase in ASPR (EAPC = 0.48, 0.30, and 0.19, respectively), ASIR (EAPC = 0.35, 0.17, and 0.14, respectively), and ASYR (EAPC = 0.49, 0.29, and 0.19, respectively), while United States of America, Russian Federation, and Argentina ranked in the top three countries or territories experienced a significant decrease in ASPR (EAPC = −0.89, −0.31, and −0.22, respectively), ASIR (EAPC = −0.71, −0.25, and −0.14, respectively), and ASYR (EAPC = −0.90, −0.30, and −0.23, respectively) (Figure 1(c) and Supplementary Figures 8 and 9). Nevertheless, from 2015 to 2019, Russian Federation, Bangladesh, and Kuwait ranked in the top three countries or territories experienced a significant increase in ASPR (EAPC = 2.62, 1.32, and 0.10, respectively), ASIR (EAPC = 2.11, 0.88, and 0.09, respectively), and ASYR (EAPC = 2.61, 1.33, and 0.07, respectively) (Supplementary Table 6).

### Variation in GERD burden in two sexes and five-year age groups

**Figure 4.**
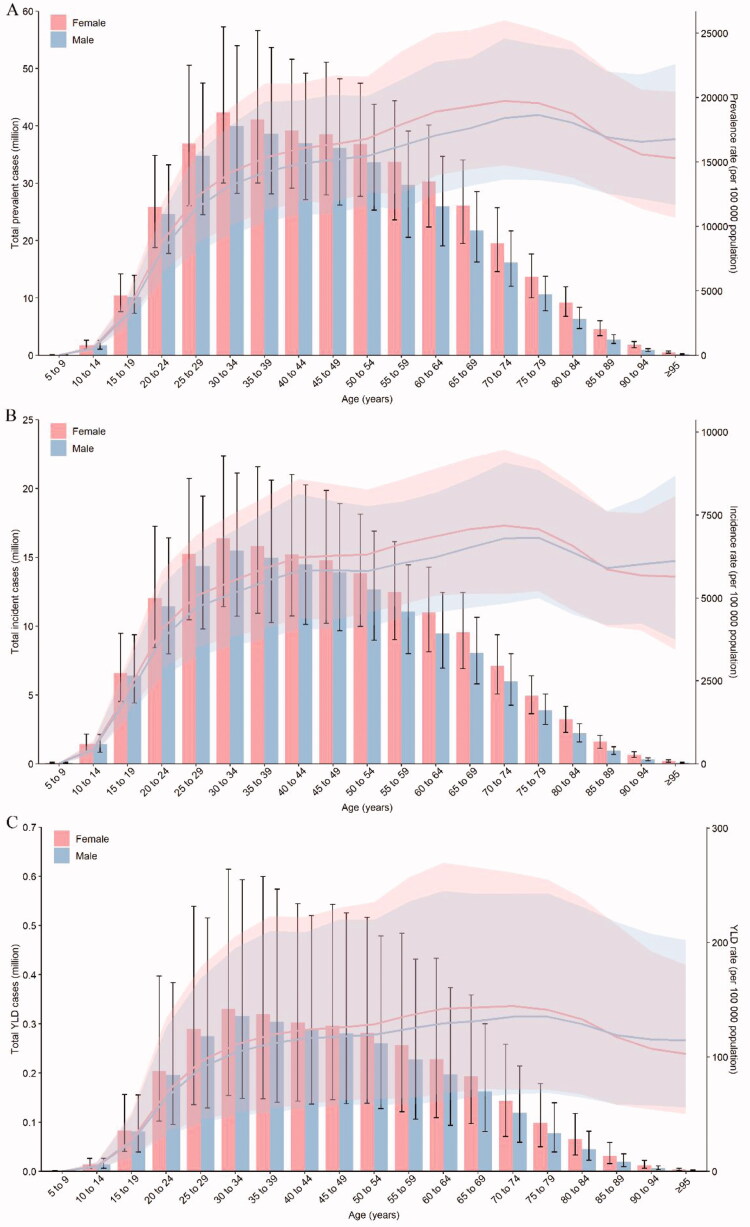
Age patterns by sex of the total number and age-specific prevalence rate (a), age-specific incidence rate (b), and age-specific YLD rate (c) due to GERD at the global level in 2019. Error bars indicate the 95% uncertainty interval (UI) for the number of cases. Shading indicates the 95% UI for the rates. GERD: gastroesophageal reflux disease.

Overall, the global number of prevalent cases, incident cases, and YLDs was higher in females than males in 2019. The ASPR, ASIR, and ASYR were also higher in females than males (Supplementary Table 2). In 2019, the number of prevalent cases, incident cases, and YLDs peaked in the 30–34 years age group in both sexes (Figure 4). The highest peak of GERD ASPR and ASIR occurred at age 75–79 years in males and 70–74 years in females in 2019. And the highest ASYR was observed at age 70–74 years for both sexes. From 1990 to 2019, the number of prevalent cases, incident cases, and YLDs continued to increase in both sexes and was higher in females than males in all years. The ASIR and ASYR both increased in males [EAPC = 0.08 (95% CI 0.04–0.11), EAPC = 0.07 (0.04–0.10), respectively] and in females [EAPC = 0.05 (0.01–0.08), EAPC = 0.07 (0.04–0.10), respectively] (Table 1). However, the ASPR increased in males [EAPC = 0.06 (0.02–0.09)] and remained stable in females [EAPC = 0.02 (−0.03 – 0.06)]. Meanwhile, from 2015 to 2019, the EAPCs in ASPR, ASIR, and ASYR all increased in both males and females (Supplementary Table 3).

### Variation in GERD burden by SDI

In 2019, higher SDI was associated with lower ASPR, ASIR, and ASYR of GERD, with values that were lower than the global rate in high, high-middle, and middle SDI quintiles, and higher than the global rate in the other two SDI quintiles (Table 1). High SDI quintile had the lowest ASPR, ASIR, and ASYR in 2019. Between 1990 and 2019, ASPR, ASIR, and ASYR decreased in high and high-middle quintiles, and increased in middle and low-middle SDI quintiles, whereas in low SDI quintile, ASIR decreased [EAPC = −0.01 (95% CI −0.01 – −0.01)], ASPR remained stable [EAPC = 0.00 (−0.01 – 0.00)], and ASYR increased [EAPC = 0.01 (0.01–0.02)]. Between 2015 and 2019, ASPR, ASIR, and ASYR decreased in low SDI quintile, but they all increased in the other four SDI quintiles (Supplementary Table 3).

**Figure 5.**
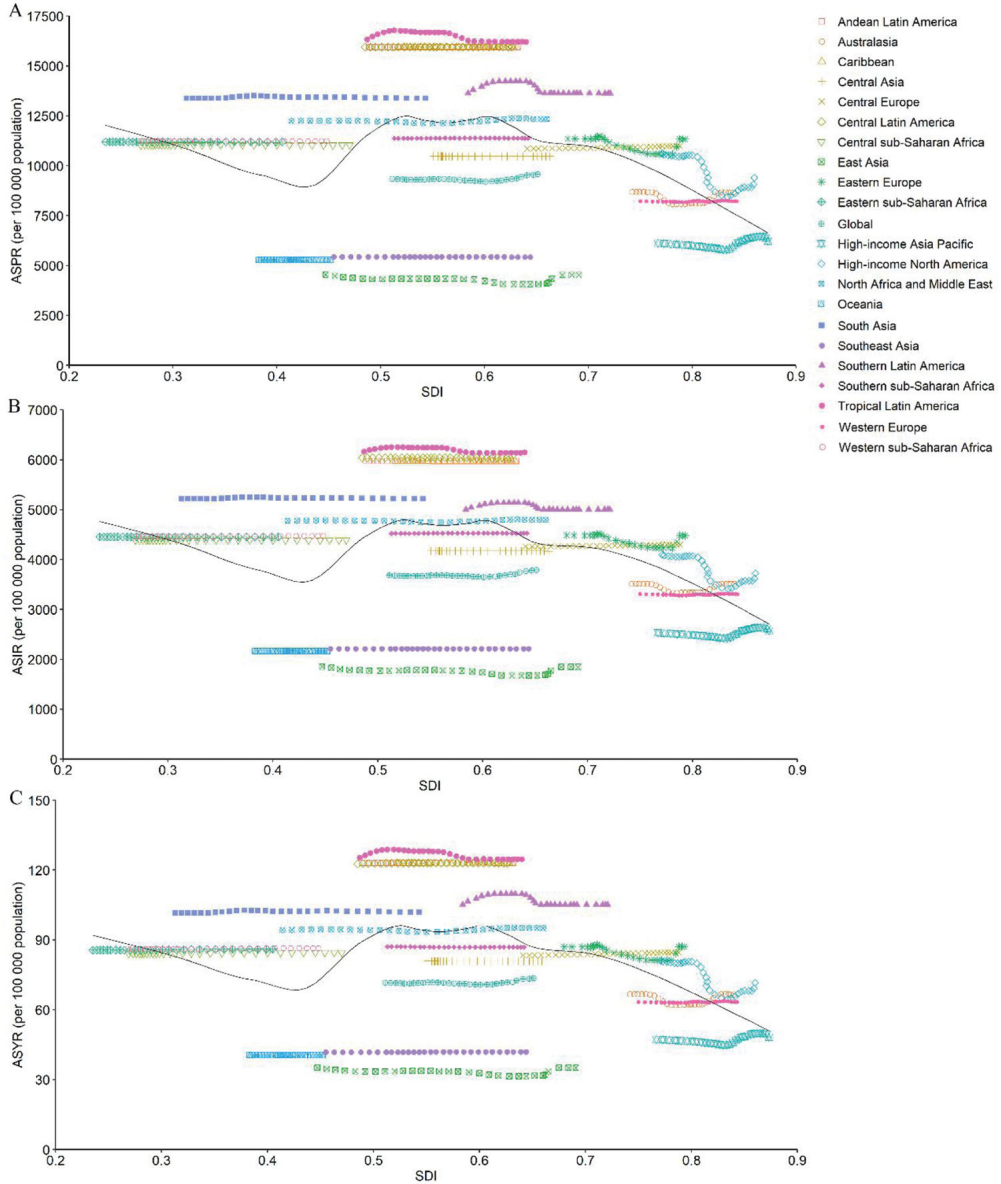
The ASPR (a), ASIR (b), and ASYR due to GERD globally and for 21 GBD regions by SDI from 1990 to 2019. The expected age-standardized rates in 2019 based solely on SDI were represented by the black line. For each region, points from left to right depict estimates from each year from 1990 to 2019. ASIR: age-standardized incidence rate; ASPR: age-standardized prevalence rate; ASYR: age-standardized YLD rate; GBD: Global Burden of Disease; GERD: gastroesophageal reflux disease; SDI: socio-demographic index.

The observed global and regional ASPR, ASIR, and ASYR in relation to SDI, versus the expected level for each location on the basis of SDI, are shown in Figure 5, which are expressed in the annual time series from 1990 to 2019. Except for High-income North America, the ASPR, ASIR, and ASYR in most GBD regions presented little change with increasing SDI values. At the global level, the ASPR, ASIR, and ASYR climbed slightly with increasing SDI values but under the expected levels during the past 30 years.

## Discussion

In this study, we comprehensively analysed the trend in gastroesophageal reflux disease (GERD) burden in 204 countries and territories for the past two decades, and compared the trend between the last 20 years and 5 years. GBD 2019 incorporated more studies, providing the most updated estimates on GERD epidemiology. Besides, GBD 2019 made adjustments for case ascertainment and updated data sources to further improve data reliability. After these adjustments, our study confirmed that the overall burden continued to worsen with the prevalent cases increasing by 77.53% from 441.57 million in 1990 to 783.95 million in 2019. Although the age-standardized prevalence (ASPR) rate was stabilized globally, it increased in certain countries and territories. This changing trend could make implications for researchers and policy-makers to prepare clinicians and health care systems to prioritize management strategies.

In GBD 2019, the highest ASPR was observed in Latin America, the Caribbean, South Asia, North Africa and the Middle East (>12,000 cases per 100,000 population). East Asia and China had the lowest ASPR below 5%. Estimated average percentage change (EAPC) was used to evaluate the changing trend. EAPCs in age-standardized rates were highest in High-income Asia Pacific and lowest in High-income North America between 1990 and 2019. However, from 2015 to 2019, the EAPCs were highest in Eastern Europe, and the EAPC in ASPR was lowest in North Africa and Middle East, the EAPCs in ASIR and ASYR were lowest in Western Europe. The increasing disease burden in these regions could be associated with risk factors, including obesity, alcohol, and smoking [6].

There are previous studies on GERD burden. At the global level, GBD 2017 also reported stabilized ASPR and ASYR, without estimating the trend in ASIR from 1990 to 2017 [7]. Our data reported stabilized ASPR, and increased ASYR and ASIR from 1990 to 2019. ASPR, ASIR and ASYR all increased globally between 2015 and 2019. These data demonstrated the increasing trend of GERD burden in recent years. It was likely related to the obesity epidemic, which increased the odds up to 3-fold [17], and the decreasing prevalence of Helicobacter pylori-associated gastritis [4]. Eusebi et al. [6] reported higher estimates in America and the Middle East and lower estimates in East Asia. The estimate of East Asia was substantially below 5% in our study and several other studies. A survey showed the prevalence in five regions in China ranged from 1.7% to 5.1%, and Shanghai at 6.4% [18,19]. Another study reported an increase in Korea [20]. The prevalence in the US previously reported ranged from 10% to 30% [21–23], which was generally higher than GBD 2019 reported. The estimates previously reported were largely based on the population in Olmsted County or employed polulation [21, 24,25], which was less representative of the current US demographics, or potential responders who have GI symptoms [22]. GBD 2019 has incorporated more studies after and is more generalized. Besides, the criteria in some previous studies [21] were relatively generous as heartburn or regurgitation for at least one day per week. The GERD definition used in our study is from expert recommendation [26] and is consistent with a previous study [6], at least once per week for 12 months, overcoming the potential overestimation. Overall, our data were largely in line with previous studies.

Our study showed that advancing age was associated with an increased risk of GERD, and women had slightly higher rates of GERD, which was consistent with a previous study [6]. Eusebi et al. reported an effect of the geographical region on the association between GERD and gender, and it is more likely to be observed in women from South America, Southeast Asia, and the Middle East. Advancing age was non-constantly associated with GERD symptoms [4].

There are several limitations and strengths. One of the limitations is significant heterogeneity between studies included. There are cultural, ethnic, and geographic differences in the same region between different studies [27]. High-quality studies well-matched in location and population should be performed in future rounds. Moreover, the variability in study design and data collection methods also affects the precision of the estimates. Prevalence could be higher when the survey focussed on gastrointestinal symptoms, or through a postal questionnaire [6, 22]. This is only partially compensated in our analysis model. Furthermore, diagnostic criteria have a significant impact like mentioned before [28]. Our study used criteria consistent with expert consensus recommendations and a previous meta-analysis. Our data are largely consistent with previous studies, suggesting the validation of our study. Additionally, the association between different risk factors and GERD should be included in the future.

To our knowledge, this is the most updated estimate on GERD epidemiology globally, including 204 countries some of which were not been assessed before. EAPC was used to describe the trend over time. YLDs were used to quantitatively describe the negative impact of GERD on patients’ life and potentially could be an indicator of disease control. It included more studies and compensated for the study heterogeneity. Hence, the data reported should be more representative of individuals.

GERD remains to have a significant impact on the economic burden as a result of increasing prevalent cases globally. ASPR was stabilized globally, and ASIR and ASYR were slightly increased. Due to the negative impact of GERD on patients’ life quality [29], the association between GERD and increased risk of oesophageal adenocarcinoma [2, 30] and potential side effects of PPIs usage in treatment[31], More research on exploring certain risk factors, pathophysiology, and novel therapeutic strategies of GERD should be performed in the future.

## Supplementary Material

Supplemental Material

## Data Availability

The data were obtained through an online query tool from the website of IHME (http://ghdx.healthdata.org/), and no permissions were required to access the data.

## Abbreviations

EAPC: estimated annual percentage change
GERD: gastroesophageal reflux disease
SDI: socio-demographic index
GBD: Global Burden of Disease, Injuries, and Risk Factors Study
YLDs: years of life lived with disability
IHME: Institute for Health Metrics and Evaluation
GATHER: Guidelines for Accurate and Transparent Health Estimates Reporting
ASR: age-standardized rate
ASIR: age-standardized incidence rate
ASPR: age-standardiZed prevalence rate
ASYR: age-standardiZed YLD rate
UI: uncertainty interval
CI: confidence interval.
