# Supplementary material for: Global, regional and national burden of gastroesophageal reflux disease, 1990–2019: update from the GBD 2019 study
Source: Ann Med. 2022 May 17;54(1):1372–84. doi: 10.1080/07853890.2022.2074535 (PMC9122392; doi:10.1080/07853890.2022.2074535)
Supplement: Supplemental Material [file IANN_A_2074535_SM1739.docx]

Supplement to: **Global, regional and national burden of gastroesophageal reflux disease, 1990-2019: update from the GBD 2019 study**

**Contents:**

**Supplementary Figures**

Supplementary Figure 1: The absolute number of prevalent cases due to GERD for all GBD regions from 1990 to 2019.

Supplementary Figure 2: The absolute number of incident cases due to GERD for all GBD regions from 1990 to 2019.

Supplementary Figure 3: The absolute number of YLDs cases due to GERD for all GBD regions from 1990 to 2019.

Supplementary Figure 4: The absolute number of incident cases due to GERD for 204 countries and territories in 2019.

Supplementary Figure 5: The absolute number of YLD cases due to GERD for 204 countries and territories in 2019.

Supplementary Figure 6: The ASIR (per 100 000 population) of GERD for 204 countries and territories in 2019.

Supplementary Figure 7: The ASYR (per 100 000 population) of GERD for 204 countries and territories in 2019.

Supplementary Figure 8: The EAPC of ASIR for GERD in 204 countries and territories between 1990 and 2019.

Supplementary Figure 9: The EAPC of ASYR for GERD in 204 countries and territories between 1990 and 2019.

**Supplementary Tables**

Supplementary Table 1: Gastroesophageal reflux disease included in GBD 2019.

Supplementary Table 2: Prevalent cases, incident cases, and years of life lived with disability (YLDs) of gastroesophageal reflux disease (GERD) in 1990 and 2019 and their percentage change from 1990 to 2019

Supplementary Table 3 EAPC of age-standardised prevalence, incidence, and YLD rates for gastroesophageal reflux disease (GERD) from 2015 to 2019.

Supplementary Table 4: Prevalent cases, incident cases, and YLDs for gastroesophageal reflux disease (GERD) by 204 countries or territories in 2019 and 2019, and their percentage change from 1990 to 2019.

Supplementary Table5: Age-standardised prevalence, incidence, and YLD rates for gastroesophageal reflux disease (GERD) by 204 countries or territories in 1990 and 2019, and their temporal trends from 1990 to 2019.

Supplementary Table 6: EAPC of age-standardised prevalence, incidence, and YLD rates for gastroesophageal reflux disease (GERD) by 204 countries or territories from 2015 to 2019.

Supplementary Table 7: SDI quintile in 2019.

**Supplementary Figure 1.** The absolute number of prevalent cases due to GERD for all GBD regions from 1990 to 2019.


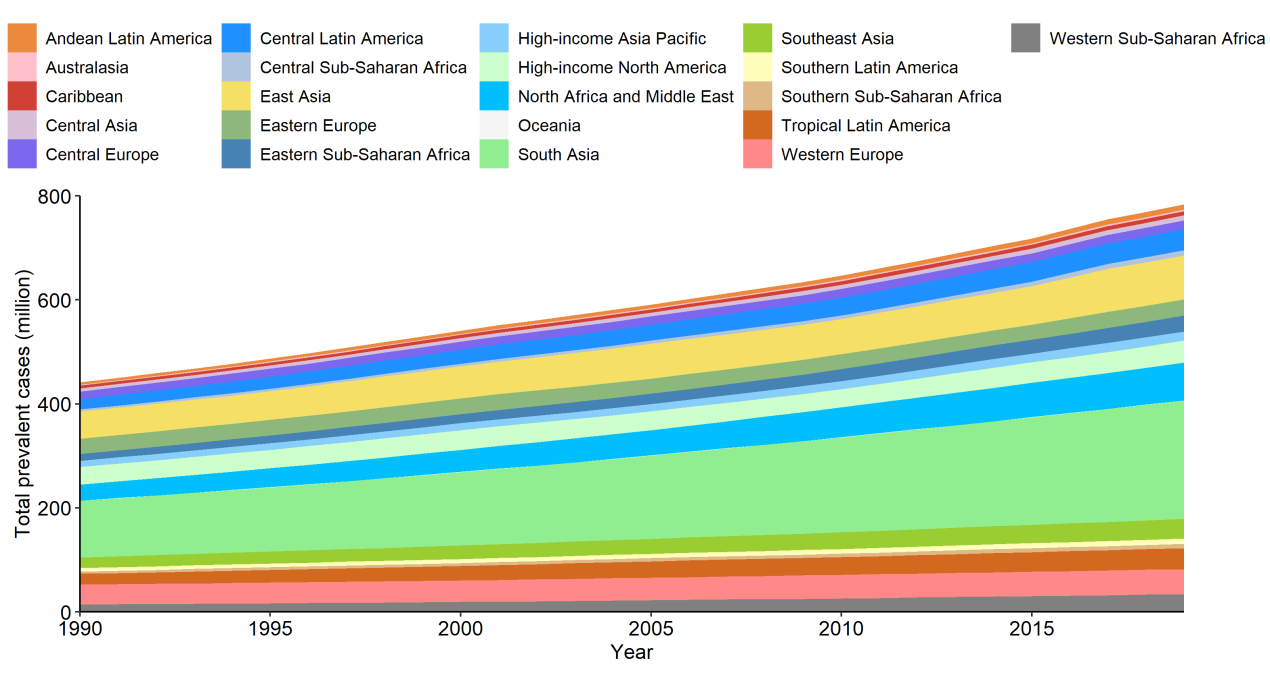


GBD, Global Burden of Disease; GERD, gastroesophageal reflux disease.

**Supplementary Figure 2.** The absolute number of incident cases due to GERD for all GBD regions from 1990 to 2019.


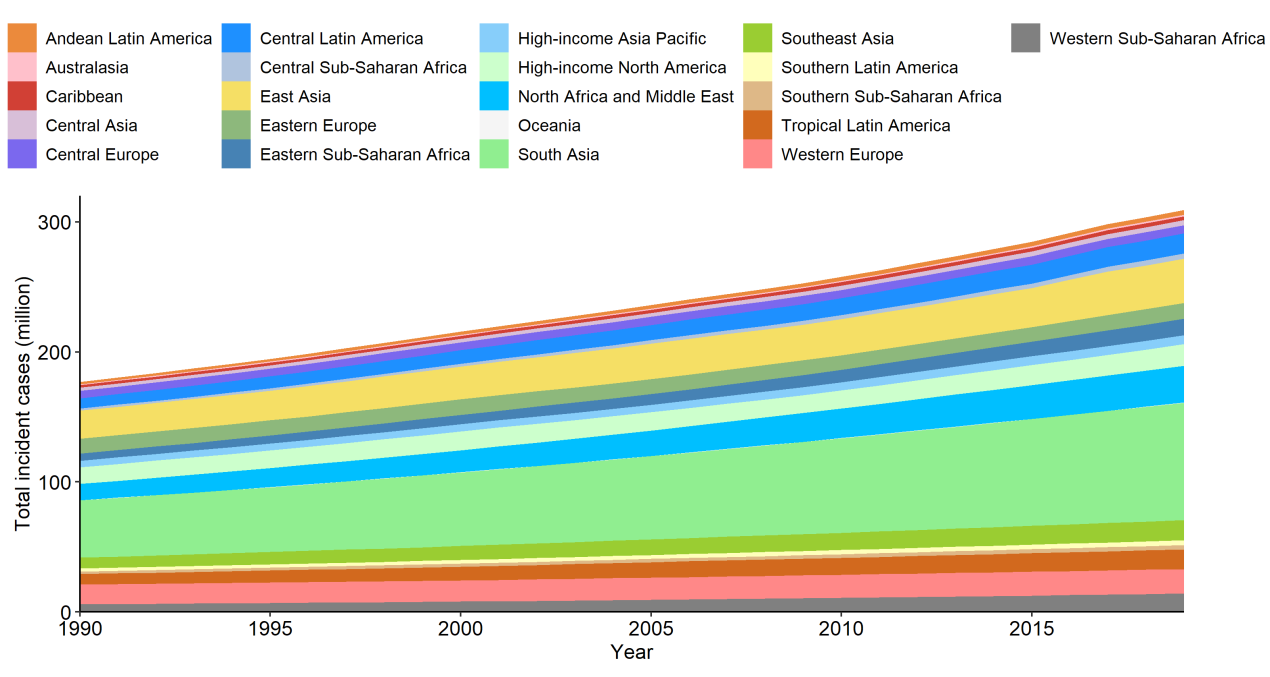


GBD, Global Burden of Disease; GERD, gastroesophageal reflux disease.

**Supplementary Figure 3.** The absolute number of YLDs cases due to GERD for all GBD regions from 1990 to 2019.


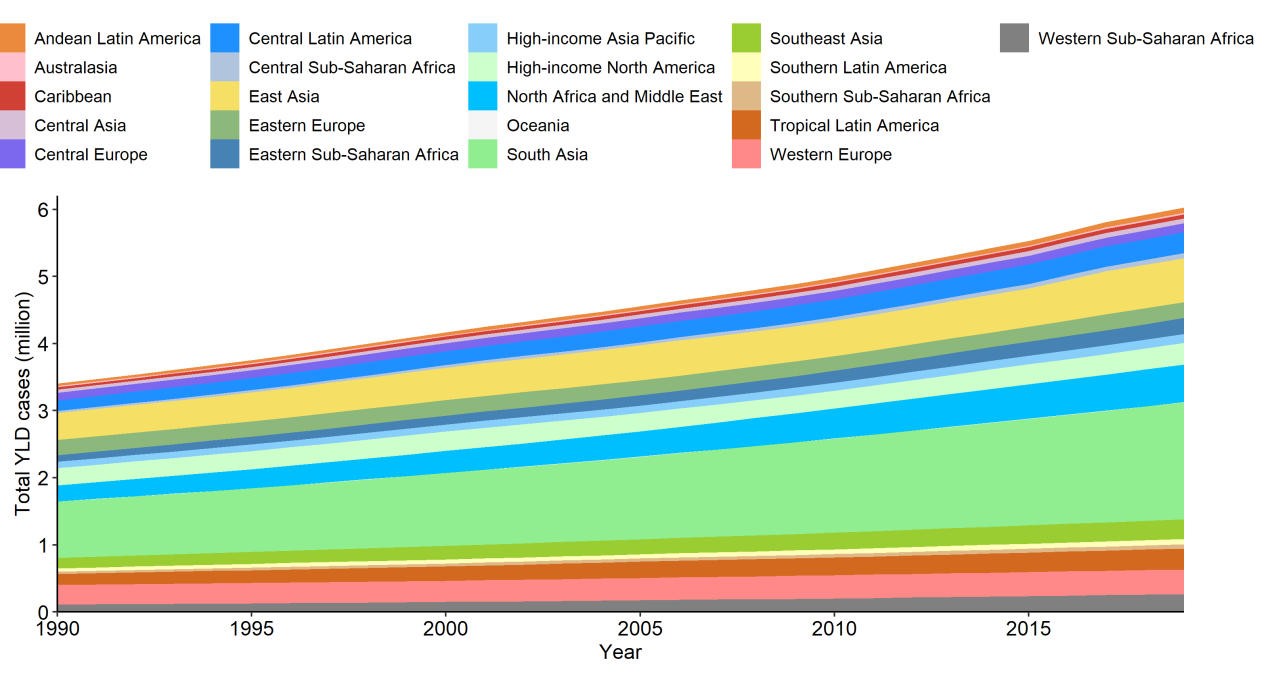


GBD, Global Burden of Disease; GERD, gastroesophageal reflux disease; YLD, year of life lived with disability.

**Supplementary Figure 4.** The absolute number of incident cases due to GERD for 204 countries and territories in 2019.


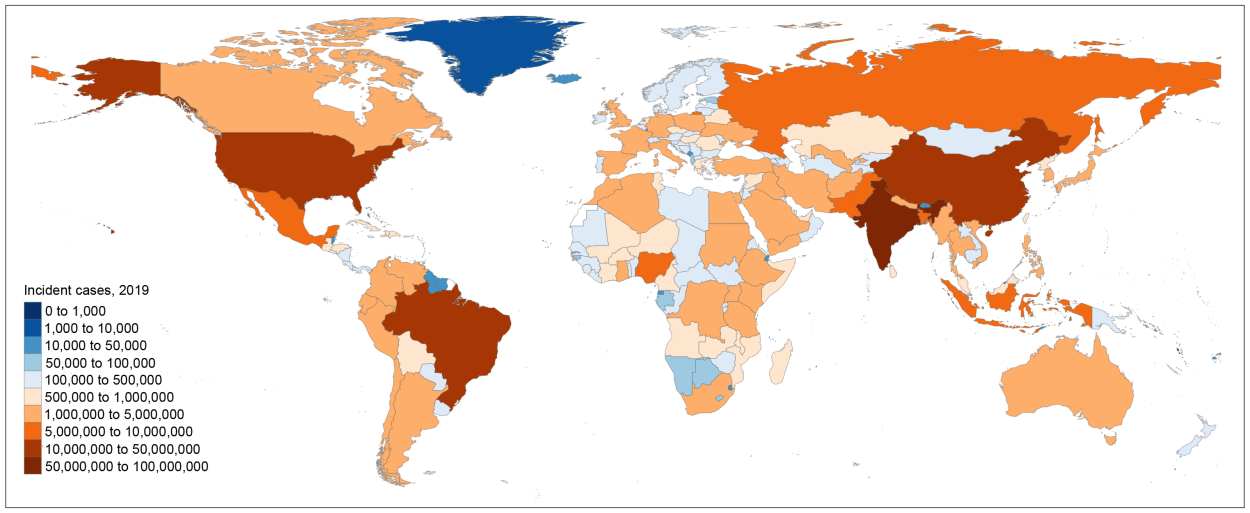


GERD, gastroesophageal reflux disease.

**Supplementary Figure 5.** The absolute number of YLD cases due to GERD for 204 countries and territories in 2019.


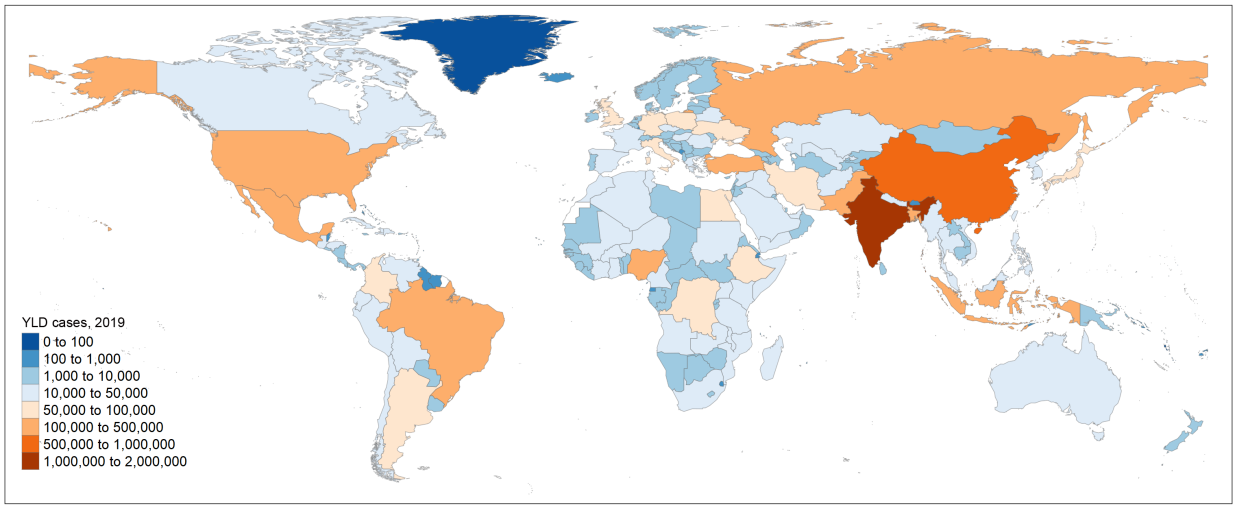


GERD, gastroesophageal reflux disease; YLD, year of life lived with disability.

**Supplementary Figure 6.** The ASIR (per 100 000 population) of GERD for 204 countries and territories in 2019.


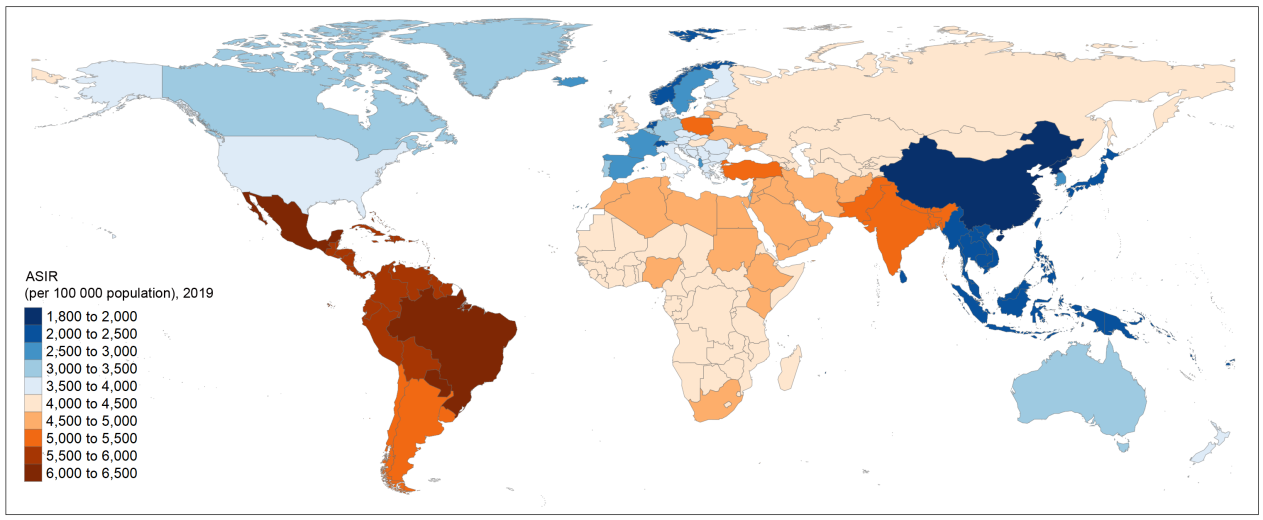


ASIR, age-standardised incidence rate; GERD, gastroesophageal reflux disease.

**Supplementary Figure 7.** The ASYR (per 100 000 population) of GERD for 204 countries and territories in 2019.


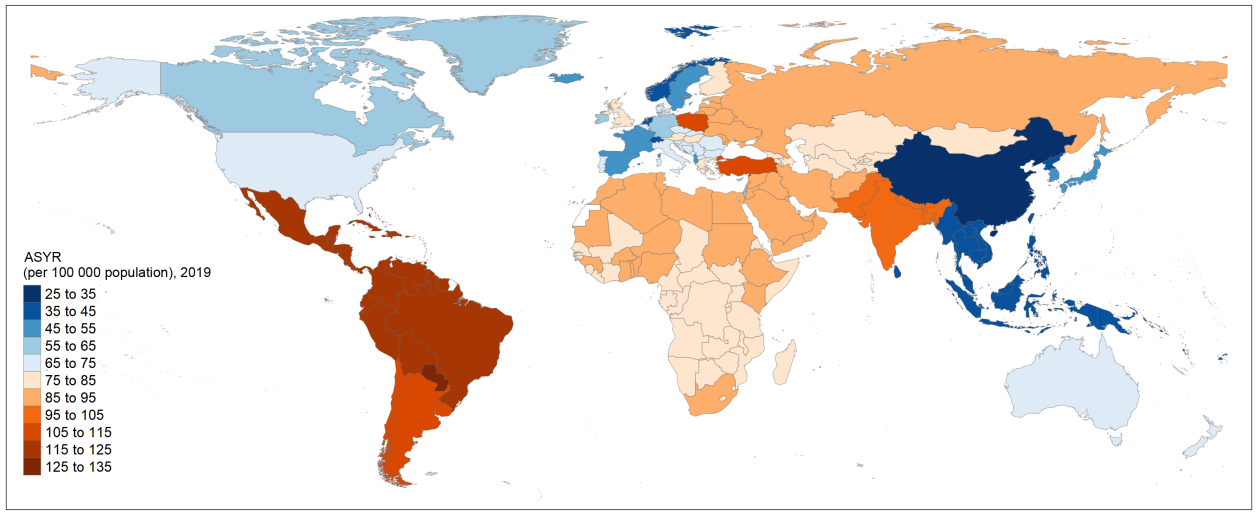


ASYR, age-standardized YLD rate; GERD, gastroesophageal reflux disease.

**Supplementary Figure 8.** The EAPC of ASIR for GERD in 204 countries and territories between 1990 and 2019.


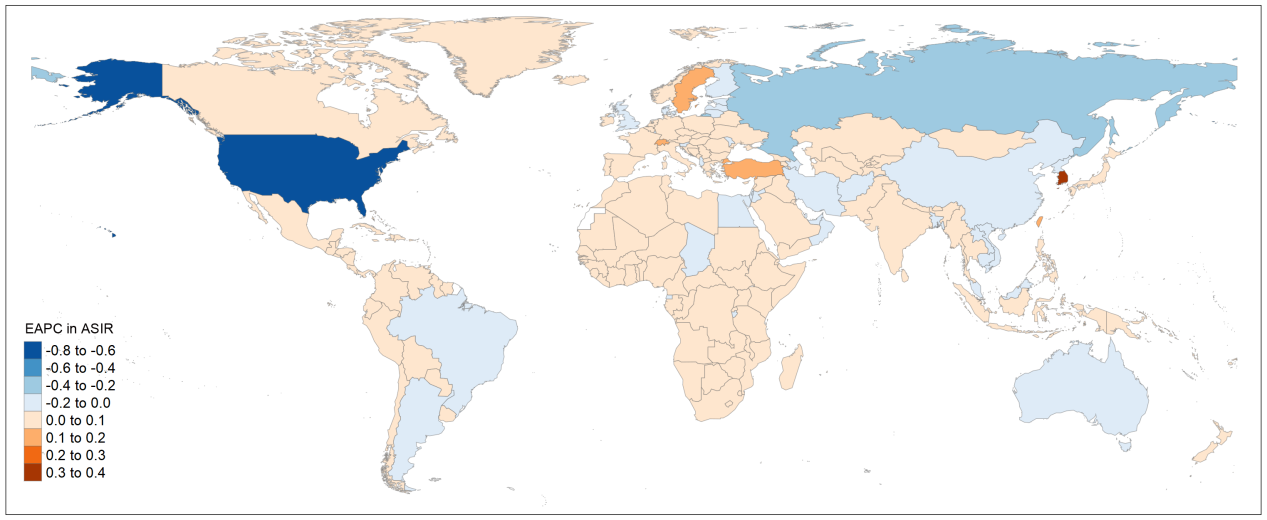


ASIR, age-standardised incidence rate; EAPC, estimated annual percentage change; GERD, gastroesophageal reflux disease.

**Supplementary Figure 9:** The EAPC of ASYR for GERD in 204 countries and territories between 1990 and 2019.

**
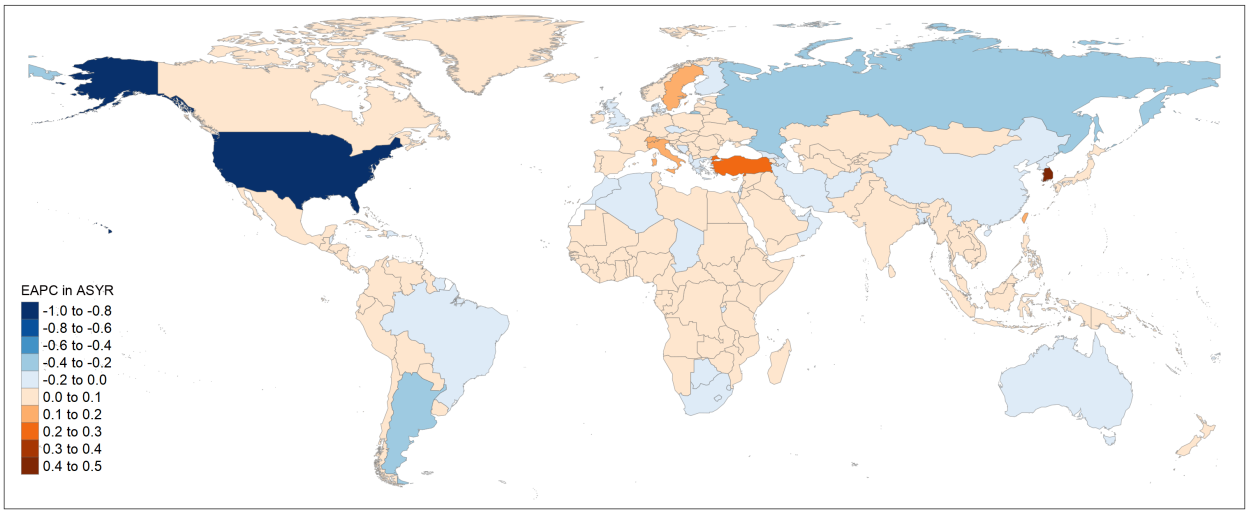
**

ASYR, age-standardized YLD rate; EAPC, estimated annual percentage change; GERD, gastroesophageal reflux disease.

**Supplementary Table 1** Gastroesophageal reflux disease included in GBD 2019

| ICD-10 codes | Conditions |
| --- | --- |
| K21 | Gastro-oesophageal reflux disease |
| K21.0 | Gastro-oesophageal reflux disease with oesophagitis  Reflux oesophagitis |
| K21.9 | Gastro-oesophageal reflux disease without oesophagitis  Oesophageal reflux NOS |
| K22.7 | Barrett's esophagus |
| R12 | Heartburn |

Abbreviations: GBD, Global Burden of Diseases, Injuries, and Risk Factors Study; ICD-10, 10th revision of the International Classification of Diseases; NOS, not otherwise specified.

**Supplementary Table 2** Prevalent cases, incident cases, and years of life lived with disability (YLDs) of gastroesophageal reflux disease (GERD) in 1990 and 2019 and their percentage change from 1990 to 2019

| Characteristics | Prevalent cases | | | | Incident cases | | | | YLDs | | |
| --- | --- | --- | --- | --- | --- | --- | --- | --- | --- | --- | --- |
|  | 1990 No. (95%UI) | 2019 No. (95%UI) | Percentage change（%） (95%UI) | 1990 No. (95%UI) | | 2019 No. (95%UI) | Percentage change（%） (95%UI) | 1990 No. (95%UI) | | 2019 No. (95%UI) | Percentage change（%） (95%UI) |
| Global | 441574363 (383480879 to 496840028) | 783945161 (689550733 to 876534233) | 77.53 (74.38 to 80.48) | 177004114 (154833551 to 201151188) | | 309381599 (272527195 to 349510437) | 74.79 (71.39 to 77.92) | 3402303 (1762371 to 6093058) | | 6028428 (3103988 to 10815634) | 77.19 (74.08 to 80.18) |
| Sex |  |  |  |  | |  |  |  | |  |  |
| Male | 209357326 (180883033 to 236527916) | 371299469 (325065564 to 416959488) | 77.35 (74.09 to 80.54) | 84369113 (73743127 to 96136377) | | 147263817 (129482109 to 166975925) | 74.55 (71.13 to 77.76) | 1622895 (839407 to 2902319) | | 2872510 (1479428 to 5183408) | 77.00 (73.74 to 80.24) |
| Female | 232217037 (202644666 to 261401141) | 412645692 (362642385 to 463196184) | 77.70 (74.33 to 80.68) | 92635001 (81195363 to 104991220) | | 162117782 (143288942 to 182238845) | 75.01 (71.44 to 78.21) | 1779409 (922964 to 3181335) | | 3155918 (1623853 to 5632673) | 77.36 (74.06 to 80.44) |
| SDI quintile |  |  |  |  | |  |  |  | |  |  |
| High | 80129582 (70492518 to 90046784) | 108963554 (96339328 to 123535247) | 35.98 (32.14 to 39.86) | 31618586 (27664986 to 35989332) | | 42820565 (37890499 to 49042946) | 35.43 (31.67 to 39.09) | 613882 (313803 to 1109140) | | 829714 (420693 to 1492912) | 35.16 (31.26 to 39.17) |
| High-middle | 104258233 (91355482 to 117108757) | 155329203 (137887005 to 173542022) | 48.99 (45.28 to 52.79) | 40934868 (35807097 to 46496531) | | 59657101 (52761656 to 67694442) | 45.45 (41.43 to 49.22) | 802970 (414860 to 1445578) | | 1193576 (610303 to 2157747) | 48.65 (44.92 to 52.41) |
| Middle | 116152841 (100500968 to 131537450) | 228622408 (199772197 to 257258463) | 96.83 (90.62 to 102.89) | 47288734 (41153433 to 53994580) | | 90256897 (79322383 to 102221182) | 86.91 (80.33 to 92.45) | 900248 (468334 to 1613342) | | 1763594 (907809 to 3164464) | 95.90 (89.76 to 101.93) |
| Low-middle | 98861524 (86013501 to 111479239) | 195611491 (171095393 to 219430480) | 97.86 (94.85 to 100.58) | 39928729 (34917892 to 45373810) | | 77640490 (68485515 to 87712250) | 92.54 (89.45 to 95.32) | 760700 (398400 to 1354386) | | 1504589 (784025 to 2678872) | 97.79 (94.87 to 100.51) |
| Low | 41902732 (36208673 to 47368554) | 94929363 (81954829 to 107279120) | 126.55 (125.43 to 127.72) | 17127223 (14930930 to 19502159) | | 38817852 (33856901 to 44260966) | 116.23 (115.22 to 117.23) | 322418 (169182 to 571645) | | 733185 (384712 to 1299601) | 127.40 (126.05 to 128.76) |
| GBD region |  |  |  |  | |  |  |  | |  |  |
| Andean Latin America | 4639874 (4055224 to 5225552) | 9934970 (8774283 to 11099233) | 114.12 (109.09 to 119.32) | 1807399 (1582992 to 2032179) | | 3753606 (3318993 to 4182895) | 107.68 (102.18 to 112.34) | 36034 (18786 to 63657) | | 76946 (39952 to 136341) | 113.54 (108.15 to 118.81) |
| Australasia | 1937387 (1693605 to 2201797) | 3130260 (2769824 to 3551039) | 61.57 (57.23 to 65.94) | 780400 (683949 to 898525) | | 1243583 (1099620 to 1422203) | 59.35 (54.79 to 64.06) | 14851 (7585 to 26922) | | 23867 (12122 to 43405) | 60.71 (55.82 to 65.70) |
| Caribbean | 4960970 (4365282 to 5556878) | 8001360 (7113300 to 8920132) | 61.29 (57.24 to 65.38) | 1904049 (1678441 to 2128661) | | 2986873 (2664282 to 3321929) | 56.87 (52.34 to 60.81) | 38417 (19999 to 68157) | | 61606 (32186 to 109087) | 60.36 (56.24 to 64.50) |
| Central Asia | 5980338 (5166428 to 6772666) | 9568453 (8202434 to 10836308) | 60.00 (56.52 to 63.52) | 2420746 (2108738 to 2753256) | | 3824162 (3325278 to 4381321) | 57.97 (54.28 to 61.47) | 46360 (23783 to 83401) | | 74174 (38068 to 133702) | 60.00 (56.11 to 63.74) |
| Central Europe | 14863126 (13071525 to 16779346) | 16918847 (15047428 to 19041022) | 13.83 (10.96 to 16.62) | 5781319 (5071870 to 6555032) | | 6438335 (5709327 to 7286614) | 11.36 (8.55 to 14.38) | 113974 (57965 to 204307) | | 129193 (65727 to 230366) | 13.35 (10.42 to 16.16) |
| Central Latin America | 19850194 (17256564 to 22247938) | 40724057 (36024577 to 45037586) | 105.16 (98.89 to 111.64) | 7837686 (6870971 to 8793414) | | 15486351 (13756037 to 17199541) | 97.59 (91.22 to 103.51) | 154007 (80562 to 272619) | | 314343 (163301 to 558984) | 104.11 (97.97 to 110.75) |
| Central Sub-Saharan Africa | 3906320 (3333613 to 4457041) | 9802005 (8357525 to 11178502) | 150.93 (148.70 to 153.16) | 1615620 (1403484 to 1865969) | | 4054442 (3514547 to 4687251) | 150.95 (148.94 to 153.36) | 30040 (15570 to 53247) | | 75814 (39443 to 133500) | 152.38 (148.59 to 156.00) |
| East Asia | 52254125 (44678263 to 60165723) | 84692834 (73460206 to 96979616) | 62.08 (53.22 to 70.86) | 21642039 (18659708 to 25044722) | | 33939051 (29518619 to 39643842) | 56.82 (47.19 to 65.36) | 406712 (207739 to 731861) | | 654695 (329593 to 1192670) | 60.97 (52.03 to 69.70) |
| Eastern Europe | 29286216 (25878125 to 32854853) | 31008844 (27494156 to 34804676) | 5.88 (3.80 to 7.91) | 11431851 (10080437 to 13020759) | | 11935372 (10564743 to 13560206) | 4.40 (2.16 to 6.47) | 223903 (113783 to 406443) | | 236818 (120535 to 428492) | 5.77 (3.61 to 7.85) |
| Eastern Sub-Saharan Africa | 13101295 (11224877 to 14858628) | 30799118 (26347324 to 35017174) | 135.08 (133.32 to 136.94) | 5444074 (4729652 to 6280442) | | 12795708 (11077327 to 14751190) | 135.04 (133.45 to 136.93) | 101115 (52718 to 178171) | | 238775 (124573 to 420008) | 136.14 (134.01 to 138.25) |
| High-income Asia Pacific | 12104351 (10586660 to 13847459) | 16884767 (14885589 to 19363357) | 39.49 (33.59 to 46.18) | 4987761 (4331624 to 5774219) | | 6773592 (5959336 to 7831094) | 35.80 (29.94 to 42.99) | 93512 (47357 to 169565) | | 129322 (65755 to 234499) | 38.30 (32.51 to 44.95) |
| High-income North America | 33730400 (29427491 to 38105207) | 43136676 (38017272 to 48933947) | 27.89 (21.78 to 34.40) | 12903826 (11185018 to 14660689) | | 16745460 (14814489 to 19114605) | 29.77 (23.94 to 35.68) | 257045 (132009 to 459535) | | 325592 (164304 to 579723) | 26.67 (20.55 to 33.40) |
| North Africa and Middle East | 30903227 (26652912 to 35130358) | 72251838 (63102947 to 80947469) | 133.80 (127.53 to 140.01) | 12468509 (10783617 to 14236294) | | 28325327 (24825600 to 32106727) | 127.17 (120.01 to 133.79) | 239878 (124957 to 431913) | | 559710 (289618 to 1006943) | 133.33 (126.92 to 139.68) |
| Oceania | 250166 (212303 to 289356) | 568997 (483274 to 659313) | 127.45 (124.38 to 130.57) | 105654 (90640 to 122176) | | 237822 (204406 to 275720) | 125.09 (121.87 to 128.46) | 1940 (993 to 3524) | | 4403 (2243 to 7993) | 126.95 (122.04 to 131.92) |
| South Asia | 109492566 (95107235 to 123796719) | 227493099 (198749134 to 256183186) | 107.77 (104.78 to 110.72) | 44134149 (38514110 to 50110470) | | 90165761 (79572298 to 102133256) | 104.30 (101.17 to 107.29) | 840433 (438714 to 1494106) | | 1744755 (909045 to 3124842) | 107.60 (104.58 to 110.55) |
| Southeast Asia | 20032216 (17125909 to 23042897) | 37884332 (32476039 to 43387628) | 89.12 (82.43 to 95.28) | 8369178 (7205877 to 9699036) | | 15456438 (13366180 to 17875431) | 84.68 (77.63 to 91.08) | 155379 (79473 to 278926) | | 293183 (148900 to 531697) | 88.69 (81.80 to 95.11) |
| Southern Latin America | 6488805 (5641310 to 7308025) | 10204386 (8924162 to 11472586) | 57.26 (55.24 to 59.09) | 2394344 (2105408 to 2723237) | | 3710048 (3275989 to 4214316) | 54.95 (52.81 to 56.86) | 50135 (25797 to 90201) | | 78553 (40647 to 140874) | 56.68 (54.19 to 59.07) |
| Southern Sub-Saharan Africa | 4412492 (3777350 to 5002431) | 8268701 (7098563 to 9350800) | 87.39 (83.82 to 90.68) | 1815866 (1581964 to 2082343) | | 3331659 (2900599 to 3812232) | 83.47 (79.13 to 87.16) | 34064 (17852 to 60250) | | 63445 (32819 to 113194) | 86.25 (82.64 to 89.69) |
| Tropical Latin America | 20853832 (18269389 to 23294497) | 40245627 (35511521 to 44588035) | 92.99 (85.67 to 100.81) | 8115960 (7151426 to 9051242) | | 15160898 (13456970 to 16783418) | 86.80 (80.31 to 93.18) | 161031 (84450 to 287389) | | 309932 (160236 to 553965) | 92.47 (84.97 to 100.46) |
| Western Europe | 38312284 (33873905 to 43118748) | 48043927 (42555669 to 54147516) | 25.40 (22.51 to 28.29) | 15182432 (13383136 to 17345731) | | 18779722 (16645762 to 21466481) | 23.69 (20.74 to 26.83) | 293589 (149481 to 534277) | | 366507 (186145 to 663160) | 24.84 (21.95 to 27.87) |
| Western Sub-Saharan Africa | 14214179 (12205423 to 16096956) | 34382064 (29505102 to 38936097) | 141.89 (139.70 to 143.66) | 5861254 (5103901 to 6730922) | | 14237389 (12381401 to 16433018) | 142.91 (141.14 to 144.57) | 109885 (57346 to 194256) | | 266797 (139635 to 471353) | 142.80 (140.39 to 144.79) |

Abbreviations: GBD, Global Burden of Disease; SDI, socio-demographic index; UI, uncertainty interval.

**Supplementary Table 3** EAPC of age-standardised prevalence, incidence, and YLD rates for gastroesophageal reflux disease (GERD) from 2015 to 2019

| Characteristics | EAPC of ASPR (95%CI) | EAPC of ASIR (95%CI) | EAPC of ASYR (95%CI) |
| --- | --- | --- | --- |
| Global | 0.56 (0.30 to 0.82) | 0.54 (0.30 to 0.79) | 0.56 (0.30 to 0.82) |
| Sex |  |  |  |
| Male | 0.52 (0.31 to 0.73) | 0.51 (0.30 to 0.73) | 0.52 (0.31 to 0.74) |
| Female | 0.60 (0.30 to 0.91) | 0.57 (0.30 to 0.84) | 0.60 (0.29 to 0.90) |
| SDI quintile |  |  |  |
| High | 0.43 (0.07 to 0.80) | 0.37 (0.08 to 0.67) | 0.40 (0.06 to 0.75) |
| High-middle | 0.75 (0.02 to 1.48) | 0.71 (0.04 to 1.38) | 0.75 (0.02 to 1.48) |
| Middle | 0.74 (0.32 to 1.17) | 0.72 (0.32 to 1.13) | 0.74 (0.32 to 1.16) |
| Low-middle | 0.17 (0.05 to 0.29) | 0.17 (0.06 to 0.27) | 0.18 (0.06 to 0.30) |
| Low | -0.08 (-0.12 to -0.03) | -0.05 (-0.08 to -0.03) | -0.07 (-0.12 to -0.03) |
| GBD region |  |  |  |
| Andean Latin America | 0.00 (0.00 to 0.00) | 0.00 (0.00 to 0.00) | -0.01 (-0.01 to 0.00) |
| Australasia | -0.01 (-0.01 to -0.01) | -0.01 (-0.01 to -0.01) | -0.04 (-0.06 to -0.02) |
| Caribbean | 0.00 (0.00 to 0.00) | 0.00 (0.00 to 0.00) | -0.02 (-0.02 to -0.01) |
| Central Asia | 0.00 (0.00 to 0.00) | 0.00 (0.00 to 0.00) | -0.01 (-0.02 to 0.00) |
| Central Europe | 0.01 (0.00 to 0.03) | 0.02 (0.02 to 0.02) | 0.00 (-0.02 to 0.02) |
| Central Latin America | 0.00 (0.00 to 0.00) | 0.00 (0.00 to 0.00) | -0.02 (-0.04 to 0.01) |
| Central Sub-Saharan Africa | 0.00 (0.00 to 0.00) | 0.00 (0.00 to 0.00) | 0.01 (0.00 to 0.02) |
| East Asia | 2.27 (-0.02 to 4.60) | 2.05 (-0.02 to 4.17) | 2.26 (-0.03 to 4.59) |
| Eastern Europe | 1.74 (0.02 to 3.48) | 1.41 (0.02 to 2.83) | 1.73 (0.02 to 3.46) |
| Eastern Sub-Saharan Africa | 0.00 (0.00 to 0.00) | 0.00 (0.00 to 0.00) | 0.01 (0.00 to 0.01) |
| High-income Asia Pacific | -0.76 (-1.78 to 0.27) | -0.55 (-1.29 to 0.20) | -0.75 (-1.76 to 0.27) |
| High-income North America | 1.22 (-0.20 to 2.66) | 0.98 (-0.17 to 2.14) | 1.15 (-0.24 to 2.57) |
| North Africa and Middle East | -0.07 (-0.11 to -0.02) | -0.03 (-0.05 to -0.01) | -0.07 (-0.12 to -0.02) |
| Oceania | 0.00 (0.00 to 0.00) | 0.00 (0.00 to 0.00) | -0.01 (-0.03 to 0.01) |
| South Asia | -0.03 (-0.07 to 0.00) | 0.00 (-0.01 to 0.00) | -0.02 (-0.05 to 0.00) |
| Southeast Asia | 0.00 (0.00 to 0.00) | 0.00 (0.00 to 0.00) | -0.01 (-0.01 to 0.00) |
| Southern Latin America | 0.00 (0.00 to 0.00) | 0.00 (0.00 to 0.00) | -0.02 (-0.03 to -0.01) |
| Southern Sub-Saharan Africa | -0.01 (-0.01 to -0.01) | -0.01 (-0.01 to -0.01) | -0.02 (-0.02 to -0.01) |
| Tropical Latin America | -0.03 (-0.05 to 0.00) | 0.02 (-0.01 to 0.04) | 0.00 (-0.01 to 0.01) |
| Western Europe | -0.12 (-0.23 to 0.00) | -0.08 (-0.16 to -0.01) | -0.13 (-0.24 to -0.02) |
| Western Sub-Saharan Africa | 0.01 (0.01 to 0.01) | 0.00 (0.00 to 0.00) | 0.02 (0.01 to 0.02) |

Abbreviations: ASIR, age-standardised incidence rate; ASPR, age-standardised prevalence rate; ASYR, age-standardised YLD rate; CI, confidence interval; EAPC, estimated annual percentage change; GBD, Global Burden of Disease; SDI, socio-demographic index; YLD, year of life lived with disability.

**Supplementary Table 4** Prevalent cases, incident cases, and YLDs for gastroesophageal reflux disease (GERD) by 204 countries or territories in 2019 and 2019, and their percentage change from 1990 to 2019

| Characteristics | Prevalent cases | | | | Incident cases | | | | YLDs | | |
| --- | --- | --- | --- | --- | --- | --- | --- | --- | --- | --- | --- |
|  | 1990 No. (95%UI) | 2019 No. (95%UI) | Percentage change（%） (95%UI) | 1990 No. (95%UI) | | 2019 No. (95%UI) | Percentage change（%） (95%UI) | 1990 No. (95%UI) | | 2019 No. (95%UI) | Percentage change（%） (95%UI) |
| Afghanistan | 978152 (846627 to 1108765) | 3044987 (2585243 to 3508217) | 211.30 (195.42 to 227.17) | 397567 (349403 to 452058) | | 1249710 (1066175 to 1441362) | 214.34 (200.24 to 228.40) | 7460 (3812 to 13462) | | 23476 (12178 to 42528) | 214.69 (197.80 to 230.99) |
| Albania | 182107 (156545 to 209399) | 222941 (194943 to 254334) | 22.42 (15.52 to 29.84) | 81624 (70557 to 94398) | | 97033 (85416 to 112503) | 18.88 (11.77 to 26.19) | 1412 (720 to 2539) | | 1714 (875 to 3131) | 21.35 (14.49 to 29.20) |
| Algeria | 2188852 (1887895 to 2502979) | 5092797 (4387057 to 5783724) | 132.67 (123.72 to 141.06) | 890749 (770356 to 1017267) | | 1995109 (1733398 to 2282525) | 123.98 (113.43 to 132.75) | 17040 (8842 to 30946) | | 39494 (20374 to 71252) | 131.77 (122.13 to 141.26) |
| American Samoa | 1933 (1640 to 2237) | 2755 (2363 to 3154) | 42.52 (35.72 to 49.37) | 814 (697 to 943) | | 1142 (994 to 1324) | 40.31 (34.02 to 47.24) | 15 (8 to 27) | | 21 (11 to 39) | 40.61 (33.55 to 48.32) |
| Andorra | 4872 (4216 to 5560) | 8894 (7772 to 10214) | 82.56 (72.82 to 92.12) | 1990 (1714 to 2287) | | 3558 (3096 to 4123) | 78.84 (69.07 to 88.52) | 38 (19 to 69) | | 68 (34 to 125) | 81.00 (70.80 to 91.09) |
| Angola | 729565 (620608 to 834601) | 2128463 (1815030 to 2429678) | 191.74 (190.16 to 193.50) | 302103 (261280 to 349279) | | 881387 (763978 to 1019730) | 191.75 (190.22 to 193.34) | 5641 (2897 to 9944) | | 16495 (8551 to 29267) | 192.42 (187.17 to 197.62) |
| Antigua and Barbuda | 8949 (7896 to 10005) | 16431 (14559 to 18342) | 83.61 (77.02 to 90.13) | 3407 (3006 to 3800) | | 6103 (5427 to 6784) | 79.15 (72.65 to 85.20) | 69 (36 to 124) | | 127 (66 to 224) | 82.97 (76.25 to 89.68) |
| Argentina | 4362874 (3801650 to 4913047) | 6729433 (5882781 to 7569746) | 54.24 (53.27 to 55.13) | 1605119 (1413897 to 1822782) | | 2453344 (2164558 to 2787569) | 52.84 (51.78 to 53.85) | 33697 (17313 to 60602) | | 51839 (26830 to 92995) | 53.84 (51.65 to 55.94) |
| Armenia | 334636 (287956 to 379276) | 382422 (334627 to 430981) | 14.28 (10.44 to 18.18) | 133997 (116683 to 153084) | | 149541 (131343 to 171178) | 11.60 (7.46 to 15.43) | 2590 (1337 to 4762) | | 2945 (1496 to 5386) | 13.72 (9.45 to 18.27) |
| Australia | 1582009 (1382163 to 1799361) | 2596210 (2293197 to 2952857) | 64.11 (60.01 to 68.30) | 639302 (559408 to 735788) | | 1035239 (915228 to 1185014) | 61.93 (57.55 to 66.64) | 12133 (6175 to 22050) | | 19799 (10095 to 35988) | 63.18 (58.36 to 68.23) |
| Austria | 970811 (842281 to 1108234) | 1227960 (1069511 to 1404636) | 26.49 (23.14 to 29.55) | 359473 (314928 to 411707) | | 448621 (393919 to 514289) | 24.80 (21.54 to 27.90) | 7434 (3797 to 13345) | | 9379 (4722 to 16829) | 26.16 (22.47 to 29.90) |
| Azerbaijan | 658629 (568646 to 747162) | 1181275 (1011639 to 1341384) | 79.35 (73.81 to 84.66) | 266600 (231450 to 303680) | | 468339 (407864 to 538737) | 75.67 (69.44 to 81.00) | 5117 (2601 to 9212) | | 9163 (4671 to 16796) | 79.06 (72.88 to 85.20) |
| Bahamas | 35957 (31280 to 40456) | 67272 (59395 to 75205) | 87.09 (79.17 to 94.96) | 13914 (12163 to 15698) | | 25126 (22252 to 28038) | 80.57 (73.06 to 87.74) | 280 (145 to 499) | | 520 (270 to 919) | 85.88 (78.20 to 94.11) |
| Bahrain | 53352 (45118 to 61984) | 206155 (175334 to 237296) | 286.41 (253.88 to 318.82) | 21255 (17877 to 24797) | | 79970 (67812 to 93652) | 276.24 (245.31 to 308.40) | 417 (213 to 752) | | 1600 (819 to 2889) | 283.84 (251.37 to 316.67) |
| Bangladesh | 9310245 (8008871 to 10538358) | 20040189 (17412520 to 22475494) | 115.25 (108.70 to 121.48) | 3771438 (3261934 to 4315596) | | 7847981 (6859982 to 8904964) | 108.09 (101.02 to 114.18) | 71930 (37146 to 126778) | | 154463 (79488 to 276852) | 114.74 (107.32 to 121.96) |
| Barbados | 42142 (37513 to 46924) | 59749 (53300 to 66464) | 41.78 (35.81 to 47.61) | 15827 (14049 to 17611) | | 21778 (19607 to 24007) | 37.60 (31.88 to 43.22) | 326 (170 to 577) | | 459 (237 to 817) | 40.83 (34.65 to 46.99) |
| Belarus | 1338427 (1174705 to 1509392) | 1403585 (1239014 to 1583513) | 4.87 (2.52 to 6.94) | 517681 (453556 to 587431) | | 535101 (470363 to 608705) | 3.37 (0.82 to 5.40) | 10270 (5226 to 18510) | | 10749 (5435 to 19226) | 4.66 (2.01 to 7.22) |
| Belgium | 1032506 (907548 to 1170680) | 1274335 (1116016 to 1438081) | 23.42 (18.30 to 29.20) | 406503 (356380 to 464843) | | 495004 (436757 to 567139) | 21.77 (17.58 to 25.80) | 7915 (3971 to 14295) | | 9707 (4937 to 17405) | 22.64 (17.44 to 28.67) |
| Belize | 20480 (17931 to 23071) | 59731 (52279 to 67095) | 191.65 (184.92 to 198.23) | 8054 (7039 to 9114) | | 22966 (20210 to 25691) | 185.16 (178.48 to 191.64) | 159 (83 to 283) | | 463 (240 to 818) | 190.67 (182.70 to 198.46) |
| Benin | 323399 (276734 to 368647) | 897863 (765421 to 1025116) | 177.63 (174.84 to 180.27) | 133529 (116026 to 153702) | | 372498 (322402 to 430817) | 178.96 (175.98 to 181.69) | 2499 (1286 to 4439) | | 6979 (3608 to 12326) | 179.24 (173.81 to 184.88) |
| Bermuda | 10843 (9562 to 12141) | 13866 (12397 to 15356) | 27.88 (21.54 to 34.48) | 4040 (3567 to 4508) | | 4985 (4484 to 5487) | 23.40 (17.45 to 29.42) | 84 (43 to 150) | | 107 (55 to 187) | 26.62 (19.90 to 33.30) |
| Bhutan | 53915 (46541 to 61211) | 95433 (83118 to 107738) | 77.01 (72.16 to 81.46) | 21942 (19035 to 25043) | | 37543 (32754 to 42689) | 71.10 (65.62 to 76.10) | 418 (216 to 749) | | 738 (381 to 1342) | 76.45 (70.81 to 81.67) |
| Bolivia (Plurinational State of) | 741996 (647874 to 835554) | 1728561 (1518463 to 1937429) | 132.96 (129.34 to 136.52) | 289092 (253290 to 324974) | | 658187 (581012 to 735276) | 127.67 (123.80 to 131.16) | 5746 (2993 to 10186) | | 13385 (6938 to 23713) | 132.93 (128.30 to 137.40) |
| Bosnia and Herzegovina | 454872 (395277 to 518756) | 431327 (379510 to 491600) | -5.18 (-9.50 to -0.48) | 183963 (159835 to 212494) | | 170007 (149661 to 195552) | -7.59 (-11.97 to -2.65) | 3515 (1785 to 6336) | | 3288 (1671 to 5921) | -6.44 (-11.00 to -1.41) |
| Botswana | 95093 (81262 to 108301) | 233656 (199664 to 267066) | 145.71 (139.27 to 152.15) | 39450 (34284 to 45393) | | 94590 (81487 to 109373) | 139.77 (132.19 to 147.08) | 735 (382 to 1297) | | 1795 (929 to 3195) | 144.2 (136.72 to 152.26) |
| Brazil | 20358919 (17834680 to 22739149) | 39154618 (34579542 to 43371836) | 92.32 (84.88 to 100.26) | 7925275 (6981949 to 8834619) | | 14749241 (13093179 to 16334074) | 86.10 (79.51 to 92.52) | 157188 (82468 to 280613) | | 301481 (155911 to 539032) | 91.80 (84.16 to 99.95) |
| Brunei Darussalam | 13083 (11156 to 15239) | 29630 (25344 to 33965) | 126.48 (115.84 to 137.33) | 5605 (4742 to 6559) | | 12435 (10676 to 14430) | 121.87 (111.55 to 132.04) | 102 (52 to 183) | | 230 (117 to 416) | 125.73 (114.65 to 137.69) |
| Bulgaria | 1013827 (888749 to 1162610) | 942872 (830445 to 1074157) | -7.00 (-9.18 to -4.74) | 403310 (355269 to 462941) | | 369130 (325800 to 423191) | -8.47 (-10.68 to -6.00) | 7775 (3953 to 13928) | | 7194 (3674 to 13109) | -7.48 (-9.92 to -5.04) |
| Burkina Faso | 652766 (559859 to 740502) | 1623120 (1386604 to 1851643) | 148.65 (144.71 to 152.41) | 268874 (234927 to 308938) | | 671154 (583276 to 773754) | 149.62 (146.08 to 152.92) | 5032 (2612 to 8933) | | 12618 (6537 to 22440) | 150.76 (144.98 to 155.94) |
| Burundi | 389631 (333156 to 444956) | 854153 (726951 to 978050) | 119.22 (116.98 to 121.50) | 160774 (139561 to 184925) | | 353899 (306082 to 409189) | 120.12 (117.98 to 122.40) | 3009 (1551 to 5325) | | 6607 (3431 to 11808) | 119.54 (115.04 to 123.40) |
| Cabo Verde | 26781 (23318 to 30243) | 59114 (50968 to 67038) | 120.73 (113.40 to 128.92) | 10952 (9616 to 12445) | | 23764 (20681 to 27336) | 116.98 (108.94 to 125.46) | 207 (108 to 371) | | 459 (236 to 815) | 121.07 (113.36 to 130.64) |
| Cambodia | 366906 (312494 to 424013) | 814612 (693725 to 938387) | 122.02 (116.86 to 127.05) | 155280 (133311 to 179597) | | 336998 (290966 to 387606) | 117.03 (110.85 to 122.19) | 2843 (1450 to 5126) | | 6311 (3209 to 11525) | 121.95 (115.36 to 129.77) |
| Cameroon | 737623 (631077 to 838267) | 2237147 (1901632 to 2563499) | 203.29 (199.41 to 206.82) | 304462 (264537 to 351052) | | 925636 (800082 to 1069165) | 204.02 (200.66 to 207.58) | 5695 (2971 to 10009) | | 17368 (8899 to 30611) | 205.00 (198.75 to 211.39) |
| Canada | 2229681 (1936950 to 2545917) | 3416950 (2990046 to 3916509) | 53.25 (47.10 to 59.57) | 927502 (801126 to 1071717) | | 1401982 (1227210 to 1617608) | 51.16 (44.81 to 57.25) | 17225 (8766 to 31297) | | 26176 (13360 to 47729) | 51.97 (45.79 to 58.95) |
| Central African Republic | 201437 (171727 to 229383) | 402918 (343980 to 459273) | 100.02 (97.79 to 102.26) | 83159 (72104 to 96197) | | 166700 (144420 to 192997) | 100.46 (98.33 to 102.78) | 1550 (797 to 2752) | | 3110 (1604 to 5479) | 100.71 (96.60 to 104.69) |
| Chad | 416033 (357504 to 471647) | 1027113 (875260 to 1170662) | 146.88 (143.02 to 150.32) | 171333 (149652 to 196630) | | 429179 (372304 to 495633) | 150.49 (146.53 to 154.07) | 3217 (1658 to 5708) | | 7969 (4130 to 14099) | 147.73 (142.19 to 153.11) |
| Chile | 1670660 (1444391 to 1896845) | 2921560 (2556272 to 3293475) | 74.87 (68.73 to 80.82) | 623454 (543614 to 711688) | | 1057246 (934447 to 1200729) | 69.58 (63.33 to 75.76) | 12930 (6642 to 23155) | | 22469 (11610 to 40351) | 73.77 (67.33 to 80.49) |
| China | 50338705 (43013935 to 57975682) | 81636483 (70733802 to 93513614) | 62.17 (53.20 to 70.92) | 20851665 (17977319 to 24138125) | | 32711266 (28441817 to 38211687) | 56.88 (47.16 to 65.40) | 391785 (200086 to 704710) | | 631038 (317697 to 1149229) | 61.07 (52.07 to 69.81) |
| Colombia | 4212461 (3666196 to 4749555) | 8253065 (7325038 to 9195712) | 95.92 (88.64 to 103.27) | 1636694 (1430804 to 1847223) | | 3083033 (2748258 to 3432735) | 88.37 (81.34 to 95.30) | 32725 (16996 to 58050) | | 63768 (33177 to 112914) | 94.86 (87.74 to 102.52) |
| Comoros | 33391 (28648 to 37791) | 68654 (59119 to 77875) | 105.60 (101.78 to 109.15) | 13806 (12071 to 15884) | | 27816 (24212 to 31877) | 101.48 (96.85 to 105.41) | 258 (134 to 455) | | 532 (275 to 943) | 105.99 (100.74 to 110.63) |
| Congo | 175609 (150018 to 199776) | 457783 (391717 to 521535) | 160.68 (153.55 to 168.24) | 72786 (63285 to 83777) | | 186710 (161374 to 215371) | 156.52 (149.41 to 164.18) | 1355 (703 to 2400) | | 3540 (1832 to 6291) | 161.26 (152.96 to 169.91) |
| Cook Islands | 838 (716 to 964) | 1083 (944 to 1242) | 29.34 (22.98 to 36.10) | 350 (303 to 404) | | 439 (386 to 511) | 25.62 (18.72 to 31.98) | 6 (3 to 12) | | 8 (4 to 15) | 27.39 (20.78 to 34.93) |
| Costa Rica | 392420 (342483 to 441348) | 829399 (734302 to 925504) | 111.35 (103.81 to 118.97) | 151882 (132913 to 171142) | | 309328 (275147 to 344444) | 103.66 (96.32 to 110.71) | 3050 (1593 to 5400) | | 6403 (3327 to 11406) | 109.92 (102.16 to 117.80) |
| Côte d'Ivoire | 835046 (708045 to 957935) | 2071077 (1764926 to 2371587) | 148.02 (144.01 to 151.82) | 347459 (298596 to 403287) | | 852732 (736093 to 985620) | 145.42 (141.50 to 149.20) | 6451 (3315 to 11450) | | 16076 (8363 to 28495) | 149.22 (143.33 to 155.20) |
| Croatia | 562359 (492088 to 641592) | 567321 (499525 to 644569) | 0.88 (-2.02 to 4.01) | 224031 (196623 to 256493) | | 222584 (196571 to 255293) | -0.65 (-3.49 to 2.70) | 4310 (2189 to 7786) | | 4311 (2176 to 7698) | 0.03 (-3.25 to 3.54) |
| Cuba | 1772407 (1565176 to 1977389) | 2317396 (2070997 to 2580857) | 30.75 (24.69 to 36.44) | 673405 (596681 to 751931) | | 843400 (760721 to 929595) | 25.24 (18.95 to 31.15) | 13710 (7151 to 24193) | | 17766 (9139 to 31501) | 29.58 (23.45 to 35.68) |
| Cyprus | 63998 (55600 to 73046) | 130886 (114300 to 149313) | 104.51 (100.79 to 107.85) | 26163 (22968 to 30047) | | 52608 (46063 to 60402) | 101.08 (96.49 to 104.67) | 494 (252 to 895) | | 1006 (511 to 1824) | 103.78 (98.84 to 108.72) |
| Czechia | 1154882 (1013442 to 1311581) | 1403776 (1237991 to 1602054) | 21.55 (18.56 to 24.74) | 461060 (404128 to 528264) | | 550878 (485893 to 631231) | 19.48 (16.38 to 22.89) | 8835 (4496 to 15871) | | 10651 (5454 to 19114) | 20.56 (17.14 to 24.24) |
| Democratic People's Republic of Korea | 917220 (787534 to 1048106) | 1439218 (1250478 to 1642833) | 56.91 (52.92 to 60.93) | 381612 (331691 to 444777) | | 588799 (515161 to 684949) | 54.29 (49.93 to 58.45) | 7149 (3649 to 12851) | | 11157 (5671 to 20118) | 56.07 (50.77 to 61.30) |
| Democratic Republic of the Congo | 2690875 (2297730 to 3068401) | 6543528 (5573266 to 7464052) | 143.17 (140.87 to 145.45) | 1112988 (966754 to 1284652) | | 2708808 (2345608 to 3131081) | 143.38 (141.22 to 145.67) | 20657 (10735 to 36656) | | 50587 (26381 to 88887) | 144.89 (140.20 to 149.41) |
| Denmark | 613268 (538463 to 691738) | 729084 (643928 to 823442) | 18.89 (13.52 to 24.05) | 231516 (203609 to 263387) | | 273256 (241058 to 310969) | 18.03 (13.78 to 22.56) | 4697 (2442 to 8390) | | 5569 (2866 to 9860) | 18.56 (13.23 to 24.00) |
| Djibouti | 32833 (27862 to 37819) | 110842 (94539 to 126831) | 237.59 (225.37 to 248.58) | 13786 (11821 to 15978) | | 45007 (38604 to 52107) | 226.48 (213.40 to 238.49) | 256 (132 to 456) | | 862 (447 to 1528) | 236.79 (223.75 to 249.15) |
| Dominica | 10427 (9281 to 11590) | 12419 (11028 to 13818) | 19.10 (15.56 to 22.55) | 3970 (3521 to 4402) | | 4602 (4137 to 5100) | 15.91 (12.17 to 19.12) | 81 (42 to 142) | | 95 (50 to 170) | 18.47 (14.84 to 22.17) |
| Dominican Republic | 881209 (768212 to 993643) | 1701171 (1498529 to 1901584) | 93.05 (87.79 to 98.33) | 344761 (300693 to 389489) | | 644003 (568215 to 719018) | 86.80 (81.03 to 91.83) | 6860 (3533 to 12226) | | 13175 (6841 to 23384) | 92.06 (86.06 to 97.91) |
| Ecuador | 1223469 (1067881 to 1378203) | 2710469 (2394241 to 3027719) | 121.54 (116.11 to 126.88) | 477047 (417654 to 537363) | | 1027438 (909347 to 1144778) | 115.37 (110.00 to 120.18) | 9513 (4955 to 16854) | | 20990 (10919 to 37301) | 120.64 (114.81 to 126.47) |
| Egypt | 5172188 (4438922 to 5885302) | 10658406 (9190474 to 12109702) | 106.07 (103.54 to 108.42) | 2082606 (1801115 to 2372991) | | 4241181 (3683121 to 4822568) | 103.65 (100.45 to 106.17) | 40190 (20917 to 72354) | | 82884 (42971 to 149220) | 106.23 (102.35 to 110.00) |
| El Salvador | 625360 (548868 to 702660) | 984765 (870430 to 1096207) | 57.47 (54.17 to 60.77) | 244144 (214692 to 274514) | | 372636 (329611 to 415272) | 52.63 (49.12 to 55.76) | 4837 (2516 to 8573) | | 7588 (3955 to 13347) | 56.87 (52.94 to 60.74) |
| Equatorial Guinea | 30450 (26049 to 34691) | 105541 (89647 to 121349) | 246.61 (234.78 to 259.41) | 12513 (10905 to 14376) | | 44232 (37997 to 51144) | 253.48 (242.11 to 265.27) | 233 (121 to 412) | | 819 (416 to 1462) | 250.96 (237.61 to 264.03) |
| Eritrea | 202930 (172607 to 232382) | 531009 (451726 to 608635) | 161.67 (159.77 to 163.54) | 84617 (72952 to 98077) | | 219852 (189949 to 254760) | 159.82 (157.66 to 162.06) | 1565 (814 to 2757) | | 4114 (2127 to 7276) | 162.86 (157.91 to 167.83) |
| Estonia | 204649 (180623 to 229992) | 198607 (175729 to 222860) | -2.95 (-5.24 to -0.62) | 79038 (69531 to 89544) | | 75480 (66721 to 85502) | -4.50 (-6.83 to -2.02) | 1567 (802 to 2796) | | 1517 (768 to 2705) | -3.24 (-5.72 to -0.59) |
| Eswatini | 53695 (45783 to 61173) | 99246 (84645 to 113489) | 84.83 (82.29 to 87.31) | 22413 (19420 to 25889) | | 40704 (35308 to 46915) | 81.60 (78.23 to 84.86) | 417 (215 to 738) | | 761 (391 to 1351) | 82.71 (78.86 to 86.46) |
| Ethiopia | 3588898 (3061322 to 4092094) | 8088339 (6885891 to 9231971) | 125.37 (122.95 to 127.57) | 1490958 (1291036 to 1715812) | | 3373821 (2914904 to 3909361) | 126.29 (124.11 to 128.41) | 27721 (14351 to 49035) | | 62805 (32744 to 110773) | 126.57 (123.53 to 129.51) |
| Fiji | 31562 (26731 to 36631) | 46754 (39875 to 53886) | 48.13 (42.71 to 53.78) | 13262 (11395 to 15367) | | 19238 (16674 to 22299) | 45.06 (39.59 to 50.47) | 245 (125 to 438) | | 360 (183 to 661) | 46.73 (40.46 to 53.92) |
| Finland | 644833 (568603 to 721895) | 772284 (683484 to 872232) | 19.76 (12.09 to 26.67) | 235340 (207173 to 269819) | | 279493 (247361 to 318166) | 18.76 (12.82 to 25.10) | 4938 (2533 to 8807) | | 5877 (3008 to 10475) | 19.02 (11.66 to 26.29) |
| France | 4645426 (4091688 to 5259571) | 5880314 (5191858 to 6660499) | 26.58 (23.02 to 30.14) | 1941879 (1707145 to 2229085) | | 2430552 (2148719 to 2803854) | 25.16 (21.53 to 29.09) | 35713 (18022 to 64339) | | 45017 (22699 to 81472) | 26.05 (21.93 to 30.53) |
| Gabon | 78384 (67549 to 88960) | 163772 (140668 to 185981) | 108.94 (105.21 to 113.11) | 32070 (28051 to 36793) | | 66604 (57847 to 76839) | 107.68 (104.11 to 112.02) | 604 (312 to 1075) | | 1264 (657 to 2228) | 109.17 (104.14 to 114.27) |
| Gambia | 67066 (57180 to 76765) | 171477 (146226 to 196138) | 155.68 (153.74 to 157.50) | 27904 (24020 to 32295) | | 71079 (61511 to 82050) | 154.73 (152.95 to 156.43) | 521 (266 to 921) | | 1330 (689 to 2360) | 155.48 (150.65 to 160.56) |
| Georgia | 622694 (541948 to 704087) | 480727 (424857 to 539930) | -22.80 (-24.65 to -20.94) | 246260 (216695 to 280605) | | 186920 (165393 to 212692) | -24.10 (-26.04 to -22.15) | 4812 (2450 to 8837) | | 3678 (1875 to 6664) | -23.56 (-25.70 to -21.36) |
| Germany | 7644790 (6716868 to 8590353) | 9040426 (7969692 to 10277731) | 18.26 (13.53 to 23.67) | 3108826 (2731580 to 3562449) | | 3612580 (3189649 to 4147672) | 16.20 (11.67 to 20.87) | 58616 (29805 to 107482) | | 68850 (34832 to 123722) | 17.46 (12.06 to 23.09) |
| Ghana | 1094077 (932309 to 1248141) | 2762878 (2361147 to 3151238) | 152.53 (150.53 to 154.35) | 451892 (392469 to 521534) | | 1131057 (981254 to 1305895) | 150.29 (148.08 to 152.44) | 8475 (4371 to 14966) | | 21452 (11103 to 38085) | 153.11 (148.69 to 157.65) |
| Greece | 1269759 (1112816 to 1444509) | 1459951 (1293135 to 1662356) | 14.98 (11.58 to 18.07) | 471176 (413508 to 540561) | | 530752 (467403 to 609231) | 12.64 (9.22 to 15.84) | 9770 (4980 to 17753) | | 11151 (5649 to 20105) | 14.14 (10.83 to 17.63) |
| Greenland | 4463 (3801 to 5155) | 5375 (4660 to 6161) | 20.44 (13.00 to 28.76) | 1833 (1567 to 2139) | | 2158 (1883 to 2514) | 17.74 (10.82 to 25.30) | 34 (18 to 62) | | 41 (21 to 74) | 19.71 (12.25 to 28.73) |
| Grenada | 11187 (9934 to 12455) | 18137 (16052 to 20243) | 62.13 (56.62 to 67.70) | 4280 (3794 to 4755) | | 6784 (6057 to 7579) | 58.50 (53.49 to 63.68) | 86 (45 to 153) | | 140 (72 to 249) | 61.82 (56.49 to 67.59) |
| Guam | 6409 (5423 to 7436) | 9441 (8171 to 10810) | 47.31 (39.54 to 55.71) | 2669 (2284 to 3107) | | 3854 (3371 to 4457) | 44.43 (36.42 to 52.83) | 50 (25 to 90) | | 73 (37 to 134) | 45.52 (37.23 to 54.29) |
| Guatemala | 860656 (750230 to 969572) | 2435067 (2130647 to 2737075) | 182.93 (180.51 to 185.20) | 336520 (295247 to 378855) | | 942153 (824625 to 1060183) | 179.97 (177.25 to 182.57) | 6655 (3470 to 11704) | | 18842 (9828 to 33547) | 183.15 (178.91 to 187.50) |
| Guinea | 466298 (402233 to 528299) | 917183 (782339 to 1045744) | 96.69 (93.56 to 99.81) | 190339 (166099 to 218177) | | 378880 (329587 to 436618) | 99.05 (96.22 to 102.16) | 3600 (1865 to 6362) | | 7115 (3687 to 12557) | 97.63 (93.04 to 102.19) |
| Guinea-Bissau | 69336 (59129 to 79094) | 143770 (122029 to 164966) | 107.35 (104.84 to 109.78) | 28752 (24972 to 33141) | | 59605 (51405 to 68942) | 107.30 (104.79 to 109.58) | 537 (277 to 951) | | 1116 (574 to 1980) | 108.06 (103.52 to 112.26) |
| Guyana | 95683 (83188 to 107917) | 120250 (106155 to 134597) | 25.68 (21.61 to 29.70) | 37488 (32652 to 42448) | | 45735 (40369 to 51158) | 22.00 (18.37 to 25.64) | 739 (383 to 1308) | | 925 (481 to 1632) | 25.08 (20.99 to 29.68) |
| Haiti | 743087 (649415 to 837534) | 1684267 (1466907 to 1900889) | 126.66 (124.77 to 128.58) | 288742 (253336 to 324421) | | 649367 (568794 to 728868) | 124.89 (122.58 to 127.19) | 5741 (2976 to 10123) | | 13009 (6738 to 23185) | 126.59 (123.25 to 130.41) |
| Honduras | 490162 (428110 to 552036) | 1329334 (1161770 to 1495399) | 171.20 (168.70 to 173.71) | 192848 (168980 to 217523) | | 514315 (450770 to 578060) | 166.69 (163.69 to 169.49) | 3806 (1963 to 6764) | | 10305 (5326 to 18341) | 170.76 (166.40 to 175.25) |
| Hungary | 1258732 (1090894 to 1439581) | 1353876 (1178155 to 1542521) | 7.56 (4.85 to 10.08) | 492329 (428166 to 566801) | | 522524 (457069 to 602159) | 6.13 (3.59 to 8.55) | 9619 (4919 to 17485) | | 10316 (5271 to 18560) | 7.25 (4.20 to 10.32) |
| Iceland | 16437 (14335 to 18643) | 26344 (23091 to 29796) | 60.27 (52.39 to 67.96) | 7088 (6172 to 8118) | | 11175 (9828 to 12874) | 57.66 (51.08 to 64.04) | 127 (65 to 230) | | 202 (103 to 369) | 59.56 (51.59 to 68.17) |
| India | 88274714 (76551427 to 99781642) | 181552690 (158485093 to 204380384) | 105.67 (102.5 to 108.84) | 35536943 (30952642 to 40366404) | | 71837990 (63576583 to 81351721) | 102.15 (99.03 to 105.30) | 676639 (352162 to 1202333) | | 1390218 (721788 to 2487108) | 105.46 (102.32 to 108.51) |
| Indonesia | 8099123 (6914979 to 9336116) | 14734723 (12647009 to 16908372) | 81.93 (75.49 to 87.74) | 3371805 (2893210 to 3927976) | | 5997915 (5171530 to 6942484) | 77.88 (71.60 to 83.96) | 62769 (32034 to 111618) | | 114033 (57835 to 203758) | 81.67 (75.25 to 87.95) |
| Iran (Islamic Republic of) | 4549645 (3892677 to 5195403) | 10406617 (8985571 to 11872520) | 128.73 (118.95 to 138.38) | 1898429 (1638735 to 2179475) | | 4155278 (3576245 to 4751335) | 118.88 (107.61 to 129.91) | 35296 (18023 to 63621) | | 80432 (40714 to 144806) | 127.88 (117.88 to 137.75) |
| Iraq | 1420264 (1224653 to 1620005) | 4397322 (3770700 to 5021402) | 209.61 (205.91 to 213.20) | 578636 (499104 to 660797) | | 1764352 (1519980 to 2018301) | 204.92 (200.58 to 209.23) | 10971 (5751 to 19735) | | 34057 (17665 to 61372) | 210.42 (204.12 to 216.46) |
| Ireland | 286310 (250034 to 326138) | 463291 (405400 to 531143) | 61.81 (57.63 to 65.63) | 117221 (103240 to 134378) | | 186446 (163643 to 214582) | 59.05 (54.56 to 62.79) | 2205 (1119 to 4016) | | 3556 (1796 to 6480) | 61.27 (56.43 to 65.80) |
| Israel | 393220 (344056 to 445484) | 812620 (714264 to 919013) | 106.66 (102.91 to 110.20) | 158389 (138876 to 181862) | | 323624 (284826 to 370484) | 104.32 (100.45 to 108.09) | 3039 (1544 to 5547) | | 6260 (3174 to 11351) | 105.98 (101.39 to 110.86) |
| Italy | 6870343 (6067825 to 7756648) | 8321445 (7377296 to 9400920) | 21.12 (17.63 to 24.39) | 2598062 (2291580 to 2964739) | | 3079086 (2721409 to 3514658) | 18.51 (14.69 to 22.24) | 52475 (26882 to 94918) | | 63320 (32654 to 113291) | 20.67 (17.09 to 24.05) |
| Jamaica | 312861 (276513 to 350526) | 484975 (429653 to 539946) | 55.01 (51.33 to 58.53) | 120682 (106562 to 135202) | | 182145 (161651 to 203072) | 50.93 (47.05 to 54.39) | 2428 (1265 to 4314) | | 3744 (1949 to 6628) | 54.21 (50.07 to 58.37) |
| Japan | 8998669 (7859770 to 10307199) | 11321847 (9986889 to 12925107) | 25.82 (19.91 to 32.47) | 3690932 (3206781 to 4288353) | | 4533287 (3998733 to 5233053) | 22.82 (16.93 to 29.94) | 69402 (34901 to 125940) | | 86439 (44316 to 155860) | 24.55 (18.94 to 31.26) |
| Jordan | 299423 (256312 to 344873) | 1240794 (1062180 to 1411378) | 314.40 (300.35 to 327.54) | 123630 (105986 to 142924) | | 495989 (428943 to 566632) | 301.19 (285.29 to 315.62) | 2336 (1205 to 4236) | | 9660 (5019 to 17570) | 313.51 (297.93 to 328.96) |
| Kazakhstan | 1568173 (1352647 to 1773785) | 1996549 (1720606 to 2257841) | 27.32 (24.66 to 29.78) | 629674 (548305 to 717262) | | 791115 (690496 to 906380) | 25.64 (22.72 to 28.08) | 12119 (6209 to 21768) | | 15405 (7881 to 27885) | 27.12 (23.83 to 30.17) |
| Kenya | 1546667 (1319381 to 1765633) | 4213624 (3586499 to 4819698) | 172.43 (168.33 to 176.58) | 648342 (560621 to 747463) | | 1743804 (1510519 to 2011711) | 168.96 (164.45 to 173.36) | 11981 (6245 to 21096) | | 32680 (16981 to 57889) | 172.77 (168.46 to 177.04) |
| Kiribati | 2974 (2530 to 3445) | 5255 (4452 to 6084) | 76.69 (73.81 to 79.72) | 1250 (1074 to 1447) | | 2191 (1884 to 2534) | 75.34 (72.16 to 78.15) | 23 (12 to 41) | | 41 (21 to 74) | 76.53 (71.66 to 81.63) |
| Kuwait | 184328 (156261 to 213093) | 608886 (515389 to 700088) | 230.33 (214.06 to 245.55) | 73588 (61923 to 85496) | | 236431 (199631 to 274571) | 221.29 (204.87 to 236.76) | 1443 (742 to 2582) | | 4740 (2447 to 8461) | 228.48 (212.17 to 244.4) |
| Kyrgyzstan | 376223 (325671 to 426567) | 620682 (533643 to 702666) | 64.98 (61.82 to 68.43) | 152368 (133036 to 173201) | | 249628 (217442 to 285126) | 63.83 (60.67 to 67.22) | 2915 (1502 to 5274) | | 4824 (2479 to 8693) | 65.51 (61.37 to 69.75) |
| Lao People's Democratic Republic | 154296 (131570 to 178176) | 334637 (283650 to 387037) | 116.88 (114.17 to 119.48) | 65102 (56225 to 75118) | | 139322 (120089 to 160954) | 114.01 (110.93 to 116.80) | 1197 (612 to 2180) | | 2602 (1327 to 4736) | 117.42 (111.72 to 123.33) |
| Latvia | 352372 (311190 to 396038) | 295333 (261977 to 331958) | -16.19 (-18.28 to -13.91) | 135941 (119559 to 154182) | | 112004 (99073 to 126980) | -17.61 (-19.81 to -15.30) | 2691 (1374 to 4829) | | 2250 (1149 to 3988) | -16.38 (-18.94 to -13.79) |
| Lebanon | 327011 (282025 to 370329) | 661104 (574679 to 749222) | 102.17 (97.65 to 106.52) | 130452 (114467 to 147902) | | 257052 (225137 to 291673) | 97.05 (91.63 to 102.88) | 2527 (1305 to 4592) | | 5089 (2624 to 9189) | 101.43 (96.05 to 106.87) |
| Lesotho | 141634 (121790 to 160477) | 198690 (170232 to 226408) | 40.28 (38.57 to 42.36) | 58178 (50866 to 66879) | | 80941 (70433 to 93193) | 39.13 (37.01 to 41.47) | 1094 (564 to 1936) | | 1523 (793 to 2685) | 39.27 (36.33 to 42.15) |
| Liberia | 148999 (128848 to 168428) | 389027 (332002 to 444524) | 161.09 (153.33 to 168.08) | 60738 (53254 to 69306) | | 160465 (138509 to 185277) | 164.19 (156.82 to 171.54) | 1139 (590 to 2022) | | 3000 (1537 to 5322) | 163.50 (154.44 to 171.46) |
| Libya | 346910 (299523 to 396970) | 873439 (748144 to 991226) | 151.78 (141.75 to 161.71) | 141726 (122036 to 162222) | | 343117 (297198 to 392855) | 142.10 (130.71 to 152.65) | 2700 (1406 to 4858) | | 6757 (3479 to 12059) | 150.25 (139.29 to 161.35) |
| Lithuania | 492512 (433970 to 555496) | 453805 (403393 to 506727) | -7.86 (-12.23 to -3.45) | 187743 (164947 to 212096) | | 169119 (149635 to 191206) | -9.92 (-13.46 to -6.03) | 3771 (1935 to 6792) | | 3458 (1763 to 6189) | -8.30 (-12.55 to -3.90) |
| Luxembourg | 36813 (32057 to 41879) | 62312 (54479 to 70887) | 69.27 (66.46 to 71.90) | 14839 (13017 to 16975) | | 25008 (21982 to 28708) | 68.53 (65.69 to 71.42) | 283 (143 to 514) | | 477 (241 to 865) | 68.85 (65.28 to 72.68) |
| Madagascar | 845024 (721388 to 963168) | 2074287 (1767628 to 2368197) | 145.47 (142.23 to 148.75) | 349683 (303885 to 402685) | | 857500 (742472 to 992722) | 145.22 (142.21 to 148.6) | 6534 (3377 to 11538) | | 16119 (8402 to 28544) | 146.71 (141.23 to 152.15) |
| Malawi | 668510 (570192 to 762215) | 1345606 (1148990 to 1539508) | 101.28 (99.57 to 103.05) | 277223 (240790 to 320083) | | 560933 (486558 to 646137) | 102.34 (100.57 to 104.09) | 5160 (2664 to 9085) | | 10429 (5403 to 18601) | 102.10 (98.39 to 105.72) |
| Malaysia | 740273 (628024 to 857190) | 1692761 (1446736 to 1950408) | 128.67 (123.20 to 134.57) | 310192 (266703 to 358371) | | 696235 (602374 to 803771) | 124.45 (118.06 to 130.32) | 5750 (2937 to 10414) | | 13119 (6671 to 24326) | 128.14 (121.20 to 136.20) |
| Maldives | 7461 (6370 to 8656) | 27472 (23112 to 32136) | 268.20 (249.20 to 288.09) | 3177 (2731 to 3674) | | 11237 (9543 to 13186) | 253.67 (232.73 to 277.26) | 58 (30 to 106) | | 215 (110 to 392) | 269.33 (248.64 to 292.25) |
| Mali | 622839 (534618 to 708639) | 1489345 (1270421 to 1692590) | 139.12 (135.45 to 142.73) | 255467 (222565 to 293822) | | 619731 (538014 to 715158) | 142.59 (139.08 to 146.75) | 4808 (2496 to 8497) | | 11571 (5955 to 20543) | 140.69 (135.28 to 146.14) |
| Malta | 32159 (28018 to 36631) | 46906 (41319 to 53315) | 45.86 (40.29 to 52.35) | 13056 (11451 to 15032) | | 18688 (16489 to 21510) | 43.15 (37.53 to 49.47) | 248 (126 to 451) | | 358 (182 to 657) | 44.55 (38.77 to 51.02) |
| Marshall Islands | 1489 (1259 to 1724) | 2645 (2239 to 3067) | 77.62 (71.38 to 83.94) | 637 (546 to 739) | | 1100 (947 to 1276) | 72.80 (66.22 to 78.72) | 12 (6 to 21) | | 20 (10 to 37) | 76.38 (69.42 to 83.84) |
| Mauritania | 152038 (130407 to 172225) | 323114 (276921 to 366378) | 112.52 (111.13 to 113.91) | 62541 (54523 to 71898) | | 132861 (115747 to 152983) | 112.44 (111.12 to 113.67) | 1179 (609 to 2090) | | 2511 (1300 to 4422) | 112.88 (109.32 to 116.61) |
| Mauritius | 53231 (45358 to 61436) | 83022 (72104 to 95468) | 55.97 (47.07 to 64.89) | 22115 (19018 to 25540) | | 33617 (29338 to 39033) | 52.01 (43.03 to 60.86) | 412 (211 to 745) | | 635 (323 to 1175) | 54.08 (45.05 to 63.71) |
| Mexico | 10199233 (8893565 to 11465454) | 20500311 (18098543 to 22748369) | 101.00 (94.51 to 107.43) | 4078356 (3576995 to 4585804) | | 7869414 (7006914 to 8724821) | 92.96 (86.18 to 99.06) | 79085 (41580 to 140200) | | 158059 (82573 to 284214) | 99.86 (93.42 to 106.37) |
| Micronesia (Federated States of) | 3701 (3142 to 4272) | 4853 (4131 to 5609) | 31.12 (26.74 to 35.67) | 1572 (1356 to 1817) | | 2022 (1744 to 2340) | 28.63 (24.56 to 32.99) | 29 (15 to 52) | | 38 (19 to 68) | 30.53 (25.42 to 35.93) |
| Monaco | 3394 (2996 to 3852) | 4213 (3702 to 4780) | 24.15 (21.44 to 26.97) | 1348 (1189 to 1547) | | 1670 (1464 to 1919) | 23.89 (21.09 to 26.73) | 26 (13 to 47) | | 32 (16 to 58) | 23.62 (20.42 to 27.19) |
| Mongolia | 157055 (135297 to 179028) | 343108 (293194 to 391954) | 118.46 (109.34 to 127.65) | 64839 (56026 to 74095) | | 136988 (118009 to 157526) | 111.27 (101.49 to 120.83) | 1221 (627 to 2188) | | 2665 (1369 to 4807) | 118.28 (108.03 to 128.24) |
| Montenegro | 62539 (54514 to 70891) | 75908 (66777 to 86670) | 21.38 (17.60 to 25.32) | 25214 (22066 to 28989) | | 30068 (26524 to 34426) | 19.25 (15.37 to 23.32) | 483 (247 to 874) | | 581 (296 to 1036) | 20.39 (16.31 to 24.80) |
| Morocco | 2386495 (2052323 to 2721385) | 4444727 (3839151 to 5036649) | 86.25 (80.10 to 92.04) | 962752 (831967 to 1098107) | | 1747470 (1532666 to 1992030) | 81.51 (74.89 to 87.01) | 18529 (9652 to 33565) | | 34361 (17572 to 62050) | 85.45 (79.05 to 91.82) |
| Mozambique | 949321 (815917 to 1081107) | 2027061 (1728156 to 2312895) | 113.53 (109.63 to 117.75) | 390246 (340116 to 448248) | | 844026 (731167 to 976212) | 116.28 (112.45 to 120.88) | 7300 (3794 to 12892) | | 15641 (8132 to 27858) | 114.26 (109.04 to 119.73) |
| Myanmar | 1699069 (1449333 to 1960647) | 2869044 (2451579 to 3302885) | 68.86 (63.81 to 73.75) | 713159 (615834 to 820881) | | 1181207 (1023001 to 1368057) | 65.63 (59.99 to 70.64) | 13166 (6714 to 23981) | | 22185 (11328 to 40506) | 68.50 (62.35 to 74.57) |
| Namibia | 107789 (92755 to 122096) | 218170 (187374 to 247602) | 102.40 (99.31 to 105.80) | 44492 (38787 to 51094) | | 88859 (77208 to 102231) | 99.72 (96.27 to 103.13) | 831 (430 to 1476) | | 1680 (864 to 2980) | 102.03 (97.69 to 106.3) |
| Nauru | 377 (317 to 438) | 431 (362 to 502) | 14.46 (13.63 to 15.25) | 159 (136 to 185) | | 182 (155 to 212) | 14.31 (13.55 to 15.00) | 3 (2 to 5) | | 3 (2 to 6) | 14.19 (11.24 to 17.24) |
| Nepal | 1781333 (1542793 to 2014179) | 3575087 (3117156 to 4014574) | 100.70 (98.19 to 103.45) | 713805 (623380 to 809516) | | 1412748 (1241840 to 1599591) | 97.92 (95.45 to 100.36) | 13719 (7068 to 24910) | | 27540 (14141 to 49676) | 100.75 (97.02 to 104.76) |
| Netherlands | 992421 (868031 to 1127366) | 1276904 (1125006 to 1449642) | 28.67 (24.12 to 33.27) | 432632 (379179 to 500706) | | 549899 (483871 to 635726) | 27.11 (22.32 to 31.75) | 7655 (3927 to 14067) | | 9781 (4973 to 17753) | 27.77 (22.61 to 33.00) |
| New Zealand | 355379 (309943 to 401001) | 534050 (469941 to 604760) | 50.28 (44.88 to 55.55) | 141098 (123932 to 161472) | | 208344 (184419 to 238710) | 47.66 (42.19 to 53.20) | 2718 (1384 to 4935) | | 4068 (2083 to 7385) | 49.68 (43.92 to 55.39) |
| Nicaragua | 395922 (344735 to 447504) | 950909 (832406 to 1068652) | 140.18 (134.67 to 145.76) | 156883 (136606 to 178010) | | 363976 (319935 to 407152) | 132.00 (125.49 to 137.52) | 3072 (1592 to 5472) | | 7372 (3823 to 13092) | 139.95 (133.87 to 146.63) |
| Niger | 517073 (441970 to 589948) | 1419705 (1208909 to 1616014) | 174.57 (171.35 to 177.66) | 214790 (186226 to 248118) | | 594567 (515900 to 684536) | 176.81 (173.41 to 180.39) | 4016 (2055 to 7066) | | 11049 (5713 to 19521) | 175.13 (169.77 to 181.15) |
| Nigeria | 7029802 (6012250 to 7996182) | 16326334 (13929150 to 18621977) | 132.24 (129.45 to 134.52) | 2896084 (2512109 to 3313451) | | 6775177 (5866886 to 7799951) | 133.94 (131.63 to 136.05) | 54345 (28184 to 96653) | | 126595 (65893 to 224416) | 132.95 (130.14 to 135.33) |
| Niue | 109 (95 to 125) | 98 (86 to 113) | -10.09 (-12.44 to -7.69) | 45 (40 to 52) | | 40 (35 to 46) | -11.34 (-13.79 to -9.01) | 1 (0 to 2) | | 1 (0 to 1) | -10.77 (-13.73 to -7.83) |
| North Macedonia | 197411 (172035 to 224549) | 267459 (234340 to 306870) | 35.48 (31.63 to 39.32) | 79810 (69651 to 91703) | | 106007 (93036 to 121694) | 32.82 (28.93 to 36.84) | 1524 (781 to 2736) | | 2052 (1045 to 3689) | 34.66 (30.70 to 39.11) |
| Northern Mariana Islands | 2175 (1816 to 2564) | 2658 (2292 to 3074) | 22.23 (8.90 to 37.00) | 906 (764 to 1067) | | 1084 (939 to 1274) | 19.61 (6.85 to 33.70) | 17 (9 to 31) | | 20 (10 to 38) | 20.11 (6.79 to 34.73) |
| Norway | 257260 (225075 to 293033) | 350474 (307033 to 401584) | 36.23 (30.85 to 41.84) | 107417 (94552 to 123186) | | 145146 (126829 to 169036) | 35.12 (30.65 to 40.08) | 1962 (1004 to 3541) | | 2674 (1361 to 4880) | 36.29 (30.91 to 41.74) |
| Oman | 168110 (143123 to 193622) | 564966 (475602 to 658378) | 236.07 (227.75 to 243.79) | 67727 (57688 to 78051) | | 222305 (186510 to 259373) | 228.24 (217.33 to 238.60) | 1313 (678 to 2345) | | 4423 (2240 to 7945) | 236.80 (226.12 to 246.55) |
| Pakistan | 10072358 (8762404 to 11352165) | 22229700 (19220139 to 25083163) | 120.70 (118.66 to 122.88) | 4090022 (3579789 to 4658440) | | 9029499 (7864038 to 10305955) | 120.77 (118.92 to 122.68) | 77727 (40635 to 138681) | | 171796 (90027 to 307018) | 121.03 (118.39 to 123.75) |
| Palau | 722 (617 to 835) | 1138 (979 to 1312) | 57.55 (47.11 to 67.75) | 301 (259 to 348) | | 461 (398 to 540) | 52.95 (42.47 to 63.84) | 6 (3 to 10) | | 9 (4 to 16) | 55.76 (44.78 to 67.02) |
| Palestine | 158339 (136330 to 181380) | 474374 (406618 to 543242) | 199.59 (194.44 to 205.14) | 64843 (55828 to 74426) | | 191464 (164821 to 219569) | 195.27 (189.93 to 201.03) | 1227 (632 to 2244) | | 3675 (1908 to 6624) | 199.54 (192.57 to 206.49) |
| Panama | 317071 (277243 to 356800) | 669729 (593093 to 746701) | 111.22 (105.21 to 117.10) | 122677 (107601 to 137774) | | 251492 (223803 to 279538) | 105.00 (99.02 to 110.61) | 2461 (1285 to 4380) | | 5171 (2694 to 9152) | 110.13 (103.92 to 116.72) |
| Papua New Guinea | 156466 (132833 to 181044) | 411534 (348779 to 477855) | 163.02 (159.78 to 166.50) | 66126 (56689 to 76606) | | 172425 (147893 to 200200) | 160.75 (157.39 to 164.53) | 1212 (622 to 2210) | | 3187 (1631 to 5774) | 162.96 (155.42 to 169.75) |
| Paraguay | 494912 (432457 to 555515) | 1091009 (959495 to 1219126) | 120.44 (117.30 to 123.48) | 190685 (166281 to 213559) | | 411657 (361675 to 459755) | 115.88 (112.37 to 118.78) | 3843 (1981 to 6788) | | 8451 (4339 to 14962) | 119.89 (115.88 to 124.20) |
| Peru | 2674409 (2339777 to 3010171) | 5495940 (4857033 to 6139347) | 105.50 (99.97 to 111.06) | 1041260 (912431 to 1171522) | | 2067980 (1829172 to 2304682) | 98.60 (92.60 to 103.72) | 20775 (10836 to 36747) | | 42571 (22096 to 75320) | 104.91 (98.69 to 110.93) |
| Philippines | 2562197 (2190022 to 2949889) | 5566045 (4783871 to 6392905) | 117.24 (112.03 to 122.46) | 1072234 (919288 to 1246867) | | 2287656 (1973969 to 2661871) | 113.35 (108.03 to 118.38) | 19861 (10203 to 35464) | | 43119 (21910 to 76839) | 117.10 (111.75 to 122.56) |
| Poland | 5685187 (4980700 to 6397514) | 7050652 (6252689 to 7903104) | 24.02 (20.90 to 27.20) | 2111330 (1867220 to 2368322) | | 2559565 (2276252 to 2857182) | 21.23 (18.18 to 24.36) | 43542 (22172 to 76935) | | 53924 (27443 to 95577) | 23.84 (20.73 to 27.07) |
| Portugal | 991759 (863156 to 1132812) | 1258885 (1105123 to 1439317) | 26.93 (22.89 to 30.44) | 392629 (343969 to 452034) | | 488094 (428889 to 563233) | 24.31 (20.24 to 28.14) | 7601 (3848 to 13616) | | 9600 (4864 to 17185) | 26.30 (22.13 to 30.54) |
| Puerto Rico | 576682 (512505 to 642830) | 729015 (654404 to 807228) | 26.42 (21.71 to 31.31) | 217001 (192779 to 241289) | | 264283 (238882 to 291186) | 21.79 (17.34 to 26.18) | 4452 (2322 to 7890) | | 5565 (2866 to 9898) | 25.01 (20.16 to 30.09) |
| Qatar | 49673 (41421 to 58066) | 390279 (326703 to 456039) | 685.69 (673.72 to 697.13) | 19658 (16339 to 23098) | | 152798 (127379 to 179775) | 677.27 (665.02 to 689.56) | 389 (198 to 705) | | 3055 (1553 to 5480) | 684.84 (665.84 to 705.33) |
| Republic of Korea | 2863967 (2457966 to 3276780) | 4982276 (4378194 to 5742303) | 73.96 (62.79 to 85.56) | 1197944 (1030385 to 1384711) | | 2009932 (1761911 to 2339295) | 67.78 (56.69 to 78.62) | 22227 (11306 to 39776) | | 38379 (19211 to 69923) | 72.67 (60.95 to 85.03) |
| Republic of Moldova | 514820 (450496 to 580760) | 542853 (478314 to 611340) | 5.45 (2.85 to 8.10) | 200876 (174731 to 228725) | | 207888 (182504 to 237043) | 3.49 (0.57 to 5.97) | 3959 (2020 to 7057) | | 4163 (2101 to 7445) | 5.16 (2.08 to 8.13) |
| Romania | 2489516 (2179576 to 2842911) | 2517417 (2224020 to 2870353) | 1.12 (-2.12 to 3.98) | 997821 (877348 to 1143041) | | 988930 (873475 to 1134323) | -0.89 (-4.11 to 2.30) | 19110 (9754 to 34543) | | 19246 (9834 to 34835) | 0.71 (-2.76 to 3.98) |
| Russian Federation | 19155806 (16836407 to 21559377) | 21217288 (18767607 to 23892031) | 10.76 (8.35 to 13.20) | 7499711 (6590086 to 8536505) | | 8185591 (7240578 to 9322319) | 9.15 (6.62 to 11.54) | 146386 (73979 to 266746) | | 161943 (81853 to 293186) | 10.63 (8.24 to 13.15) |
| Rwanda | 495256 (422675 to 566726) | 1051623 (897382 to 1202178) | 112.34 (109.69 to 115.16) | 205110 (177755 to 235826) | | 432381 (374730 to 498097) | 110.80 (108.02 to 113.71) | 3824 (1962 to 6795) | | 8138 (4207 to 14379) | 112.81 (108.03 to 117.45) |
| Saint Kitts and Nevis | 5728 (5074 to 6418) | 11013 (9730 to 12333) | 92.26 (82.52 to 101.70) | 2186 (1933 to 2438) | | 4083 (3637 to 4544) | 86.76 (78.36 to 95.13) | 44 (23 to 78) | | 85 (44 to 151) | 92.15 (82.24 to 101.50) |
| Saint Lucia | 17074 (15043 to 19208) | 32900 (29251 to 36665) | 92.68 (84.39 to 100.98) | 6633 (5840 to 7452) | | 12199 (10873 to 13554) | 83.91 (75.09 to 91.85) | 132 (69 to 233) | | 253 (131 to 448) | 91.50 (82.64 to 100.24) |
| Saint Vincent and the Grenadines | 13665 (12031 to 15387) | 20145 (17929 to 22444) | 47.42 (40.56 to 54.12) | 5315 (4679 to 5969) | | 7484 (6695 to 8309) | 40.80 (33.86 to 47.07) | 106 (55 to 188) | | 155 (80 to 275) | 46.28 (39.20 to 53.29) |
| Samoa | 6291 (5392 to 7234) | 9406 (8037 to 10816) | 49.52 (46.06 to 53.02) | 2672 (2317 to 3078) | | 3931 (3408 to 4523) | 47.14 (43.42 to 50.63) | 49 (25 to 89) | | 73 (37 to 134) | 48.84 (43.95 to 54.08) |
| San Marino | 2156 (1885 to 2447) | 3379 (2967 to 3840) | 56.75 (52.91 to 60.05) | 875 (771 to 1005) | | 1353 (1194 to 1554) | 54.65 (50.57 to 58.41) | 17 (8 to 30) | | 26 (13 to 47) | 55.75 (51.51 to 60.20) |
| Sao Tome and Principe | 8651 (7476 to 9784) | 17821 (15248 to 20288) | 105.98 (100.30 to 112.42) | 3566 (3122 to 4072) | | 7304 (6328 to 8421) | 104.85 (99.62 to 110.81) | 67 (35 to 119) | | 139 (72 to 244) | 106.62 (100.02 to 114.01) |
| Saudi Arabia | 1381200 (1180139 to 1591396) | 4648684 (3936029 to 5336535) | 236.57 (223.93 to 249.11) | 561628 (479012 to 646755) | | 1827651 (1551267 to 2115740) | 225.42 (211.17 to 239.01) | 10769 (5599 to 19122) | | 36198 (18614 to 64357) | 236.12 (222.33 to 250.74) |
| Senegal | 522698 (446635 to 593964) | 1208073 (1034100 to 1370437) | 131.12 (129.88 to 132.31) | 216426 (188634 to 249455) | | 497163 (432560 to 572675) | 129.71 (128.30 to 130.95) | 4046 (2100 to 7164) | | 9366 (4867 to 16521) | 131.51 (127.77 to 135.32) |
| Serbia | 1033791 (904330 to 1182398) | 1108016 (974707 to 1261503) | 7.18 (4.06 to 10.70) | 413040 (363111 to 475403) | | 437125 (385444 to 501626) | 5.83 (2.60 to 9.38) | 7957 (4030 to 14373) | | 8453 (4314 to 15354) | 6.24 (2.98 to 10.01) |
| Seychelles | 3401 (2931 to 3903) | 6109 (5245 to 7011) | 79.61 (71.52 to 87.88) | 1415 (1231 to 1621) | | 2481 (2153 to 2886) | 75.39 (66.58 to 83.75) | 26 (14 to 48) | | 47 (24 to 87) | 78.80 (69.81 to 88.00) |
| Sierra Leone | 282723 (242856 to 320504) | 646616 (550775 to 739573) | 128.71 (125.34 to 131.95) | 115661 (100724 to 132766) | | 267468 (231224 to 309014) | 131.25 (128.03 to 134.42) | 2182 (1126 to 3851) | | 5021 (2590 to 8916) | 130.10 (124.75 to 134.75) |
| Singapore | 228633 (196056 to 261105) | 551015 (478040 to 628107) | 141.00 (125.21 to 155.34) | 93280 (80159 to 108172) | | 217937 (190270 to 252257) | 133.64 (117.22 to 148.87) | 1780 (913 to 3242) | | 4273 (2170 to 7691) | 140.06 (123.37 to 155.72) |
| Slovakia | 547964 (480228 to 621885) | 699458 (615216 to 798470) | 27.65 (24.23 to 30.85) | 219913 (192133 to 251011) | | 275720 (242365 to 316051) | 25.38 (21.91 to 28.63) | 4207 (2143 to 7573) | | 5348 (2720 to 9623) | 27.13 (23.29 to 30.92) |
| Slovenia | 219940 (192675 to 249494) | 277823 (244981 to 316625) | 26.32 (22.10 to 30.58) | 87874 (77010 to 100707) | | 108764 (96259 to 125102) | 23.77 (19.53 to 28.32) | 1684 (859 to 3054) | | 2113 (1074 to 3769) | 25.45 (20.93 to 30.25) |
| Solomon Islands | 11534 (9826 to 13329) | 26269 (22210 to 30487) | 127.74 (123.03 to 132.83) | 4928 (4231 to 5701) | | 11049 (9488 to 12826) | 124.21 (119.19 to 129.81) | 90 (46 to 165) | | 204 (103 to 367) | 127.06 (119.44 to 134.42) |
| Somalia | 484208 (413428 to 555092) | 1367849 (1167496 to 1567540) | 182.49 (176.59 to 188.74) | 200637 (173906 to 231234) | | 572217 (494273 to 666424) | 185.20 (178.98 to 192.47) | 3742 (1932 to 6624) | | 10599 (5516 to 18746) | 183.23 (175.87 to 190.82) |
| South Africa | 3310762 (2833526 to 3768860) | 6308655 (5417195 to 7175995) | 90.55 (86.48 to 94.36) | 1357919 (1181025 to 1551168) | | 2528274 (2192497 to 2890894) | 86.19 (81.40 to 90.27) | 25535 (13362 to 45436) | | 48337 (24945 to 86613) | 89.29 (85.10 to 93.27) |
| South Sudan | 414230 (354262 to 473046) | 667916 (572209 to 757368) | 61.24 (56.76 to 65.72) | 172551 (149373 to 198782) | | 276952 (240922 to 317510) | 60.50 (56.09 to 65.06) | 3190 (1656 to 5626) | | 5143 (2661 to 9063) | 61.21 (56.07 to 66.48) |
| Spain | 3157132 (2766564 to 3594863) | 4413017 (3862538 to 4986140) | 39.78 (35.18 to 43.65) | 1314984 (1149711 to 1513710) | | 1804017 (1580863 to 2091042) | 37.19 (32.26 to 41.44) | 24273 (12419 to 44027) | | 33772 (17181 to 61275) | 39.13 (34.10 to 43.78) |
| Sri Lanka | 794530 (675493 to 918102) | 1284276 (1110152 to 1474311) | 61.64 (54.50 to 69.13) | 331066 (285437 to 382089) | | 523171 (456888 to 605609) | 58.03 (50.61 to 65.43) | 6167 (3167 to 11224) | | 9871 (5035 to 18133) | 60.06 (52.12 to 68.35) |
| Sudan | 1677724 (1442959 to 1914803) | 3794497 (3251923 to 4349137) | 126.17 (124.00 to 128.33) | 681916 (588056 to 777562) | | 1535322 (1321037 to 1759247) | 125.15 (122.97 to 127.28) | 13036 (6775 to 23334) | | 29515 (15376 to 53081) | 126.42 (122.45 to 130.41) |
| Suriname | 52570 (46123 to 59023) | 97038 (85929 to 108270) | 84.59 (78.43 to 90.48) | 20265 (17828 to 22755) | | 36227 (32285 to 40299) | 78.77 (72.16 to 84.54) | 407 (213 to 717) | | 746 (387 to 1319) | 83.15 (76.83 to 89.70) |
| Sweden | 717117 (629712 to 808841) | 951288 (834579 to 1085119) | 32.65 (27.33 to 38.22) | 295078 (258255 to 338251) | | 383784 (338496 to 442127) | 30.06 (25.83 to 35.03) | 5498 (2827 to 9949) | | 7274 (3747 to 13263) | 32.30 (26.70 to 38.28) |
| Switzerland | 396760 (346806 to 448095) | 550188 (480244 to 626731) | 38.67 (35.55 to 41.76) | 177392 (155824 to 204122) | | 243921 (215840 to 280254) | 37.50 (34.13 to 40.77) | 3034 (1556 to 5538) | | 4207 (2148 to 7672) | 38.66 (34.61 to 42.87) |
| Syrian Arab Republic | 999763 (859777 to 1146059) | 1686365 (1469093 to 1910510) | 68.68 (57.95 to 78.51) | 410050 (353044 to 471022) | | 671987 (590714 to 769532) | 63.88 (54.85 to 73.25) | 7790 (4001 to 13919) | | 13006 (6661 to 23437) | 66.97 (56.28 to 77.07) |
| Taiwan (Province of China) | 998200 (859896 to 1142721) | 1617133 (1414226 to 1837863) | 62.00 (50.09 to 75.21) | 408762 (349516 to 468897) | | 638986 (556558 to 743773) | 56.32 (45.40 to 68.29) | 7779 (4017 to 14229) | | 12500 (6304 to 22638) | 60.70 (48.31 to 74.23) |
| Tajikistan | 389180 (336209 to 442285) | 830225 (708336 to 949221) | 113.33 (108.45 to 118.55) | 159787 (138394 to 181888) | | 337420 (291706 to 386135) | 111.17 (106.25 to 116.66) | 3027 (1557 to 5454) | | 6466 (3305 to 11580) | 113.62 (107.47 to 119.61) |
| Thailand | 2703826 (2298730 to 3130808) | 4749586 (4130654 to 5458807) | 75.66 (65.20 to 86.48) | 1127675 (971262 to 1300487) | | 1913935 (1669877 to 2234065) | 69.72 (58.41 to 80.14) | 20996 (10652 to 38209) | | 36608 (18724 to 67472) | 74.35 (63.21 to 85.62) |
| Timor-Leste | 28905 (24403 to 33498) | 54793 (46878 to 63063) | 89.56 (83.45 to 96.44) | 12199 (10428 to 14204) | | 23122 (20071 to 26668) | 89.53 (83.34 to 96.84) | 225 (115 to 407) | | 424 (218 to 780) | 88.72 (81.65 to 96.85) |
| Togo | 240454 (204936 to 275226) | 650808 (555763 to 740884) | 170.66 (165.59 to 175.52) | 100289 (86743 to 115980) | | 266871 (230895 to 308074) | 166.10 (160.51 to 171.06) | 1866 (961 to 3346) | | 5057 (2616 to 8920) | 170.98 (164.27 to 177.88) |
| Tokelau | 70 (61 to 81) | 70 (60 to 80) | -1.24 (-3.47 to 1.10) | 29 (26 to 33) | | 29 (25 to 33) | -2.15 (-4.64 to 0.11) | 1 (0 to 1) | | 1 (0 to 1) | -1.44 (-4.48 to 1.68) |
| Tonga | 3753 (3210 to 4308) | 4665 (4019 to 5343) | 24.31 (21.78 to 26.57) | 1588 (1381 to 1832) | | 1938 (1689 to 2232) | 22.08 (19.39 to 24.63) | 29 (15 to 53) | | 36 (18 to 66) | 23.65 (19.93 to 27.40) |
| Trinidad and Tobago | 168436 (147307 to 189407) | 263953 (234481 to 294273) | 56.71 (50.17 to 63.17) | 64486 (56694 to 72221) | | 96969 (86430 to 107280) | 50.37 (43.94 to 56.43) | 1305 (680 to 2317) | | 2028 (1049 to 3596) | 55.37 (48.81 to 62.05) |
| Tunisia | 815443 (702535 to 928821) | 1565491 (1358400 to 1765225) | 91.98 (83.65 to 99.52) | 328208 (284737 to 374213) | | 608257 (533157 to 692315) | 85.33 (76.22 to 92.72) | 6347 (3283 to 11595) | | 12093 (6217 to 21913) | 90.53 (82.43 to 98.70) |
| Turkey | 6546387 (5651344 to 7426743) | 13227257 (12207283 to 14247243) | 102.05 (86.07 to 121.40) | 2545364 (2209759 to 2904147) | | 4851733 (4315371 to 5427680) | 90.61 (77.35 to 105.24) | 50790 (26301 to 91262) | | 102059 (53178 to 184472) | 100.94 (84.48 to 120.99) |
| Turkmenistan | 281053 (242078 to 320467) | 507939 (436190 to 573904) | 80.73 (74.26 to 86.76) | 115295 (99696 to 131668) | | 203336 (176842 to 233148) | 76.36 (69.65 to 82.7) | 2189 (1130 to 3930) | | 3946 (2025 to 7163) | 80.32 (73.32 to 87.33) |
| Tuvalu | 431 (368 to 496) | 597 (513 to 687) | 38.64 (37.01 to 40.24) | 178 (154 to 206) | | 247 (215 to 284) | 38.46 (36.83 to 40.08) | 3 (2 to 6) | | 5 (2 to 8) | 38.02 (34.63 to 41.66) |
| Uganda | 1129337 (965238 to 1286843) | 2764055 (2353038 to 3164567) | 144.75 (142.61 to 146.82) | 471063 (407602 to 541635) | | 1157109 (999038 to 1336883) | 145.64 (143.72 to 147.67) | 8685 (4530 to 15366) | | 21461 (11110 to 38038) | 147.09 (142.21 to 151.76) |
| Ukraine | 7227630 (6342238 to 8128793) | 6897374 (6069560 to 7755200) | -4.57 (-6.02 to -3.24) | 2810860 (2481853 to 3181436) | | 2650188 (2336870 to 2992678) | -5.72 (-7.41 to -4.33) | 55259 (28242 to 99698) | | 52738 (26966 to 94423) | -4.56 (-6.43 to -2.77) |
| United Arab Emirates | 197045 (164645 to 230135) | 1369742 (1125122 to 1610981) | 595.14 (539.07 to 648.38) | 78225 (65016 to 92178) | | 527547 (434567 to 629823) | 574.39 (518.84 to 626.06) | 1545 (781 to 2778) | | 10704 (5441 to 19417) | 592.74 (535.58 to 649.05) |
| United Kingdom | 7236932 (6373584 to 8141689) | 8936644 (7907755 to 10096884) | 23.49 (19.10 to 28.16) | 2755951 (2429780 to 3129803) | | 3375015 (2982218 to 3839513) | 22.46 (18.79 to 26.01) | 55288 (28283 to 100358) | | 68024 (34406 to 122609) | 23.04 (18.76 to 27.74) |
| United Republic of Tanzania | 1789203 (1527918 to 2034906) | 4259690 (3643902 to 4846923) | 138.08 (135.31 to 141.14) | 742470 (646128 to 853577) | | 1758855 (1528018 to 2030870) | 136.89 (134.25 to 140.02) | 13776 (7133 to 24288) | | 33001 (17013 to 58534) | 139.56 (134.54 to 144.43) |
| United States of America | 31495485 (27470837 to 35624833) | 39713666 (34961099 to 45039243) | 26.09 (19.83 to 32.81) | 11974196 (10383074 to 13594483) | | 15341054 (13550603 to 17501632) | 28.12 (22.21 to 34.12) | 239780 (123302 to 428321) | | 299369 (150725 to 531682) | 24.85 (18.67 to 31.67) |
| United States Virgin Islands | 16319 (14388 to 18270) | 20589 (18379 to 22877) | 26.16 (19.86 to 32.93) | 6164 (5448 to 6874) | | 7454 (6701 to 8225) | 20.93 (15.73 to 27.03) | 127 (65 to 224) | | 158 (81 to 280) | 24.52 (18.16 to 31.24) |
| Uruguay | 455008 (399487 to 510776) | 552881 (487816 to 619860) | 21.51 (20.12 to 22.68) | 165675 (146751 to 187252) | | 199272 (177141 to 224700) | 20.28 (18.91 to 21.55) | 3505 (1801 to 6299) | | 4242 (2195 to 7597) | 21.02 (19.17 to 22.93) |
| Uzbekistan | 1592696 (1373058 to 1814002) | 3225525 (2758812 to 3666699) | 102.52 (96.71 to 108.72) | 651926 (564691 to 743007) | | 1300876 (1127631 to 1494906) | 99.54 (93.60 to 105.82) | 12371 (6400 to 22352) | | 25080 (12805 to 44984) | 102.73 (95.55 to 109.92) |
| Vanuatu | 5489 (4655 to 6351) | 12471 (10631 to 14405) | 127.22 (124.11 to 130.53) | 2322 (1994 to 2683) | | 5218 (4499 to 6021) | 124.70 (121.19 to 127.83) | 43 (22 to 76) | | 96 (49 to 175) | 126.40 (120.25 to 132.33) |
| Venezuela (Bolivarian Republic of) | 2356908 (2052793 to 2657408) | 4771478 (4227119 to 5326240) | 102.45 (93.83 to 111.24) | 917683 (801637 to 1035016) | | 1780002 (1580068 to 1981665) | 93.97 (85.36 to 101.93) | 18317 (9488 to 32590) | | 36836 (19071 to 65023) | 101.10 (92.40 to 110.28) |
| Viet Nam | 2792372 (2394737 to 3213117) | 5617619 (4803811 to 6452749) | 101.18 (92.64 to 109.76) | 1172635 (1009734 to 1349463) | | 2289292 (1980485 to 2658065) | 95.23 (85.59 to 104.39) | 21701 (11183 to 39480) | | 43630 (22286 to 80462) | 101.05 (90.82 to 110.74) |
| Yemen | 982139 (844897 to 1123303) | 2821544 (2420964 to 3224125) | 187.29 (184.56 to 190.38) | 401112 (345640 to 459345) | | 1141827 (981907 to 1311933) | 184.67 (181.48 to 188.16) | 7602 (3894 to 13532) | | 21861 (11379 to 39147) | 187.58 (182.05 to 192.59) |
| Zambia | 522241 (444843 to 595374) | 1349787 (1145999 to 1548573) | 158.46 (154.99 to 162.42) | 218813 (189314 to 252479) | | 561305 (483920 to 649563) | 156.52 (152.60 to 160.91) | 4039 (2109 to 7111) | | 10452 (5355 to 18425) | 158.79 (152.69 to 164.66) |
| Zimbabwe | 703520 (600058 to 800725) | 1210284 (1033952 to 1381878) | 72.03 (69.48 to 74.81) | 293414 (254837 to 337281) | | 498291 (432239 to 574055) | 69.83 (66.79 to 72.72) | 5451 (2821 to 9651) | | 9349 (4822 to 16441) | 71.49 (67.62 to 75.34) |

Abbreviations: UI, uncertainty interval; YLD, years of life lived with disability.

**Supplementary Table 5** Age-standardised prevalence, incidence, and YLD rates for gastroesophageal reflux disease (GERD) by 204 countries or territories in 1990 and 2019, and their temporal trends from 1990 to 2019

| Characteristics | Age-standardised prevalence rate  per 100,000 population | | | | Age-standardized incidence rate  per 100,000 population | | | | Age-standardized YLD rate  per 100,000 population | | |
| --- | --- | --- | --- | --- | --- | --- | --- | --- | --- | --- | --- |
|  | 1990 No. (95%UI) | 2019 No. (95%UI) | EAPC No. (95%CI) | 1990 No. (95%UI) | | 2019 No. (95%UI) | EAPC No. (95%CI) | 1990 No. (95%UI) | | 2019 No. (95%UI) | EAPC No. (95%CI) |
| Afghanistan | 12188.41 (10593.17 to 13761.34) | 12136.52 (10560.51 to 13698.99) | -0.01 (-0.01 to -0.01) | 4777.45 (4210.21 to 5409.59) | | 4757.01 (4205.10 to 5398.90) | -0.01 (-0.01 to -0.01) | 92.58 (47.62 to 167.21) | | 92.21 (47.65 to 167.08) | -0.01 (-0.01 to 0.00) |
| Albania | 6560.35 (5673.20 to 7521.67) | 6557.53 (5673.70 to 7510.29) | -0.06 (-0.08 to -0.04) | 2891.45 (2530.97 to 3353.77) | | 2891.51 (2529.93 to 3355.75) | -0.04 (-0.05 to -0.03) | 50.58 (25.74 to 91.38) | | 50.67 (25.97 to 92.11) | -0.05 (-0.06 to -0.03) |
| Algeria | 12139.21 (10555.12 to 13701.23) | 12126.75 (10542.52 to 13691.37) | 0.00 (0.00 to 0.00) | 4759.17 (4202.90 to 5398.58) | | 4755.25 (4196.58 to 5393.53) | 0.00 (0.00 to 0.00) | 93.81 (48.57 to 169.81) | | 93.66 (48.63 to 169.01) | -0.01 (-0.01 to 0.00) |
| American Samoa | 5285.56 (4566.34 to 6052.05) | 5296.57 (4569.92 to 6057.75) | 0.01 (0.01 to 0.01) | 2165.30 (1892.29 to 2492.67) | | 2169.82 (1895.83 to 2500.76) | 0.01 (0.01 to 0.01) | 40.64 (20.77 to 74.11) | | 40.50 (20.66 to 74.20) | -0.01 (-0.01 to 0.00) |
| Andorra | 7762.63 (6746.70 to 8819.98) | 7779.31 (6763.08 to 8846.91) | 0.01 (0.01 to 0.01) | 3180.29 (2790.71 to 3641.68) | | 3186.49 (2799.20 to 3648.99) | 0.01 (0.01 to 0.01) | 59.98 (30.66 to 108.61) | | 60.04 (30.75 to 109.03) | 0.00 (0.00 to 0.01) |
| Angola | 11017.24 (9604.43 to 12434.98) | 11048.66 (9623.91 to 12465.06) | 0.01 (0.01 to 0.01) | 4382.31 (3847.70 to 5001.96) | | 4392.67 (3856.26 to 5013.40) | 0.01 (0.01 to 0.01) | 84.41 (43.70 to 150.10) | | 84.83 (44.07 to 151.86) | 0.02 (0.02 to 0.02) |
| Antigua and Barbuda | 15951.80 (14147.63 to 17785.43) | 15942.05 (14143.14 to 17778.62) | 0.00 (0.00 to 0.00) | 5980.20 (5314.28 to 6642.27) | | 5977.25 (5314.03 to 6640.36) | 0.00 (0.00 to 0.00) | 123.29 (64.13 to 218.12) | | 122.96 (63.84 to 217.33) | -0.01 (-0.01 to -0.01) |
| Argentina | 13626.98 (11861.15 to 15356.24) | 13626.28 (11861.68 to 15354.31) | -0.22 (-0.31 to -0.13) | 5002.85 (4406.29 to 5686.9) | | 5002.64 (4405.97 to 5687.14) | -0.14 (-0.19 to -0.08) | 105.24 (54.03 to 189.43) | | 105.14 (54.26 to 187.94) | -0.23 (-0.31 to -0.14) |
| Armenia | 10476.46 (9127.99 to 11794.68) | 10477.15 (9128.81 to 11792.08) | 0.00 (0.00 to 0.00) | 4170.03 (3660.47 to 4766.45) | | 4170.83 (3658.86 to 4767.61) | 0.00 (0.00 to 0.00) | 80.74 (41.46 to 147.27) | | 80.92 (41.33 to 147.23) | 0.01 (0.01 to 0.01) |
| Australia | 8508.90 (7412.50 to 9705.51) | 8509.09 (7408.69 to 9708.47) | -0.02 (-0.20 to 0.16) | 3453.92 (3015.85 to 3982.81) | | 3454.36 (3017.22 to 3984.12) | -0.01 (-0.14 to 0.11) | 65.32 (33.35 to 118.04) | | 65.36 (33.27 to 118.90) | -0.02 (-0.20 to 0.16) |
| Austria | 10222.09 (8754.50 to 11692.28) | 10190.20 (8720.43 to 11656.46) | 0.00 (-0.01 to 0.00) | 3843.76 (3362.55 to 4401.33) | | 3835.12 (3352.42 to 4392.28) | 0.00 (-0.01 to 0.00) | 78.64 (40.07 to 140.56) | | 78.52 (40.18 to 140.19) | 0.00 (0.00 to 0.00) |
| Azerbaijan | 10484.21 (9133.98 to 11804.15) | 10465.45 (9120.04 to 11781.43) | -0.01 (-0.01 to -0.01) | 4172.63 (3663.66 to 4769.28) | | 4166.36 (3657.56 to 4763.66) | -0.01 (-0.01 to 0.00) | 81.06 (41.53 to 146.43) | | 80.87 (41.37 to 146.52) | -0.01 (-0.01 to -0.01) |
| Bahamas | 15946.54 (14144.80 to 17781.53) | 15944.78 (14144.01 to 17781.05) | 0.00 (0.00 to 0.00) | 5978.16 (5312.62 to 6641.65) | | 5978.01 (5313.75 to 6641.46) | 0.00 (0.00 to 0.00) | 123.31 (63.79 to 219.46) | | 123.09 (64.04 to 217.48) | 0.00 (-0.01 to 0.00) |
| Bahrain | 11917.25 (10373.32 to 13462.44) | 11851.81 (10314.64 to 13403.39) | -0.03 (-0.04 to -0.03) | 4683.44 (4135.86 to 5318.43) | | 4663.17 (4108.43 to 5293.58) | -0.03 (-0.03 to -0.02) | 91.81 (47.14 to 166.35) | | 91.14 (46.92 to 164.49) | -0.04 (-0.04 to -0.03) |
| Bangladesh | 12946.88 (11362.69 to 14477.06) | 13044.64 (11445.08 to 14572.40) | -0.16 (-0.32 to -0.01) | 5022.84 (4434.42 to 5665.15) | | 5048.80 (4458.27 to 5688.97) | -0.10 (-0.20 to -0.01) | 99.10 (51.12 to 176.9) | | 100.18 (52.01 to 179.08) | -0.15 (-0.31 to 0.00) |
| Barbados | 15949.19 (14146.31 to 17782.58) | 15942.98 (14142.91 to 17779.32) | 0.00 (0.00 to 0.00) | 5978.13 (5311.18 to 6638.99) | | 5977.13 (5312.89 to 6640.46) | 0.00 (0.00 to 0.00) | 123.54 (64.11 to 219.19) | | 123.24 (64.16 to 218.35) | -0.01 (-0.01 to -0.01) |
| Belarus | 11321.18 (9933.92 to 12747.09) | 11302.83 (9920.30 to 12728.54) | 0.00 (0.00 to 0.00) | 4429.58 (3890.17 to 5038.63) | | 4424.32 (3886.63 to 5032.37) | 0.00 (0.00 to 0.00) | 87.06 (44.24 to 155.22) | | 87.13 (44.48 to 156.14) | 0.00 (0.00 to 0.01) |
| Belgium | 8410.58 (7343.03 to 9479.24) | 8528.38 (7365.25 to 9673.44) | 0.04 (0.02 to 0.06) | 3369.15 (2951.50 to 3843.31) | | 3401.30 (2983.24 to 3886.20) | 0.03 (0.02 to 0.04) | 64.78 (32.75 to 115.77) | | 65.60 (33.14 to 117.89) | 0.04 (0.02 to 0.06) |
| Belize | 15916.38 (14123.69 to 17761.24) | 15931.73 (14136.86 to 17779.71) | 0.00 (0.00 to 0.00) | 5971.16 (5313.77 to 6640) | | 5975.75 (5316.65 to 6643.92) | 0.00 (0.00 to 0.00) | 123.25 (64.18 to 219.00) | | 123.00 (63.83 to 218.39) | -0.01 (-0.01 to 0.00) |
| Benin | 11050.89 (9619.23 to 12472.70) | 11038.88 (9614.86 to 12451.26) | 0.00 (0.00 to 0.00) | 4393.83 (3856.02 to 5016.53) | | 4389.48 (3852.66 to 5009.00) | 0.00 (0.00 to 0.00) | 84.78 (43.88 to 151.48) | | 85.06 (43.87 to 152.23) | 0.01 (0.01 to 0.01) |
| Bermuda | 15941.80 (14140.9 to 17777.68) | 15936.30 (14135.30 to 17781.69) | 0.00 (0.00 to 0.00) | 5976.64 (5311.70 to 6642.42) | | 5976.10 (5313.10 to 6644.38) | 0.00 (0.00 to 0.00) | 123.50 (64.23 to 218.61) | | 123.42 (64.09 to 218.38) | 0.00 (0.00 to 0.00) |
| Bhutan | 13049.66 (11475.15 to 14583.36) | 13046.01 (11462.68 to 14582.06) | 0.00 (0.00 to 0.00) | 5054.86 (4472.93 to 5709.14) | | 5054.18 (4472.86 to 5708.38) | 0.00 (0.00 to 0.00) | 100.11 (51.90 to 180.94) | | 100.27 (51.81 to 182.11) | 0.01 (0.00 to 0.01) |
| Bolivia (Plurinational State of) | 15941.87 (14141.43 to 17783.11) | 15929.31 (14133.14 to 17775.30) | 0.00 (0.00 to 0.00) | 5977.76 (5314.49 to 6643.04) | | 5973.67 (5312.78 to 6642.31) | 0.00 (0.00 to 0.00) | 122.64 (63.52 to 216.77) | | 122.87 (63.88 to 218.07) | 0.01 (0.01 to 0.01) |
| Bosnia and Herzegovina | 9663.84 (8458.67 to 10945.14) | 9657.35 (8453.02 to 10944.31) | 0.00 (0.00 to 0.00) | 3896.78 (3409.43 to 4469.33) | | 3894.68 (3405.39 to 4468.33) | 0.00 (0.00 to 0.00) | 74.41 (37.80 to 133.77) | | 74.24 (37.97 to 133.88) | -0.01 (-0.01 to -0.01) |
| Botswana | 11055.76 (9633.76 to 12475.04) | 11043.57 (9630.31 to 12459.36) | 0.00 (0.00 to 0.00) | 4395.25 (3856.79 to 5017.00) | | 4390.92 (3856.22 to 5012.28) | 0.00 (0.00 to 0.00) | 84.75 (43.92 to 151.69) | | 84.10 (43.77 to 150.21) | -0.02 (-0.02 to -0.02) |
| Brazil | 16335.57 (14523.39 to 18037.91) | 16204.14 (14316.26 to 17944.68) | -0.12 (-0.16 to -0.08) | 6167.02 (5487.94 to 6809.26) | | 6146.60 (5457.64 to 6790.57) | -0.07 (-0.09 to -0.04) | 125.27 (65.32 to 221.84) | | 124.74 (64.60 to 222.06) | -0.11 (-0.15 to -0.07) |
| Brunei Darussalam | 6468.52 (5631.41 to 7333.68) | 6471.07 (5636.62 to 7337.59) | 0.00 (0.00 to 0.00) | 2700.79 (2356.13 to 3100.31) | | 2701.60 (2357.39 to 3103.17) | 0.00 (0.00 to 0.00) | 49.69 (25.42 to 89.61) | | 49.83 (25.58 to 90.21) | 0.01 (0.01 to 0.01) |
| Bulgaria | 9656.86 (8453.38 to 10943.90) | 9656.75 (8453.43 to 10939.53) | 0.00 (0.00 to 0.00) | 3894.65 (3405.19 to 4467.12) | | 3894.12 (3406.49 to 4468.09) | 0.00 (0.00 to 0.00) | 74.36 (38.01 to 134.18) | | 74.47 (38.12 to 134.13) | 0.00 (0.00 to 0.01) |
| Burkina Faso | 11057.07 (9622.1 to 12484.48) | 11052.35 (9622.64 to 12475.18) | 0.00 (0.00 to 0.00) | 4395.64 (3855.53 to 5016.89) | | 4393.83 (3857.17 to 5015.14) | 0.00 (0.00 to 0.00) | 84.68 (43.91 to 150.57) | | 85.21 (44.09 to 152.00) | 0.03 (0.02 to 0.03) |
| Burundi | 11045.51 (9615.63 to 12462.73) | 11012.57 (9604.32 to 12429.66) | -0.01 (-0.01 to -0.01) | 4391.63 (3856.94 to 5013.61) | | 4381.50 (3844.19 to 5002.99) | -0.01 (-0.01 to -0.01) | 84.65 (43.81 to 151.2) | | 84.44 (43.59 to 150.92) | -0.01 (-0.02 to 0.00) |
| Cabo Verde | 11077.44 (9620.24 to 12506.57) | 11025.04 (9618.39 to 12445.61) | -0.02 (-0.02 to -0.02) | 4401.36 (3863.79 to 5022.80) | | 4384.64 (3852.93 to 5004.27) | -0.01 (-0.01 to -0.01) | 85.65 (44.47 to 153.56) | | 85.18 (43.99 to 152.87) | -0.02 (-0.02 to -0.02) |
| Cambodia | 5325.72 (4599.15 to 6101.88) | 5316.33 (4591.17 to 6081.24) | -0.01 (-0.01 to -0.01) | 2181.54 (1904.56 to 2516.26) | | 2177.43 (1901.74 to 2509.55) | -0.01 (-0.01 to -0.01) | 40.87 (20.73 to 73.95) | | 40.92 (20.81 to 74.92) | 0.01 (0.01 to 0.01) |
| Cameroon | 11034.97 (9612.36 to 12447.98) | 11029.08 (9612.60 to 12443.81) | 0.00 (0.00 to 0.00) | 4388.58 (3851.46 to 5010.25) | | 4386.36 (3849.96 to 5007.13) | 0.00 (0.00 to 0.00) | 84.52 (43.83 to 150.37) | | 84.82 (43.83 to 151.72) | 0.02 (0.01 to 0.02) |
| Canada | 7165.07 (6232.20 to 8169.35) | 7162.60 (6229.36 to 8165.56) | 0.05 (0.03 to 0.07) | 3002.75 (2610.45 to 3488.08) | | 3002.14 (2611.14 to 3486.74) | 0.03 (0.02 to 0.05) | 55.39 (28.27 to 100.81) | | 55.30 (28.34 to 100.28) | 0.04 (0.02 to 0.06) |
| Central African Republic | 11042.97 (9618.54 to 12455.68) | 11044.91 (9636.99 to 12457.67) | 0.00 (0.00 to 0.00) | 4390.89 (3853.39 to 5011.74) | | 4391.56 (3854.00 to 5011.56) | 0.00 (0.00 to 0.00) | 84.18 (43.58 to 149.34) | | 84.43 (43.87 to 150.61) | 0.02 (0.01 to 0.02) |
| Chad | 11046.40 (9612.58 to 12462.35) | 11019.88 (9596.02 to 12431.93) | -0.01 (-0.01 to -0.01) | 4392.01 (3854.44 to 5013.78) | | 4383.51 (3844.65 to 5003.51) | -0.01 (-0.01 to -0.01) | 84.90 (43.97 to 152.46) | | 84.71 (43.81 to 151.35) | -0.01 (-0.01 to 0.00) |
| Chile | 13630.22 (11865.29 to 15360.69) | 13622.28 (11856.44 to 15349.85) | 0.00 (0.00 to 0.00) | 5003.91 (4407.65 to 5688.40) | | 5001.41 (4405.04 to 5685.64) | 0.00 (0.00 to 0.00) | 105.07 (54.36 to 188.71) | | 105.03 (53.95 to 187.75) | 0.00 (0.00 to 0.00) |
| China | 4532.19 (3927.36 to 5154.28) | 4509.32 (3899.11 to 5133.17) | -0.15 (-0.30 to 0.00) | 1849.31 (1612.82 to 2144.21) | | 1841.66 (1607.09 to 2133.51) | -0.14 (-0.27 to 0.00) | 35.04 (17.8 to 63.08) | | 34.94 (17.73 to 63.02) | -0.14 (-0.29 to 0.01) |
| Colombia | 15937.55 (14137.75 to 17783.77) | 15941.41 (14142.51 to 17777.35) | 0.00 (0.00 to 0.00) | 5977.37 (5315.90 to 6645.27) | | 5976.30 (5312.29 to 6639.16) | 0.00 (0.00 to 0.00) | 122.89 (63.86 to 217.98) | | 123.23 (63.88 to 218.22) | 0.01 (0.01 to 0.01) |
| Comoros | 11033.78 (9608.80 to 12446.19) | 11028.79 (9614.62 to 12446.71) | 0.00 (0.00 to 0.00) | 4387.63 (3851.52 to 5007.67) | | 4385.92 (3853.23 to 5005.87) | 0.00 (0.00 to 0.00) | 84.83 (43.91 to 151.42) | | 85.10 (44.13 to 152.40) | 0.01 (0.01 to 0.01) |
| Congo | 11045.56 (9622.78 to 12458.91) | 11027.97 (9618.88 to 12442.49) | -0.01 (-0.01 to -0.01) | 4391.35 (3858.00 to 5011.82) | | 4385.94 (3850.31 to 5005.80) | 0.00 (0.00 to 0.00) | 84.54 (43.80 to 150.63) | | 84.62 (43.94 to 150.79) | 0.01 (0.00 to 0.01) |
| Cook Islands | 5278.61 (4557.20 to 6042.16) | 5301.52 (4573.92 to 6064.68) | 0.02 (0.02 to 0.02) | 2162.68 (1889.76 to 2491.02) | | 2171.96 (1897.50 to 2505.54) | 0.02 (0.02 to 0.02) | 40.69 (20.72 to 74.41) | | 40.72 (20.95 to 74.13) | 0.00 (0.00 to 0.01) |
| Costa Rica | 15931.64 (14133.96 to 17778.12) | 15946.75 (14145.24 to 17783.44) | 0.00 (0.00 to 0.00) | 5974.99 (5314.22 to 6643.88) | | 5978.80 (5314.63 to 6641.03) | 0.00 (0.00 to 0.00) | 123.06 (64.05 to 217.74) | | 123.10 (64.09 to 218.52) | 0.00 (0.00 to 0.00) |
| Côte d'Ivoire | 10999.54 (9592.16 to 12420.09) | 11004.54 (9604.64 to 12423.99) | 0.00 (0.00 to 0.00) | 4377.04 (3841.62 to 4997.72) | | 4378.41 (3843.18 to 4998.84) | 0.00 (0.00 to 0.00) | 83.96 (43.49 to 148.69) | | 84.61 (44.10 to 151.08) | 0.02 (0.02 to 0.02) |
| Croatia | 9665.62 (8458.09 to 10953.33) | 9656.43 (8452.11 to 10941.22) | 0.00 (0.00 to 0.00) | 3897.53 (3410.62 to 4471.76) | | 3894.29 (3405.56 to 4467.68) | 0.00 (0.00 to 0.00) | 74.24 (38.01 to 133.45) | | 74.25 (37.85 to 133.59) | 0.00 (0.00 to 0.00) |
| Cuba | 15926.60 (14132.03 to 17772.93) | 15923.16 (14130.01 to 17769.23) | 0.00 (0.00 to 0.00) | 5973.10 (5313.38 to 6642.92) | | 5971.00 (5310.48 to 6636.25) | 0.00 (0.00 to 0.00) | 122.98 (64.00 to 216.73) | | 122.85 (64.23 to 217.61) | 0.00 (0.00 to 0.00) |
| Cyprus | 7805.36 (6777.77 to 8882.85) | 7808.77 (6782.82 to 8885.45) | 0.00 (0.00 to 0.00) | 3195.70 (2812.12 to 3658.88) | | 3197.07 (2813.20 to 3663.14) | 0.00 (0.00 to 0.00) | 60.23 (30.68 to 109.4) | | 60.23 (30.83 to 108.84) | 0.00 (0.00 to 0.00) |
| Czechia | 9663.85 (8455.47 to 10950.82) | 9653.03 (8450.87 to 10936.24) | 0.00 (0.00 to 0.00) | 3897.14 (3408.54 to 4471.57) | | 3892.82 (3404.95 to 4466.35) | 0.00 (0.00 to 0.00) | 74.21 (38.12 to 133.66) | | 74.09 (38.08 to 133.37) | -0.01 (-0.01 to -0.01) |
| Democratic People's Republic of Korea | 4698.82 (4092.61 to 5344.99) | 4672.09 (4069.89 to 5322.27) | -0.02 (-0.02 to -0.02) | 1942.59 (1705.23 to 2258.21) | | 1931.72 (1691.95 to 2244.82) | -0.02 (-0.02 to -0.02) | 36.44 (18.72 to 66.31) | | 36.24 (18.50 to 65.15) | -0.02 (-0.02 to -0.02) |
| Democratic Republic of the Congo | 11039.22 (9602.89 to 12456.72) | 11031.83 (9620.71 to 12448.72) | 0.00 (0.00 to 0.00) | 4389.12 (3856.92 to 5010.16) | | 4386.95 (3852.36 to 5006.26) | 0.00 (0.00 to 0.00) | 84.01 (43.67 to 148.73) | | 84.51 (43.89 to 150.49) | 0.02 (0.02 to 0.03) |
| Denmark | 9681.37 (8426.27 to 10927.77) | 9641.73 (8436.60 to 10918.63) | -0.03 (-0.03 to -0.02) | 3711.16 (3252.88 to 4227.67) | | 3702.28 (3249.03 to 4205.43) | -0.02 (-0.02 to -0.01) | 74.52 (38.68 to 132.31) | | 74.28 (38.37 to 132.30) | -0.02 (-0.03 to -0.02) |
| Djibouti | 10996.40 (9589.24 to 12419.69) | 10999.04 (9591.77 to 12416.35) | 0.00 (0.00 to 0.00) | 4374.64 (3843.11 to 4990.07) | | 4375.62 (3840.26 to 4991.62) | 0.00 (0.00 to 0.00) | 84.76 (43.83 to 151.35) | | 84.86 (43.81 to 150.70) | 0.01 (0.00 to 0.01) |
| Dominica | 15931.71 (14133.40 to 17773.42) | 15915.79 (14124.57 to 17761.57) | 0.00 (0.00 to 0.00) | 5971.21 (5304.35 to 6633.37) | | 5970.34 (5312.53 to 6639.54) | 0.00 (0.00 to 0.00) | 123.14 (64.10 to 217.91) | | 122.64 (63.77 to 217.36) | -0.01 (-0.01 to -0.01) |
| Dominican Republic | 15939.99 (14140.74 to 17785.38) | 15923.82 (14129.58 to 17772.58) | 0.00 (-0.01 to 0.00) | 5978.50 (5317.60 to 6645.49) | | 5972.25 (5312.45 to 6639.67) | 0.00 (-0.01 to 0.00) | 123.35 (64.22 to 219.08) | | 123.03 (63.96 to 217.58) | -0.01 (-0.01 to -0.01) |
| Ecuador | 15932.17 (14133.39 to 17779.76) | 15933.12 (14136.28 to 17776.29) | 0.00 (0.00 to 0.00) | 5975.69 (5315.28 to 6645.11) | | 5974.80 (5313.32 to 6641.85) | 0.00 (0.00 to 0.00) | 123.12 (63.70 to 217.22) | | 123.09 (63.94 to 217.87) | 0.00 (0.00 to 0.00) |
| Egypt | 12124.79 (10544.95 to 13688.56) | 12081.08 (10503.77 to 13639.71) | -0.01 (-0.01 to -0.01) | 4753.90 (4196.49 to 5393.19) | | 4740.61 (4181.78 to 5382.41) | -0.01 (-0.01 to -0.01) | 93.60 (48.26 to 169.07) | | 93.37 (48.28 to 168.40) | 0.00 (-0.01 to 0.00) |
| El Salvador | 15952.31 (14148.57 to 17789.31) | 15976.02 (14173.00 to 17791.38) | 0.01 (0.00 to 0.01) | 5981.26 (5316.61 to 6645.23) | | 5986.05 (5322.29 to 6649.26) | 0.00 (0.00 to 0.00) | 122.76 (64.14 to 217.09) | | 122.96 (63.99 to 217.11) | 0.01 (0.00 to 0.01) |
| Equatorial Guinea | 11068.09 (9633.56 to 12500.59) | 11034.80 (9604.67 to 12454.38) | -0.01 (-0.01 to -0.01) | 4398.96 (3859.95 to 5019.48) | | 4386.12 (3857.58 to 5002.70) | -0.01 (-0.01 to -0.01) | 84.15 (43.74 to 149.97) | | 84.60 (43.77 to 151.22) | 0.02 (0.02 to 0.03) |
| Eritrea | 11052.71 (9635.36 to 12468.57) | 11038.97 (9630.55 to 12456.54) | 0.00 (0.00 to 0.00) | 4393.35 (3858.34 to 5011.62) | | 4389.07 (3856.26 to 5008.39) | 0.00 (0.00 to 0.00) | 84.40 (43.93 to 151.17) | | 84.62 (43.68 to 151.77) | 0.01 (0.01 to 0.02) |
| Estonia | 11323.02 (9935.16 to 12749.19) | 11276.06 (9904.73 to 12699.60) | -0.01 (-0.02 to -0.01) | 4430.27 (3890.54 to 5039.42) | | 4415.54 (3879.77 to 5023.62) | -0.01 (-0.01 to -0.01) | 87.00 (44.44 to 155.06) | | 87.00 (44.46 to 155.83) | 0.00 (0.00 to 0.01) |
| Eswatini | 11063.77 (9644.37 to 12485.42) | 11059.06 (9648.11 to 12482.51) | 0.00 (0.00 to 0.00) | 4397.96 (3857.25 to 5018.74) | | 4395.94 (3860.01 to 5016.97) | 0.00 (0.00 to 0.00) | 84.98 (44.30 to 152.29) | | 84.01 (43.55 to 149.39) | -0.04 (-0.05 to -0.04) |
| Ethiopia | 11450.19 (9970.42 to 12968.41) | 11445.27 (9973.45 to 12962.82) | 0.00 (0.00 to 0.00) | 4556.79 (3993.35 to 5178.06) | | 4554.23 (3990.07 to 5174.75) | 0.00 (0.00 to 0.00) | 87.60 (45.17 to 156.48) | | 88.06 (45.23 to 158.29) | 0.02 (0.02 to 0.03) |
| Fiji | 5292.12 (4569.11 to 6054.07) | 5295.76 (4574.59 to 6058.34) | 0.00 (0.00 to 0.00) | 2168.09 (1894.39 to 2498.64) | | 2169.26 (1895.70 to 2497.82) | 0.00 (0.00 to 0.00) | 40.64 (20.68 to 73.77) | | 40.52 (20.47 to 74.46) | -0.01 (-0.01 to -0.01) |
| Finland | 10570.51 (9281.87 to 11839.26) | 10401.52 (9107.46 to 11688.82) | -0.09 (-0.10 to -0.07) | 3936.68 (3462.00 to 4501.06) | | 3884.74 (3410.25 to 4415.15) | -0.06 (-0.07 to -0.05) | 81.25 (42.02 to 144.48) | | 80.06 (41.03 to 143.9) | -0.08 (-0.10 to -0.07) |
| France | 6879.69 (6007.17 to 7816.23) | 6879.84 (6004.83 to 7814.37) | 0.08 (-0.15 to 0.32) | 2909.44 (2549.90 to 3344.82) | | 2909.69 (2550.36 to 3345.32) | 0.06 (-0.12 to 0.25) | 53.09 (26.72 to 95.82) | | 53.16 (26.92 to 95.68) | 0.09 (-0.15 to 0.33) |
| Gabon | 11029.90 (9617.82 to 12449.73) | 11039.80 (9619.37 to 12451.60) | 0.00 (0.00 to 0.00) | 4386.77 (3853.02 to 5009.59) | | 4390.16 (3849.71 to 5010.36) | 0.00 (0.00 to 0.00) | 84.55 (43.71 to 150.70) | | 84.65 (43.69 to 151.12) | 0.01 (0.00 to 0.01) |
| Gambia | 11010.77 (9613.30 to 12425.69) | 11030.89 (9613.00 to 12443.56) | 0.01 (0.01 to 0.01) | 4380.99 (3840.59 to 5001.27) | | 4387.00 (3849.22 to 5006.91) | 0.01 (0.01 to 0.01) | 84.72 (43.88 to 151.19) | | 84.84 (43.81 to 151.64) | 0.00 (0.00 to 0.01) |
| Georgia | 10490.19 (9140.46 to 11809.04) | 10474.87 (9129.37 to 11789.74) | 0.00 (0.00 to 0.00) | 4174.83 (3665.79 to 4770.08) | | 4169.41 (3658.71 to 4764.71) | 0.00 (0.00 to 0.00) | 81.09 (41.42 to 146.78) | | 80.73 (41.35 to 146.78) | -0.02 (-0.02 to -0.02) |
| Germany | 7528.11 (6579.44 to 8487.26) | 7658.63 (6681.36 to 8666.84) | 0.08 (0.05 to 0.10) | 3114.15 (2722.26 to 3583.68) | | 3150.72 (2751.50 to 3630.15) | 0.05 (0.04 to 0.07) | 57.99 (29.57 to 105.69) | | 58.97 (29.97 to 105.58) | 0.08 (0.05 to 0.10) |
| Ghana | 11034.13 (9617.42 to 12448.33) | 11051.34 (9626.52 to 12472.09) | 0.01 (0.01 to 0.01) | 4388.09 (3851.67 to 5009.31) | | 4393.45 (3857.03 to 5014.44) | 0.01 (0.00 to 0.01) | 84.80 (44.12 to 150.79) | | 85.17 (44.29 to 152.31) | 0.01 (0.01 to 0.02) |
| Greece | 10207.87 (8841.43 to 11640.07) | 10205.60 (8839.14 to 11639.59) | -0.01 (-0.02 to 0.01) | 3844.37 (3355.17 to 4415.12) | | 3843.86 (3354.83 to 4414.25) | 0.00 (-0.01 to 0.00) | 78.85 (40.39 to 143.81) | | 78.79 (40.50 to 144.24) | -0.01 (-0.02 to 0.00) |
| Greenland | 8124.04 (7100.21 to 9248.7) | 8121.54 (7091.66 to 9250.19) | 0.00 (0.00 to 0.00) | 3296.09 (2868.75 to 3815.29) | | 3297.26 (2873.28 to 3814.99) | 0.00 (0.00 to 0.00) | 62.10 (32.03 to 112.24) | | 62.32 (32.08 to 112.36) | 0.01 (0.01 to 0.02) |
| Grenada | 15939.68 (14140.83 to 17776.22) | 15916.93 (14124.84 to 17757.75) | 0.00 (0.00 to 0.00) | 5975.09 (5309.87 to 6639.01) | | 5969.69 (5309.95 to 6635.26) | 0.00 (0.00 to 0.00) | 123.09 (64.10 to 217.69) | | 122.67 (63.77 to 217.15) | -0.01 (-0.01 to -0.01) |
| Guam | 5277.00 (4556.53 to 6042.48) | 5286.68 (4567.31 to 6050.11) | 0.01 (0.00 to 0.01) | 2161.78 (1888.95 to 2488.22) | | 2165.74 (1892.42 to 2495.21) | 0.01 (0.00 to 0.01) | 40.80 (20.79 to 74.48) | | 40.79 (20.73 to 74.40) | 0.00 (-0.01 to 0.00) |
| Guatemala | 15936.80 (14136.98 to 17782.59) | 15959.20 (14157.98 to 17783.48) | 0.01 (0.00 to 0.01) | 5977.50 (5316.87 to 6645.78) | | 5981.35 (5314.81 to 6644.63) | 0.00 (0.00 to 0.00) | 122.33 (63.44 to 216.12) | | 122.65 (63.88 to 216.19) | 0.01 (0.01 to 0.01) |
| Guinea | 11040.60 (9613.14 to 12451.07) | 11041.64 (9608.98 to 12451.61) | 0.00 (0.00 to 0.00) | 4390.24 (3852.27 to 5011.14) | | 4390.69 (3850.78 to 5011.5) | 0.00 (0.00 to 0.00) | 84.83 (44.02 to 150.77) | | 85.02 (44.20 to 152.12) | 0.01 (0.00 to 0.01) |
| Guinea-Bissau | 11048.65 (9613.49 to 12467.02) | 11049.18 (9623.57 to 12464.54) | 0.00 (0.00 to 0.00) | 4392.75 (3854.20 to 5014.29) | | 4392.78 (3854.77 to 5013.07) | 0.00 (0.00 to 0.00) | 84.79 (43.97 to 151.51) | | 84.94 (44.25 to 151.80) | 0.01 (0.00 to 0.01) |
| Guyana | 15933.90 (14136.07 to 17780.14) | 15937.33 (14137.13 to 17778.31) | 0.00 (0.00 to 0.00) | 5975.78 (5314.59 to 6644.15) | | 5976.09 (5313.25 to 6642.57) | 0.00 (0.00 to 0.00) | 122.07 (63.58 to 215.75) | | 122.06 (63.61 to 216.09) | 0.00 (0.00 to 0.00) |
| Haiti | 15946.33 (14142.88 to 17788.35) | 15952.27 (14148.26 to 17787.19) | 0.00 (0.00 to 0.00) | 5980.70 (5317.76 to 6648.42) | | 5981.04 (5316.69 to 6643.20) | 0.00 (0.00 to 0.00) | 122.43 (63.68 to 217.06) | | 122.34 (63.92 to 216.22) | 0.00 (0.00 to 0.00) |
| Honduras | 15938.44 (14138.59 to 17784.58) | 15953.31 (14148.49 to 17787.84) | 0.00 (0.00 to 0.00) | 5977.76 (5316.50 to 6645.27) | | 5981.43 (5316.77 to 6644.18) | 0.00 (0.00 to 0.00) | 122.86 (63.60 to 217.03) | | 122.89 (63.78 to 217.20) | 0.00 (0.00 to 0.00) |
| Hungary | 10131.43 (8666.13 to 11585.90) | 10121.85 (8656.66 to 11575.39) | -0.01 (-0.01 to 0.00) | 4019.81 (3480.62 to 4620.88) | | 4016.48 (3476.96 to 4617.74) | 0.00 (0.00 to 0.00) | 77.78 (40 to 139.64) | | 77.94 (39.82 to 139.24) | 0.01 (0.01 to 0.01) |
| Iceland | 6169.85 (5393.98 to 7037.17) | 6251.15 (5423.64 to 7096.89) | 0.06 (0.04 to 0.07) | 2665.29 (2330.81 to 3072.60) | | 2692.82 (2350.37 to 3115.51) | 0.04 (0.03 to 0.06) | 47.63 (24.55 to 85.92) | | 48.26 (24.27 to 87.58) | 0.06 (0.05 to 0.07) |
| India | 13433.44 (11844.09 to 15077.69) | 13423.86 (11828.07 to 15065.83) | 0.02 (0.00 to 0.03) | 5247.11 (4653.88 to 5917.17) | | 5245.75 (4654.39 to 5915.26) | 0.01 (0.00 to 0.02) | 101.88 (52.89 to 183.89) | | 102.26 (53.10 to 184.31) | 0.04 (0.02 to 0.05) |
| Indonesia | 5551.77 (4827.87 to 6310.81) | 5548.24 (4827.47 to 6305.82) | 0.00 (0.00 to 0.00) | 2250.39 (1955.33 to 2592.90) | | 2248.88 (1953.53 to 2591.93) | 0.00 (0.00 to 0.00) | 42.58 (21.63 to 76.22) | | 42.69 (21.62 to 76.51) | 0.01 (0.01 to 0.01) |
| Iran (Islamic Republic of) | 11409.38 (9947.09 to 12910.68) | 11433.69 (9963.60 to 12938.89) | -0.07 (-0.16 to 0.01) | 4582.96 (4025.77 to 5215.86) | | 4590.85 (4033.41 to 5224.17) | -0.06 (-0.13 to 0.01) | 87.70 (44.48 to 157.73) | | 88.01 (44.53 to 158.39) | -0.07 (-0.16 to 0.02) |
| Iraq | 12127.16 (10550.11 to 13691.22) | 12124.16 (10547.23 to 13687.46) | 0.00 (0.00 to 0.00) | 4754.45 (4198.76 to 5395.65) | | 4753.34 (4197.26 to 5393.37) | 0.00 (0.00 to 0.00) | 92.84 (47.95 to 168.08) | | 93.10 (48.02 to 168.51) | 0.01 (0.01 to 0.01) |
| Ireland | 7804.17 (6782.38 to 8884.43) | 7805.28 (6782.21 to 8882.70) | 0.00 (0.00 to 0.00) | 3195.08 (2811.44 to 3657.24) | | 3195.88 (2812.52 to 3659.72) | 0.00 (0.00 to 0.00) | 60.21 (30.59 to 109.16) | | 60.19 (30.52 to 109.23) | 0.00 (0.00 to 0.00) |
| Israel | 8372.60 (7314.88 to 9477.53) | 8364.97 (7307.88 to 9465.69) | -0.05 (-0.09 to 0.00) | 3358.36 (2943.72 to 3856.39) | | 3355.05 (2940.53 to 3851.54) | -0.04 (-0.07 to -0.01) | 64.72 (32.72 to 117.74) | | 64.63 (32.81 to 116.78) | -0.05 (-0.09 to 0.00) |
| Italy | 9698.72 (8496.60 to 10954.12) | 9697.97 (8496.99 to 10948.32) | 0.08 (0.04 to 0.12) | 3725.76 (3271.33 to 4256.14) | | 3724.50 (3270.21 to 4254.95) | 0.07 (0.04 to 0.10) | 74.46 (38.18 to 133.68) | | 74.71 (38.33 to 134.16) | 0.10 (0.06 to 0.14) |
| Jamaica | 15940.27 (14138.32 to 17783.82) | 15933.59 (14135.39 to 17776.72) | 0.00 (0.00 to 0.00) | 5977.65 (5314.63 to 6644.47) | | 5975.47 (5314.32 to 6642.86) | 0.00 (0.00 to 0.00) | 123.42 (64.19 to 218.67) | | 122.96 (64.16 to 217.35) | -0.01 (-0.01 to -0.01) |
| Japan | 5892.17 (5129.80 to 6733.56) | 5920.25 (5159.24 to 6767.49) | 0.07 (0.01 to 0.12) | 2441.38 (2130.24 to 2820.14) | | 2452.04 (2139.86 to 2831.52) | 0.07 (0.02 to 0.12) | 45.55 (23.19 to 82.70) | | 45.89 (23.33 to 83.33) | 0.07 (0.02 to 0.13) |
| Jordan | 12087.21 (10504.98 to 13652.26) | 12044.92 (10471.57 to 13608.46) | -0.01 (-0.02 to -0.01) | 4740.38 (4184.75 to 5379.83) | | 4726.49 (4173.73 to 5363.62) | -0.01 (-0.01 to 0.00) | 93.33 (48.18 to 168.63) | | 93.09 (48.03 to 168.08) | 0.00 (-0.01 to 0.00) |
| Kazakhstan | 10493.68 (9148.86 to 11812.64) | 10482.68 (9134.88 to 11800.97) | 0.00 (0.00 to 0.00) | 4175.54 (3665.95 to 4766.95) | | 4172.31 (3662.41 to 4767.22) | 0.00 (0.00 to 0.00) | 80.75 (41.46 to 146.42) | | 80.70 (41.32 to 145.69) | 0.00 (0.00 to 0.00) |
| Kenya | 11451.50 (9978.57 to 12973) | 11455.98 (9978.82 to 12971.11) | 0.00 (0.00 to 0.00) | 4556.62 (3993.72 to 5179.80) | | 4558.03 (3995.29 to 5183.8) | 0.00 (0.00 to 0.00) | 87.83 (45.08 to 157.47) | | 88.02 (45.12 to 158.02) | 0.02 (0.01 to 0.02) |
| Kiribati | 5310.27 (4584.51 to 6073.18) | 5317.25 (4592.24 to 6083.38) | 0.00 (0.00 to 0.01) | 2174.98 (1900.19 to 2506.01) | | 2177.78 (1902.33 to 2510.64) | 0.00 (0.00 to 0.01) | 40.68 (20.84 to 74.37) | | 40.72 (20.81 to 74.69) | 0.00 (0.00 to 0.01) |
| Kuwait | 11863.18 (10321.73 to 13419.45) | 12012.63 (10436.53 to 13567.28) | 0.05 (0.03 to 0.06) | 4667.18 (4112.83 to 5300.22) | | 4718.47 (4155.84 to 5354.61) | 0.04 (0.03 to 0.05) | 91.82 (47.25 to 166.28) | | 92.75 (48.03 to 168.06) | 0.04 (0.03 to 0.05) |
| Kyrgyzstan | 10484.54 (9137.50 to 11802.97) | 10472.86 (9126.37 to 11788.95) | 0.00 (0.00 to 0.00) | 4172.32 (3663.23 to 4766.95) | | 4169.01 (3658.41 to 4764.83) | 0.00 (0.00 to 0.00) | 80.90 (41.31 to 146.38) | | 81.01 (41.34 to 146.37) | 0.01 (0.01 to 0.01) |
| Lao People's Democratic Republic | 5304.42 (4575.46 to 6067.32) | 5295.61 (4571.58 to 6056.58) | -0.01 (-0.01 to -0.01) | 2172.98 (1898.17 to 2504.84) | | 2169.43 (1895.41 to 2499.71) | -0.01 (-0.01 to -0.01) | 40.81 (20.80 to 75.02) | | 40.85 (20.91 to 74.80) | 0.01 (0.00 to 0.01) |
| Latvia | 11326.92 (9937.87 to 12753.23) | 11294.18 (9915.31 to 12718.93) | -0.01 (-0.01 to -0.01) | 4431.52 (3891.24 to 5040.71) | | 4421.48 (3884.31 to 5029.60) | -0.01 (-0.01 to -0.01) | 86.83 (44.53 to 154.59) | | 87.02 (44.59 to 155.70) | 0.01 (0.01 to 0.01) |
| Lebanon | 12164.43 (10568.57 to 13731.74) | 12200.07 (10596.55 to 13759.66) | 0.01 (0.01 to 0.01) | 4767.31 (4204.90 to 5402.43) | | 4777.96 (4222.24 to 5412.88) | 0.01 (0.01 to 0.01) | 93.61 (48.59 to 169.56) | | 93.84 (48.60 to 169.69) | 0.01 (0.01 to 0.01) |
| Lesotho | 11045.30 (9636.00 to 12459.73) | 11043.59 (9633.24 to 12462.21) | 0.00 (0.00 to 0.00) | 4391.78 (3854.26 to 5012.33) | | 4390.83 (3857.92 to 5011.65) | 0.00 (0.00 to 0.00) | 84.77 (43.85 to 150.83) | | 83.97 (43.64 to 149.43) | -0.04 (-0.04 to -0.04) |
| Liberia | 11022.70 (9609.97 to 12436.24) | 11015.24 (9607.22 to 12431.25) | 0.00 (0.00 to 0.00) | 4385.33 (3846.18 to 5008.62) | | 4381.95 (3845.32 to 5001.95) | 0.00 (0.00 to 0.00) | 83.88 (43.46 to 149.45) | | 84.12 (43.73 to 149.83) | 0.01 (0.01 to 0.01) |
| Libya | 12022.81 (10451.82 to 13587.26) | 12102.68 (10526.14 to 13666.37) | 0.02 (0.02 to 0.02) | 4719.47 (4166.72 to 5351.19) | | 4746.72 (4190.69 to 5386.31) | 0.02 (0.01 to 0.02) | 92.83 (48.07 to 167.52) | | 93.07 (48.21 to 168.06) | 0.01 (0.00 to 0.01) |
| Lithuania | 11932.16 (10471.05 to 13446.73) | 11934.23 (10449.20 to 13393.52) | 0.00 (0.00 to 0.00) | 4591.62 (4018.35 to 5198.28) | | 4582.48 (4028.19 to 5206.13) | -0.01 (-0.01 to -0.01) | 91.58 (46.91 to 163.94) | | 91.96 (47.19 to 165.83) | 0.02 (0.01 to 0.02) |
| Luxembourg | 7810.22 (6789.23 to 8892.23) | 7791.01 (6771.66 to 8864.24) | -0.01 (-0.01 to -0.01) | 3196.78 (2814.03 to 3657.62) | | 3190.53 (2804.66 to 3652.84) | -0.01 (-0.01 to -0.01) | 60.17 (30.61 to 109.00) | | 60.00 (30.51 to 109.14) | -0.01 (-0.01 to 0.00) |
| Madagascar | 11021.73 (9594.84 to 12437.62) | 11027.16 (9605.55 to 12442.31) | 0.00 (0.00 to 0.00) | 4383.92 (3849.14 to 5004.95) | | 4385.48 (3849.75 to 5005.07) | 0.00 (0.00 to 0.00) | 84.56 (43.80 to 151.31) | | 84.91 (43.73 to 151.91) | 0.02 (0.02 to 0.02) |
| Malawi | 11036.03 (9614.56 to 12450.63) | 11044.89 (9632.86 to 12457.36) | 0.00 (0.00 to 0.00) | 4388.66 (3853.31 to 5009.28) | | 4391.59 (3854.88 to 5012.62) | 0.00 (0.00 to 0.00) | 84.46 (43.57 to 150.92) | | 84.80 (43.98 to 151.79) | 0.03 (0.02 to 0.03) |
| Malaysia | 5294.26 (4570.91 to 6057.17) | 5285.28 (4564.63 to 6047.40) | -0.01 (-0.01 to -0.01) | 2168.93 (1895.33 to 2498.58) | | 2165.35 (1891.98 to 2493.28) | -0.01 (-0.01 to -0.01) | 40.76 (20.83 to 74.53) | | 40.77 (20.94 to 75.49) | 0.00 (0.00 to 0.01) |
| Maldives | 5264.38 (4542.57 to 6030.80) | 5243.59 (4522.52 to 6010.18) | -0.01 (-0.02 to 0.00) | 2157.58 (1883.65 to 2486.04) | | 2148.35 (1876.45 to 2476.52) | -0.01 (-0.02 to 0.00) | 40.62 (20.74 to 74.78) | | 40.60 (20.61 to 74.48) | 0.01 (0.00 to 0.01) |
| Mali | 11037.14 (9615.07 to 12448.17) | 11025.43 (9606.33 to 12438.03) | 0.00 (0.00 to 0.00) | 4389.27 (3850.52 to 5010.76) | | 4385.20 (3847.89 to 5005.43) | 0.00 (0.00 to 0.00) | 84.63 (43.98 to 151.04) | | 84.97 (43.95 to 152.49) | 0.02 (0.02 to 0.02) |
| Malta | 7815.67 (6785.94 to 8898.04) | 7792.34 (6769.20 to 8865.27) | -0.01 (-0.01 to -0.01) | 3199.11 (2816.76 to 3662.23) | | 3190.95 (2805.07 to 3653.52) | -0.01 (-0.01 to -0.01) | 60.29 (30.74 to 109.66) | | 60.10 (30.69 to 109.14) | -0.01 (-0.01 to -0.01) |
| Marshall Islands | 5288.98 (4570.67 to 6053.66) | 5284.55 (4562.74 to 6046.58) | 0.00 (-0.01 to 0.00) | 2166.60 (1893.71 to 2493.96) | | 2165.25 (1891.76 to 2494.21) | 0.00 (0.00 to 0.00) | 40.64 (20.83 to 74.46) | | 40.43 (20.77 to 74.34) | -0.02 (-0.02 to -0.02) |
| Mauritania | 11034.50 (9613.54 to 12448.03) | 11031.02 (9600.72 to 12443.91) | 0.00 (0.00 to 0.00) | 4388.12 (3851.02 to 5008.42) | | 4387.01 (3850.53 to 5007.56) | 0.00 (0.00 to 0.00) | 85.08 (44.32 to 152.54) | | 85.20 (44.23 to 152.01) | 0.01 (0.01 to 0.01) |
| Mauritius | 5302.55 (4577.08 to 6063.16) | 5299.84 (4575.36 to 6061.76) | 0.00 (0.00 to 0.00) | 2171.97 (1897.49 to 2502.06) | | 2170.94 (1896.90 to 2500.62) | 0.00 (0.00 to 0.00) | 40.76 (20.82 to 74.55) | | 40.66 (20.69 to 74.71) | -0.01 (-0.01 to -0.01) |
| Mexico | 15948.74 (14119.50 to 17648.19) | 15949.98 (14121.96 to 17645.72) | 0.00 (0.00 to 0.00) | 6101.58 (5447.12 to 6746.59) | | 6101.00 (5446.55 to 6744.55) | 0.00 (0.00 to 0.00) | 122.58 (64.04 to 220.33) | | 122.74 (64.16 to 220.76) | 0.00 (0.00 to 0.00) |
| Micronesia (Federated States of) | 5288.73 (4568.23 to 6051.77) | 5296.29 (4575.03 to 6058.74) | 0.00 (0.00 to 0.01) | 2166.53 (1892.42 to 2493.12) | | 2169.43 (1895.43 to 2497.94) | 0.00 (0.00 to 0.01) | 40.70 (20.74 to 74.06) | | 40.66 (20.78 to 74.38) | 0.00 (-0.01 to 0.00) |
| Monaco | 7816.29 (6790.06 to 8898.57) | 7806.45 (6784.69 to 8884.38) | 0.00 (0.00 to 0.00) | 3199.39 (2816.29 to 3662.85) | | 3196.24 (2813.30 to 3660.93) | 0.00 (0.00 to 0.00) | 60.40 (30.94 to 109.73) | | 60.22 (30.58 to 108.75) | -0.01 (-0.01 to -0.01) |
| Mongolia | 10456.43 (9105.36 to 11774.21) | 10474.43 (9125.51 to 11789.73) | 0.01 (0.01 to 0.01) | 4162.97 (3656.04 to 4762.93) | | 4169.48 (3658.73 to 4765.74) | 0.01 (0.01 to 0.01) | 80.70 (41.09 to 147.03) | | 80.89 (41.37 to 147.09) | 0.01 (0.01 to 0.01) |
| Montenegro | 9661.74 (8456.56 to 10943.74) | 9656.43 (8452.39 to 10940.46) | 0.00 (0.00 to 0.00) | 3896.24 (3407.53 to 4468.98) | | 3894.31 (3404.86 to 4467.58) | 0.00 (0.00 to 0.00) | 74.52 (38.16 to 134.84) | | 74.39 (38.05 to 134.22) | 0.00 (-0.01 to 0.00) |
| Morocco | 12155.07 (10563.81 to 13718.30) | 12142.82 (10554.67 to 13706.76) | 0.00 (-0.01 to 0.00) | 4764.92 (4205.49 to 5402.10) | | 4760.38 (4201.23 to 5397.62) | 0.00 (-0.01 to 0.00) | 93.75 (48.42 to 169.43) | | 93.56 (48.18 to 169.11) | -0.01 (-0.01 to -0.01) |
| Mozambique | 11051.43 (9623.85 to 12467.80) | 11053.34 (9628.62 to 12469.66) | 0.00 (0.00 to 0.00) | 4394.13 (3853.65 to 5015.36) | | 4394.53 (3855.43 to 5015.65) | 0.00 (0.00 to 0.00) | 84.35 (43.93 to 150.66) | | 84.46 (43.93 to 150.74) | 0.01 (0.00 to 0.01) |
| Myanmar | 5303.02 (4575.85 to 6064.02) | 5315.08 (4588.53 to 6081.63) | 0.01 (0.01 to 0.01) | 2172.32 (1897.75 to 2503.69) | | 2177.10 (1901.32 to 2510.65) | 0.01 (0.01 to 0.01) | 40.76 (20.82 to 74.44) | | 40.92 (21.00 to 74.95) | 0.02 (0.02 to 0.02) |
| Namibia | 11041.01 (9623.10 to 12454.01) | 11049.47 (9629.15 to 12465.42) | 0.00 (0.00 to 0.00) | 4390.12 (3854.85 to 5011.11) | | 4392.70 (3858.32 to 5013.48) | 0.00 (0.00 to 0.00) | 84.57 (43.88 to 151.18) | | 84.45 (43.85 to 151.24) | 0.00 (-0.01 to 0.00) |
| Nauru | 5281.64 (4564.93 to 6050.66) | 5307.11 (4584.16 to 6072.76) | 0.02 (0.02 to 0.03) | 2163.96 (1890.74 to 2491.64) | | 2173.99 (1899.31 to 2504.72) | 0.02 (0.02 to 0.03) | 40.71 (20.62 to 75.28) | | 40.75 (20.76 to 74.28) | 0.01 (0.00 to 0.01) |
| Nepal | 13072.38 (11492.06 to 14620.79) | 13109.28 (11526.51 to 14672.08) | 0.01 (0.01 to 0.01) | 5064.51 (4482.71 to 5713.66) | | 5076.80 (4491.22 to 5720.77) | 0.01 (0.01 to 0.01) | 99.78 (51.68 to 179.99) | | 100.46 (51.93 to 181.31) | 0.03 (0.03 to 0.03) |
| Netherlands | 5616.74 (4881.79 to 6403.16) | 5610.34 (4876.73 to 6393.86) | 0.01 (0.00 to 0.02) | 2469.33 (2165.15 to 2851.09) | | 2467.30 (2162.46 to 2849.71) | 0.01 (0.00 to 0.01) | 43.42 (22.12 to 79.34) | | 43.35 (22.02 to 78.99) | 0.01 (0.00 to 0.02) |
| New Zealand | 9636.64 (8389.47 to 10887.46) | 9639.88 (8393.39 to 10893.21) | 0.01 (0.01 to 0.02) | 3835.31 (3373.27 to 4409.45) | | 3837.03 (3372.85 to 4409.35) | 0.02 (0.01 to 0.02) | 73.78 (37.46 to 133.75) | | 74.01 (37.79 to 133.74) | 0.03 (0.02 to 0.03) |
| Nicaragua | 15948.79 (14145.48 to 17787.91) | 15942.51 (14143.48 to 17777.68) | 0.00 (0.00 to 0.00) | 5980.58 (5316.39 to 6644.67) | | 5976.39 (5311.71 to 6638.89) | 0.00 (0.00 to 0.00) | 122.77 (63.50 to 217.84) | | 122.90 (64.02 to 217.16) | 0.00 (0.00 to 0.01) |
| Niger | 11019.73 (9604.52 to 12434.11) | 11031.56 (9597.66 to 12445.14) | 0.00 (0.00 to 0.01) | 4383.83 (3845.26 to 5004.99) | | 4387.03 (3851.19 to 5007.22) | 0.00 (0.00 to 0.00) | 84.86 (43.89 to 150.99) | | 85.11 (44.32 to 152.15) | 0.02 (0.01 to 0.02) |
| Nigeria | 11418.88 (9950.88 to 12941.05) | 11469.85 (9987.25 to 12985.97) | 0.02 (0.01 to 0.02) | 4545.79 (3981.86 to 5169.58) | | 4563.29 (4000.82 to 5187.06) | 0.01 (0.01 to 0.02) | 87.66 (44.87 to 156.94) | | 88.18 (45.28 to 158.46) | 0.03 (0.02 to 0.03) |
| Niue | 5301.55 (4576.52 to 6062.17) | 5297.51 (4572.98 to 6063.10) | 0.00 (0.00 to 0.00) | 2171.34 (1896.47 to 2500.52) | | 2169.77 (1895.97 to 2498.71) | 0.00 (0.00 to 0.00) | 40.80 (20.93 to 73.98) | | 40.58 (20.91 to 74.63) | -0.02 (-0.02 to -0.02) |
| North Macedonia | 9654.94 (8453.68 to 10935.18) | 9649.06 (8449.16 to 10927.71) | 0.00 (0.00 to 0.00) | 3893.94 (3404.55 to 4465.04) | | 3891.24 (3403.20 to 4463.64) | 0.00 (0.00 to 0.00) | 74.36 (38.13 to 133.60) | | 74.33 (38.31 to 134.38) | 0.00 (0.00 to 0.00) |
| Northern Mariana Islands | 5251.10 (4535.50 to 6005.08) | 5281.29 (4558.55 to 6042.37) | 0.02 (0.01 to 0.02) | 2151.66 (1877.97 to 2477.35) | | 2163.56 (1889.93 to 2491.9) | 0.02 (0.01 to 0.02) | 40.58 (20.76 to 74.62) | | 40.66 (20.55 to 74.69) | 0.01 (0.00 to 0.01) |
| Norway | 5014.45 (4353.65 to 5711.52) | 5082.83 (4399.26 to 5818.16) | 0.07 (0.05 to 0.08) | 2121.75 (1850.54 to 2439.49) | | 2148.18 (1872.32 to 2496.12) | 0.06 (0.05 to 0.07) | 38.51 (19.56 to 68.99) | | 39.10 (19.98 to 71.24) | 0.08 (0.06 to 0.09) |
| Oman | 11877.81 (10340.07 to 13428.35) | 11807.61 (10282.60 to 13351.54) | -0.03 (-0.05 to -0.01) | 4667.51 (4124.35 to 5302.79) | | 4643.82 (4103.37 to 5278.75) | -0.03 (-0.04 to -0.01) | 91.61 (47.48 to 165.47) | | 91.09 (46.60 to 165.10) | -0.03 (-0.05 to -0.01) |
| Pakistan | 13342.78 (11762.37 to 14896.93) | 13359.46 (11775.69 to 14908.83) | 0.00 (0.00 to 0.00) | 5223.63 (4638.21 to 5898.19) | | 5229.41 (4642.48 to 5904.41) | 0.00 (0.00 to 0.00) | 102.26 (52.82 to 183.61) | | 102.28 (52.44 to 185.09) | 0.00 (0.00 to 0.00) |
| Palau | 5288.78 (4567.58 to 6050.55) | 5270.85 (4550.23 to 6034.27) | -0.01 (-0.01 to 0.00) | 2166.69 (1893.2 to 2495.95) | | 2159.22 (1885.78 to 2486.79) | -0.01 (-0.01 to 0.00) | 40.71 (20.70 to 75.07) | | 40.41 (20.58 to 73.88) | -0.02 (-0.03 to -0.02) |
| Palestine | 12201.24 (10598.45 to 13756.34) | 12135.31 (10553.92 to 13700.65) | -0.02 (-0.02 to -0.01) | 4778.94 (4224.04 to 5414.58) | | 4756.92 (4198.92 to 5394.88) | -0.01 (-0.02 to -0.01) | 93.81 (48.50 to 170.44) | | 93.14 (48.33 to 168.18) | -0.03 (-0.03 to -0.02) |
| Panama | 15921.01 (14127.10 to 17767.17) | 15924.40 (14130.24 to 17772.53) | 0.00 (0.00 to 0.00) | 5972.32 (5314.16 to 6642.42) | | 5972.34 (5312.65 to 6640.49) | 0.00 (0.00 to 0.00) | 122.94 (63.98 to 218.6) | | 122.93 (64.06 to 217.39) | 0.00 (0.00 to 0.00) |
| Papua New Guinea | 5282.28 (4562.76 to 6046.66) | 5280.57 (4559.93 to 6044.76) | 0.00 (0.00 to 0.00) | 2164.11 (1891.37 to 2492.55) | | 2163.51 (1890.75 to 2493.68) | 0.00 (0.00 to 0.00) | 40.47 (20.72 to 74.18) | | 40.43 (20.54 to 73.60) | 0.00 (0.00 to 0.00) |
| Paraguay | 16314.15 (14397.45 to 18170.37) | 16310.51 (14395.46 to 18167.80) | 0.00 (0.00 to 0.00) | 6070.47 (5388.74 to 6729.56) | | 6069.06 (5387.11 to 6728.66) | 0.00 (0.00 to 0.00) | 126.01 (65.35 to 223.25) | | 125.91 (65.11 to 223.26) | 0.00 (0.00 to 0.00) |
| Peru | 15933.38 (14134.59 to 17779.73) | 15932.34 (14135.52 to 17773.58) | 0.00 (0.00 to 0.00) | 5975.91 (5315.09 to 6645.11) | | 5974.10 (5312.59 to 6640.55) | 0.00 (0.00 to 0.00) | 123.09 (64.02 to 217.93) | | 123.26 (64.08 to 218.57) | 0.01 (0.01 to 0.01) |
| Philippines | 5549.45 (4827.69 to 6308.53) | 5550.86 (4830.54 to 6311.02) | 0.00 (0.00 to 0.00) | 2249.51 (1954.51 to 2592.35) | | 2249.76 (1953.96 to 2592.80) | 0.00 (0.00 to 0.00) | 42.61 (21.55 to 76.45) | | 42.73 (21.68 to 76.63) | 0.01 (0.01 to 0.02) |
| Poland | 13682.67 (12013.64 to 15329.30) | 13668.06 (11998.99 to 15316.07) | 0.00 (0.00 to 0.01) | 5132.06 (4543.64 to 5787.23) | | 5128.38 (4540.05 to 5782.80) | 0.00 (0.00 to 0.00) | 104.90 (53.65 to 185.41) | | 105.34 (53.64 to 185.83) | 0.02 (0.02 to 0.03) |
| Portugal | 8457.90 (7296.38 to 9630.73) | 8452.74 (7292.76 to 9628.74) | 0.00 (0.00 to 0.01) | 3379.53 (2949.89 to 3887.10) | | 3377.95 (2947.14 to 3884.48) | 0.00 (0.00 to 0.00) | 65.06 (33.03 to 117.02) | | 65.16 (33.15 to 115.74) | 0.02 (0.01 to 0.02) |
| Puerto Rico | 15951.16 (14147.85 to 17785.38) | 15947.10 (14144.94 to 17782.26) | 0.00 (0.00 to 0.00) | 5980.14 (5315.23 to 6642.27) | | 5978.35 (5313.22 to 6640.16) | 0.00 (0.00 to 0.00) | 123.13 (64.08 to 219.20) | | 122.96 (63.89 to 217.75) | 0.00 (-0.01 to 0.00) |
| Qatar | 11677.95 (10183.92 to 13236.27) | 11536.01 (10067.61 to 13070.27) | -0.06 (-0.08 to -0.05) | 4600.12 (4058.06 to 5231.51) | | 4552.99 (4008.35 to 5179.05) | -0.06 (-0.07 to -0.05) | 90.00 (46.55 to 163.01) | | 88.75 (45.71 to 160.37) | -0.07 (-0.08 to -0.06) |
| Republic of Korea | 6802.59 (5958.48 to 7784.93) | 6784.53 (5942.11 to 7757.37) | 0.48 (0.16 to 0.79) | 2801.86 (2455.35 to 3241.54) | | 2797.25 (2451.56 to 3239.66) | 0.35 (0.11 to 0.58) | 52.40 (26.58 to 94.47) | | 52.51 (26.67 to 94.45) | 0.49 (0.18 to 0.81) |
| Republic of Moldova | 11311.88 (9926.08 to 12737.36) | 11288.07 (9910.59 to 12710.21) | -0.01 (-0.01 to -0.01) | 4428.24 (3886.75 to 5036.35) | | 4419.87 (3881.12 to 5028.36) | -0.01 (-0.01 to -0.01) | 86.87 (44.27 to 154.64) | | 86.99 (44.31 to 154.88) | 0.01 (0.00 to 0.01) |
| Romania | 9659.83 (8455.45 to 10946.92) | 9657.15 (8453.25 to 10940.9) | 0.00 (0.00 to 0.00) | 3895.55 (3407.09 to 4468.58) | | 3894.33 (3406.29 to 4468.68) | 0.00 (0.00 to 0.00) | 74.28 (38.04 to 133.57) | | 74.51 (38.09 to 135.00) | 0.01 (0.01 to 0.02) |
| Russian Federation | 11232.73 (9858.86 to 12650.75) | 11220.55 (9860.61 to 12639.47) | -0.31 (-0.48 to -0.14) | 4447.45 (3909.14 to 5054.19) | | 4443.44 (3906.61 to 5057.64) | -0.25 (-0.39 to -0.11) | 85.93 (43.68 to 156.68) | | 86.19 (43.95 to 157.49) | -0.30 (-0.46 to -0.13) |
| Rwanda | 11046.39 (9612.52 to 12465.47) | 11051.27 (9623.44 to 12471.20) | 0.00 (0.00 to 0.00) | 4391.80 (3857.67 to 5013.36) | | 4393.23 (3858.33 to 5013.47) | 0.00 (0.00 to 0.00) | 84.57 (43.85 to 150.43) | | 84.81 (43.99 to 152.08) | 0.02 (0.01 to 0.03) |
| Saint Kitts and Nevis | 15939.77 (14137.51 to 17781.89) | 15925.94 (14129.4 to 17770.38) | 0.00 (0.00 to 0.00) | 5975.89 (5310.52 to 6639.03) | | 5973.68 (5313.64 to 6641.96) | 0.00 (0.00 to 0.00) | 122.92 (63.97 to 218.51) | | 122.76 (63.87 to 217.32) | 0.00 (0.00 to 0.00) |
| Saint Lucia | 15947.95 (14143.96 to 17785.63) | 15929.51 (14133.54 to 17774.42) | 0.00 (0.00 to 0.00) | 5978.85 (5312.94 to 6640.73) | | 5973.83 (5312.73 to 6642.47) | 0.00 (0.00 to 0.00) | 122.80 (63.76 to 217.12) | | 122.59 (63.82 to 216.58) | 0.00 (-0.01 to 0.00) |
| Saint Vincent and the Grenadines | 15932.32 (14136.03 to 17774.94) | 15917.08 (14124.54 to 17764.46) | 0.00 (0.00 to 0.00) | 5973.43 (5310.29 to 6638.20) | | 5971.15 (5314.01 to 6641.51) | 0.00 (0.00 to 0.00) | 123.01 (64.03 to 218.03) | | 122.64 (63.87 to 217.49) | -0.01 (-0.01 to -0.01) |
| Samoa | 5288.49 (4567.08 to 6049.4) | 5288.13 (4569.30 to 6053.41) | 0.00 (0.00 to 0.00) | 2166.39 (1892.73 to 2496.15) | | 2166.30 (1893.48 to 2494.21) | 0.00 (0.00 to 0.00) | 40.78 (20.83 to 74.14) | | 40.68 (20.66 to 74.96) | -0.01 (-0.01 to -0.01) |
| San Marino | 7799.23 (6776.85 to 8876.11) | 7818.94 (6791.87 to 8900.61) | 0.01 (0.01 to 0.01) | 3193.61 (2808.28 to 3655.19) | | 3201.18 (2818.89 to 3667.35) | 0.01 (0.01 to 0.01) | 60.27 (30.65 to 108.89) | | 60.33 (30.73 to 109.56) | 0.00 (0.00 to 0.00) |
| Sao Tome and Principe | 11038.27 (9611.55 to 12449.83) | 11021.38 (9607.17 to 12439.01) | -0.01 (-0.01 to -0.01) | 4388.92 (3852.75 to 5008.22) | | 4383.72 (3849.21 to 5003.76) | 0.00 (-0.01 to 0.00) | 85.20 (43.93 to 152.38) | | 85.05 (44 to 151.63) | -0.01 (-0.01 to -0.01) |
| Saudi Arabia | 11907.64 (10361.33 to 13460.43) | 11914.66 (10361.70 to 13458.51) | 0.00 (0.00 to 0.01) | 4680.37 (4129.10 to 5311.57) | | 4683.42 (4130.31 to 5313.92) | 0.00 (0.00 to 0.01) | 91.66 (47.67 to 164.85) | | 91.6 (47.6 to 164.95) | 0.00 (-0.01 to 0.00) |
| Senegal | 11031.81 (9604.14 to 12443.67) | 11032.96 (9606.29 to 12446.74) | 0.00 (0.00 to 0.00) | 4387.49 (3849.34 to 5008.74) | | 4387.16 (3851.80 to 5007.26) | 0.00 (0.00 to 0.00) | 84.76 (44.06 to 151.11) | | 84.91 (44.04 to 151.92) | 0.01 (0.01 to 0.01) |
| Serbia | 9657.33 (8453.67 to 10940.90) | 9654.71 (8452.82 to 10938.26) | 0.00 (0.00 to 0.00) | 3894.75 (3404.96 to 4467.47) | | 3893.62 (3404.47 to 4466.44) | 0.00 (0.00 to 0.00) | 74.42 (37.99 to 134.27) | | 74.33 (38.08 to 134.55) | 0.00 (0.00 to 0.00) |
| Seychelles | 5300.81 (4580.51 to 6061.84) | 5281.37 (4560.19 to 6043.54) | -0.02 (-0.02 to -0.01) | 2171.19 (1897.10 to 2499.80) | | 2163.42 (1890.17 to 2490.89) | -0.01 (-0.02 to -0.01) | 40.98 (20.91 to 74.97) | | 40.70 (20.85 to 74.85) | -0.02 (-0.03 to -0.02) |
| Sierra Leone | 11028.44 (9606.41 to 12440.93) | 11022.15 (9607.52 to 12436.7) | 0.00 (-0.01 to 0.00) | 4386.54 (3849.19 to 5008.24) | | 4384.24 (3847.04 to 5004.14) | 0.00 (-0.01 to 0.00) | 84.65 (43.70 to 151.23) | | 84.85 (43.90 to 151.72) | 0.01 (0.01 to 0.01) |
| Singapore | 7286.65 (6355.91 to 8224.79) | 7335.27 (6373.68 to 8317.94) | 0.05 (0.03 to 0.06) | 2943.68 (2570.47 to 3366.67) | | 2958.38 (2594.24 to 3397.14) | 0.04 (0.02 to 0.05) | 56.34 (28.66 to 102.24) | | 56.94 (29.19 to 101.88) | 0.06 (0.05 to 0.08) |
| Slovakia | 9664.68 (8456.97 to 10950.28) | 9657.70 (8452.82 to 10942.84) | 0.00 (0.00 to 0.00) | 3897.71 (3408.22 to 4470.39) | | 3894.65 (3406.53 to 4468.72) | 0.00 (0.00 to 0.00) | 74.27 (37.94 to 133.56) | | 74.33 (38.13 to 134.35) | 0.00 (0.00 to 0.00) |
| Slovenia | 9667.09 (8459.71 to 10954.52) | 9649.88 (8447.72 to 10929.97) | -0.01 (-0.01 to -0.01) | 3897.99 (3411.71 to 4471.65) | | 3891.64 (3403.79 to 4464.47) | -0.01 (-0.01 to -0.01) | 74.17 (38.05 to 133.45) | | 74.25 (37.97 to 133.80) | 0.00 (0.00 to 0.00) |
| Solomon Islands | 5270.97 (4548.37 to 6034.73) | 5288.83 (4564.61 to 6052.71) | 0.01 (0.01 to 0.01) | 2159.99 (1886.14 to 2488.78) | | 2166.86 (1893.43 to 2497.19) | 0.01 (0.01 to 0.01) | 40.62 (20.58 to 74.58) | | 40.62 (20.59 to 74.24) | 0.00 (0.00 to 0.00) |
| Somalia | 11031.91 (9614.26 to 12447.77) | 11037.38 (9626.07 to 12456.86) | 0.00 (0.00 to 0.00) | 4386.67 (3853.32 to 5006.64) | | 4388.47 (3856.59 to 5008.63) | 0.00 (0.00 to 0.00) | 84.50 (43.63 to 150.48) | | 84.62 (43.81 to 150.69) | 0.01 (0.01 to 0.01) |
| South Africa | 11472.91 (9991.45 to 12986.59) | 11467.17 (9985.68 to 12982.60) | 0.00 (0.00 to 0.00) | 4563.87 (4001.04 to 5192.76) | | 4561.36 (3998.74 to 5189.60) | 0.00 (0.00 to 0.00) | 87.85 (45.08 to 157.30) | | 87.46 (45.04 to 156.38) | -0.02 (-0.02 to -0.01) |
| South Sudan | 10984.47 (9578.79 to 12405.46) | 11029.44 (9603.12 to 12438.37) | 0.01 (0.01 to 0.02) | 4371.43 (3835.18 to 4987.55) | | 4386.25 (3847.68 to 5005.94) | 0.01 (0.01 to 0.01) | 83.70 (43.25 to 148.63) | | 84.09 (43.50 to 149.57) | 0.02 (0.01 to 0.02) |
| Spain | 7011.94 (6095.10 to 7963.25) | 7000.31 (6082.16 to 7946.51) | 0.07 (-0.09 to 0.24) | 2944.89 (2562.72 to 3404.10) | | 2941.01 (2559.04 to 3399.83) | 0.06 (-0.07 to 0.19) | 54.12 (27.58 to 97.51) | | 54.06 (27.50 to 97.40) | 0.08 (-0.09 to 0.24) |
| Sri Lanka | 5290.90 (4567.03 to 6052.99) | 5309.03 (4582.23 to 6072.13) | 0.01 (0.01 to 0.01) | 2167.71 (1894.07 to 2498.39) | | 2174.69 (1899.65 to 2506.79) | 0.01 (0.01 to 0.01) | 40.77 (20.93 to 74.66) | | 40.85 (20.85 to 74.64) | 0.01 (0.01 to 0.01) |
| Sudan | 12133.81 (10548.65 to 13701.29) | 12118.61 (10535.58 to 13691.27) | 0.00 (-0.01 to 0.00) | 4758.77 (4194.62 to 5395.84) | | 4753.35 (4189.23 to 5389.46) | 0.00 (0.00 to 0.00) | 93.59 (48.14 to 169.19) | | 93.45 (48.38 to 169.37) | 0.00 (-0.01 to 0.00) |
| Suriname | 15927.64 (14135.60 to 17767.14) | 15937.19 (14137.12 to 17777.55) | 0.00 (0.00 to 0.00) | 5972.36 (5311.66 to 6638.83) | | 5975.94 (5312.85 to 6643.75) | 0.00 (0.00 to 0.00) | 122.87 (63.86 to 216.91) | | 122.52 (63.63 to 216.27) | -0.01 (-0.01 to -0.01) |
| Sweden | 6688.55 (5848.49 to 7562.39) | 7124.05 (6180.66 to 8116.26) | 0.15 (0.07 to 0.23) | 2798.17 (2438.21 to 3223.40) | | 2947.12 (2564.19 to 3405.32) | 0.12 (0.06 to 0.19) | 51.64 (26.24 to 93.44) | | 55.00 (28.02 to 99.92) | 0.16 (0.08 to 0.24) |
| Switzerland | 4660.12 (4046.51 to 5304.87) | 4649.86 (4038.28 to 5292.21) | 0.16 (0.09 to 0.23) | 2110.40 (1853.84 to 2437.52) | | 2106.58 (1850.10 to 2435.22) | 0.14 (0.08 to 0.21) | 35.78 (18.47 to 64.94) | | 35.84 (18.35 to 65.01) | 0.18 (0.10 to 0.25) |
| Syrian Arab Republic | 12107.84 (10527.73 to 13672.08) | 12189.29 (10587.24 to 13764.69) | 0.02 (0.01 to 0.03) | 4749.27 (4189.68 to 5388.94) | | 4778.11 (4210.21 to 5414.37) | 0.02 (0.01 to 0.03) | 93.57 (48.08 to 168.91) | | 93.73 (48.28 to 169.47) | 0.01 (0.00 to 0.01) |
| Taiwan (Province of China) | 4970.50 (4322.53 to 5635.88) | 5150.38 (4464.23 to 5858.15) | 0.19 (0.11 to 0.26) | 2024.02 (1748.70 to 2316.06) | | 2077.02 (1809.67 to 2394.84) | 0.14 (0.09 to 0.20) | 38.54 (19.70 to 70.34) | | 40.02 (20.29 to 71.77) | 0.19 (0.12 to 0.27) |
| Tajikistan | 10463.64 (9111.76 to 11781.85) | 10451.87 (9098.01 to 11764.79) | 0.00 (0.00 to 0.00) | 4164.83 (3657.15 to 4763.35) | | 4161.98 (3656.66 to 4761.54) | 0.00 (0.00 to 0.00) | 80.92 (41.25 to 146.13) | | 80.75 (41.31 to 145.44) | 0.00 (-0.01 to 0.00) |
| Thailand | 5302.96 (4575.73 to 6064.06) | 5303.14 (4575.94 to 6063.49) | 0.00 (0.00 to 0.00) | 2172.31 (1897.68 to 2503.89) | | 2172.37 (1897.65 to 2503.57) | 0.00 (0.00 to 0.00) | 40.84 (20.80 to 74.88) | | 41.01 (21.03 to 75.29) | 0.02 (0.02 to 0.02) |
| Timor-Leste | 5285.47 (4565.01 to 6049.13) | 5291.01 (4566.15 to 6056.99) | 0.00 (0.00 to 0.00) | 2165.38 (1892.38 to 2495.15) | | 2167.66 (1894.74 to 2497.61) | 0.00 (0.00 to 0.00) | 40.61 (20.73 to 73.98) | | 40.71 (20.80 to 74.91) | 0.02 (0.01 to 0.04) |
| Togo | 11048.32 (9613.18 to 12466.89) | 11055.75 (9638.41 to 12476.86) | 0.00 (0.00 to 0.00) | 4392.44 (3854.67 to 5012.84) | | 4394.47 (3857.48 to 5013.79) | 0.00 (0.00 to 0.00) | 84.96 (43.95 to 151.48) | | 85.21 (44.05 to 151.9) | 0.01 (0.01 to 0.01) |
| Tokelau | 5311.57 (4584.05 to 6078.16) | 5286.48 (4563.41 to 6049.76) | -0.02 (-0.02 to -0.02) | 2176.00 (1900.04 to 2509.22) | | 2165.86 (1892.30 to 2495.68) | -0.02 (-0.02 to -0.02) | 40.96 (20.86 to 74.33) | | 40.68 (20.68 to 74.66) | -0.02 (-0.03 to -0.02) |
| Tonga | 5300.55 (4572.53 to 6065.46) | 5303.22 (4576.36 to 6066.05) | 0.00 (0.00 to 0.00) | 2171.53 (1896.55 to 2504.63) | | 2172.41 (1898.00 to 2505.55) | 0.00 (0.00 to 0.00) | 40.89 (20.80 to 74.37) | | 40.82 (20.78 to 74.4) | 0.00 (-0.01 to 0.00) |
| Trinidad and Tobago | 15929.00 (14132.51 to 17775.90) | 15923.10 (14128.04 to 17770.08) | 0.00 (0.00 to 0.00) | 5973.68 (5312.27 to 6641.60) | | 5972.06 (5311.97 to 6639.71) | 0.00 (0.00 to 0.00) | 122.89 (64.09 to 217.88) | | 122.68 (63.84 to 217.85) | 0.00 (0.00 to 0.00) |
| Tunisia | 12130.96 (10547.14 to 13695.32) | 12159.64 (10565.70 to 13724.44) | 0.01 (0.01 to 0.01) | 4756.96 (4197.06 to 5395.48) | | 4765.92 (4205.95 to 5403.48) | 0.01 (0.01 to 0.01) | 93.88 (48.29 to 168.94) | | 93.89 (48.29 to 169.17) | 0.00 (0.00 to 0.00) |
| Turkey | 13346.28 (11668.33 to 15000.21) | 14226.80 (13132.37 to 15338.41) | 0.30 (0.23 to 0.37) | 5057.71 (4454.92 to 5709.69) | | 5248.27 (4687.28 to 5842.60) | 0.17 (0.13 to 0.20) | 102.95 (53.05 to 185.39) | | 109.67 (57.20 to 198.02) | 0.29 (0.22 to 0.36) |
| Turkmenistan | 10478.21 (9129.18 to 11796.73) | 10459.35 (9106.55 to 11773.59) | -0.01 (-0.01 to -0.01) | 4170.14 (3661.66 to 4765.70) | | 4163.57 (3658.07 to 4758.40) | 0.00 (-0.01 to 0.00) | 81.02 (41.34 to 146.66) | | 80.93 (41.25 to 146.86) | 0.00 (0.00 to 0.00) |
| Tuvalu | 5323.99 (4597.42 to 6099.60) | 5291.57 (4572.96 to 6054.31) | -0.02 (-0.02 to -0.02) | 2180.58 (1904.17 to 2514.44) | | 2167.53 (1893.83 to 2495.51) | -0.02 (-0.02 to -0.02) | 41.01 (20.83 to 75.15) | | 40.65 (20.79 to 73.97) | -0.03 (-0.03 to -0.03) |
| Uganda | 11030.88 (9609.69 to 12445.79) | 11050.46 (9627.90 to 12467.45) | 0.01 (0.01 to 0.01) | 4387.14 (3850.54 to 5007.85) | | 4393.10 (3857.10 to 5013.54) | 0.00 (0.00 to 0.01) | 84.11 (43.73 to 149.93) | | 84.93 (44.11 to 151.64) | 0.04 (0.04 to 0.04) |
| Ukraine | 11678.51 (10198.96 to 13060.93) | 11664.72 (10187.24 to 13045.10) | 0.00 (0.00 to 0.00) | 4612.41 (4054.26 to 5226.03) | | 4608.28 (4050.17 to 5221.74) | 0.00 (0.00 to 0.00) | 89.61 (45.91 to 162.41) | | 89.79 (46.08 to 163.81) | 0.01 (0.01 to 0.02) |
| United Arab Emirates | 11701.97 (10190.67 to 13234.00) | 11624.76 (10132.80 to 13163.22) | -0.03 (-0.04 to -0.03) | 4609.02 (4064.42 to 5242.05) | | 4586.93 (4035.18 to 5218.52) | -0.02 (-0.03 to -0.02) | 90.29 (46.71 to 163.88) | | 89.59 (46.30 to 161.86) | -0.03 (-0.04 to -0.03) |
| United Kingdom | 10390.96 (9079.17 to 11766.56) | 10335.00 (9002.04 to 11713.93) | -0.11 (-0.30 to 0.08) | 4026.73 (3545.66 to 4583.27) | | 4005.90 (3509.66 to 4579.98) | -0.08 (-0.22 to 0.06) | 79.81 (41.09 to 143.87) | | 79.31 (40.78 to 142.97) | -0.11 (-0.29 to 0.08) |
| United Republic of Tanzania | 11039.52 (9613.17 to 12451.85) | 11039.84 (9614.60 to 12452.00) | 0.00 (0.00 to 0.00) | 4390.13 (3852.06 to 5012.04) | | 4390.11 (3851.68 to 5010.98) | 0.00 (0.00 to 0.00) | 84.37 (43.81 to 150.23) | | 84.85 (43.98 to 151.18) | 0.03 (0.02 to 0.03) |
| United States of America | 10993.58 (9577.18 to 12428.79) | 9659.80 (8405.19 to 10935.68) | -0.89 (-1.10 to -0.68) | 4216.00 (3666.27 to 4788.30) | | 3810.93 (3325.21 to 4379.04) | -0.71 (-0.89 to -0.54) | 83.90 (43.19 to 150.27) | | 73.50 (37.17 to 131.47) | -0.90 (-1.11 to -0.69) |
| United States Virgin Islands | 15953.83 (14148.61 to 17787.89) | 15955.27 (14149.14 to 17786.14) | 0.00 (0.00 to 0.00) | 5981.71 (5316.27 to 6644.32) | | 5981.26 (5314.65 to 6643.68) | 0.00 (0.00 to 0.00) | 123.50 (64.22 to 219.79) | | 123.20 (63.97 to 218.49) | -0.01 (-0.01 to -0.01) |
| Uruguay | 13628.57 (11864.93 to 15357.56) | 13631.52 (11868.97 to 15361.11) | 0.00 (0.00 to 0.00) | 5003.18 (4406.71 to 5687.51) | | 5003.96 (4407.45 to 5688.29) | 0.00 (0.00 to 0.00) | 105.28 (54.17 to 189.52) | | 105.19 (54.32 to 187.87) | 0.00 (0.00 to 0.00) |
| Uzbekistan | 10474.77 (9126.54 to 11794.60) | 10467.93 (9122.81 to 11784.40) | 0.00 (0.00 to 0.00) | 4168.74 (3660.32 to 4763.96) | | 4167.27 (3657.40 to 4763.65) | 0.00 (0.00 to 0.00) | 80.88 (41.57 to 147.20) | | 80.83 (41.40 to 145.85) | 0.00 (0.00 to 0.00) |
| Vanuatu | 5274.43 (4554.36 to 6038.77) | 5285.43 (4561.70 to 6049.72) | 0.01 (0.01 to 0.01) | 2161.30 (1887.65 to 2489.82) | | 2165.60 (1891.80 to 2495.65) | 0.01 (0.01 to 0.01) | 40.57 (20.68 to 73.95) | | 40.59 (20.78 to 73.86) | 0.00 (0.00 to 0.01) |
| Venezuela (Bolivarian Republic of) | 15934.83 (14136.05 to 17778.18) | 15941.49 (14138.75 to 17783.93) | 0.00 (0.00 to 0.00) | 5975.60 (5313.56 to 6643.72) | | 5978.01 (5314.78 to 6644.69) | 0.00 (0.00 to 0.00) | 122.94 (64.14 to 217.04) | | 123.03 (63.74 to 217.04) | 0.00 (0.00 to 0.00) |
| Viet Nam | 5319.57 (4593.02 to 6089.07) | 5307.52 (4581.83 to 6068.58) | -0.01 (-0.01 to -0.01) | 2178.79 (1902.53 to 2512.29) | | 2173.79 (1899.16 to 2503.79) | -0.01 (-0.01 to -0.01) | 41.09 (21.08 to 75.13) | | 41.08 (21.03 to 75.32) | 0.00 (0.00 to 0.00) |
| Yemen | 12150.94 (10566.77 to 13718.77) | 12144.72 (10557.29 to 13707.90) | 0.00 (0.00 to 0.00) | 4762.14 (4200.31 to 5400.78) | | 4760.94 (4202.96 to 5400.36) | 0.00 (0.00 to 0.00) | 93.22 (48.09 to 168.01) | | 93.27 (47.96 to 169.35) | 0.01 (0.00 to 0.01) |
| Zambia | 11022.69 (9593.19 to 12437.08) | 11027.46 (9619.57 to 12443.53) | 0.00 (0.00 to 0.00) | 4384.54 (3848.13 to 5006.26) | | 4385.96 (3850.75 to 5006.52) | 0.00 (0.00 to 0.00) | 84.49 (43.80 to 150.52) | | 84.55 (43.64 to 150.77) | 0.01 (0.00 to 0.01) |
| Zimbabwe | 11036.32 (9616.78 to 12447.10) | 11059.06 (9641.10 to 12485.05) | 0.01 (0.01 to 0.01) | 4388.85 (3849.22 to 5008.99) | | 4396.02 (3861.56 to 5017.81) | 0.01 (0.01 to 0.01) | 84.79 (43.84 to 152.1) | | 84.63 (43.91 to 150.81) | 0.00 (-0.01 to 0.00) |

Abbreviations: CI, confidence interval; EAPC, estimated annual percentage change; UI, uncertainty interval; YLD, years of life lived with disability.

**Supplementary Table 6** EAPC of age-standardised prevalence, incidence, and YLD rates for gastroesophageal reflux disease (GERD) by 204 countries or territories from 2015 to 2019

| Characteristics | EAPC of ASPR (95%CI) | EAPC of ASIR (95%CI) | EAPC of ASYR (95%CI) |
| --- | --- | --- | --- |
| Afghanistan | -0.02 (-0.02 to -0.02) | -0.02 (-0.02 to -0.02) | -0.03 (-0.04 to -0.01) |
| Albania | 0.33 (-0.05 to 0.72) | 0.25 (-0.04 to 0.54) | 0.30 (-0.08 to 0.69) |
| Algeria | 0.00 (0.00 to 0.00) | 0.00 (0.00 to 0.00) | -0.01 (-0.03 to 0.00) |
| American Samoa | 0.01 (0.01 to 0.01) | 0.01 (0.01 to 0.01) | -0.03 (-0.04 to -0.01) |
| Andorra | -0.01 (-0.02 to -0.01) | -0.01 (-0.01 to -0.01) | -0.02 (-0.05 to 0.01) |
| Angola | 0.01 (0.00 to 0.01) | 0.00 (0.00 to 0.00) | 0.01 (0.00 to 0.02) |
| Antigua and Barbuda | -0.01 (-0.01 to -0.01) | -0.01 (-0.01 to -0.01) | -0.03 (-0.04 to -0.01) |
| Argentina | 0.00 (0.00 to 0.00) | 0.00 (0.00 to 0.00) | -0.02 (-0.03 to -0.01) |
| Armenia | 0.00 (0.00 to 0.00) | 0.00 (0.00 to 0.00) | 0.00 (-0.02 to 0.01) |
| Australia | 0.00 (0.00 to 0.00) | 0.00 (0.00 to 0.00) | -0.03 (-0.05 to 0.00) |
| Austria | -0.05 (-0.09 to -0.01) | -0.04 (-0.07 to -0.01) | -0.04 (-0.10 to 0.01) |
| Azerbaijan | 0.00 (0.00 to 0.00) | 0.00 (0.00 to 0.00) | -0.02 (-0.04 to 0.01) |
| Bahamas | 0.00 (0.00 to 0.00) | 0.00 (0.00 to 0.00) | -0.01 (-0.02 to 0.00) |
| Bahrain | 0.02 (0.01 to 0.04) | 0.03 (0.02 to 0.04) | 0.01 (0.00 to 0.03) |
| Bangladesh | 1.32 (0.06 to 2.60) | 0.88 (0.04 to 1.73) | 1.33 (0.03 to 2.64) |
| Barbados | 0.00 (0.00 to 0.00) | 0.00 (0.00 to 0.00) | -0.02 (-0.03 to -0.01) |
| Belarus | -0.01 (-0.01 to -0.01) | -0.01 (-0.01 to -0.01) | 0.02 (-0.01 to 0.05) |
| Belgium | -0.04 (-0.08 to -0.01) | -0.03 (-0.06 to 0.00) | -0.04 (-0.12 to 0.03) |
| Belize | 0.00 (0.00 to 0.00) | 0.00 (0.00 to 0.00) | -0.01 (-0.02 to 0.00) |
| Benin | -0.01 (-0.01 to -0.01) | 0.00 (-0.01 to 0.00) | 0.01 (0.00 to 0.02) |
| Bermuda | 0.00 (0.00 to 0.00) | 0.00 (0.00 to 0.00) | -0.02 (-0.02 to -0.01) |
| Bhutan | 0.01 (0.01 to 0.01) | 0.01 (0.01 to 0.01) | 0.00 (-0.01 to 0.01) |
| Bolivia (Plurinational State of) | 0.00 (0.00 to 0.00) | 0.00 (0.00 to 0.00) | -0.01 (-0.02 to 0.00) |
| Bosnia and Herzegovina | 0.00 (0.00 to 0.00) | 0.00 (0.00 to 0.00) | -0.02 (-0.02 to -0.01) |
| Botswana | -0.01 (-0.01 to 0.00) | 0.00 (-0.01 to 0.00) | -0.02 (-0.03 to -0.01) |
| Brazil | -0.03 (-0.05 to 0.00) | 0.02 (-0.01 to 0.05) | 0.00 (-0.01 to 0.01) |
| Brunei Darussalam | 0.00 (0.00 to 0.00) | 0.00 (0.00 to 0.00) | 0.02 (-0.02 to 0.05) |
| Bulgaria | 0.00 (0.00 to 0.00) | 0.00 (0.00 to 0.00) | -0.01 (-0.02 to 0.00) |
| Burkina Faso | 0.00 (0.00 to 0.00) | 0.00 (0.00 to 0.00) | 0.01 (-0.01 to 0.02) |
| Burundi | 0.00 (-0.01 to 0.00) | 0.00 (0.00 to 0.00) | 0.02 (0.01 to 0.03) |
| Cabo Verde | -0.01 (-0.02 to -0.01) | -0.01 (-0.01 to -0.01) | -0.01 (-0.02 to 0.01) |
| Cambodia | -0.01 (-0.01 to -0.01) | -0.01 (-0.01 to -0.01) | -0.02 (-0.03 to -0.01) |
| Cameroon | 0.00 (0.00 to 0.00) | 0.00 (0.00 to 0.00) | 0.03 (0.00 to 0.05) |
| Canada | 0.00 (0.00 to 0.00) | 0.00 (0.00 to 0.00) | -0.03 (-0.05 to 0.00) |
| Central African Republic | 0.01 (0.01 to 0.01) | 0.00 (0.00 to 0.01) | 0.02 (0.00 to 0.04) |
| Chad | 0.00 (0.00 to 0.00) | 0.00 (0.00 to 0.00) | 0.01 (-0.02 to 0.03) |
| Chile | 0.00 (0.00 to 0.00) | 0.00 (0.00 to 0.00) | -0.01 (-0.03 to 0.01) |
| China | 2.36 (-0.01 to 4.79) | 2.14 (-0.01 to 4.34) | 2.35 (-0.02 to 4.78) |
| Colombia | 0.00 (0.00 to 0.00) | 0.00 (-0.01 to 0.00) | -0.01 (-0.02 to -0.01) |
| Comoros | 0.00 (0.00 to 0.00) | 0.00 (0.00 to 0.00) | 0.00 (-0.03 to 0.03) |
| Congo | 0.00 (0.00 to 0.00) | 0.00 (0.00 to 0.00) | 0.00 (0.00 to 0.01) |
| Cook Islands | 0.01 (0.01 to 0.01) | 0.01 (0.01 to 0.01) | 0.00 (-0.02 to 0.02) |
| Costa Rica | 0.00 (0.00 to 0.00) | 0.00 (0.00 to 0.00) | 0.00 (-0.02 to 0.01) |
| Côte d'Ivoire | 0.00 (0.00 to 0.00) | 0.00 (0.00 to 0.00) | 0.02 (0.00 to 0.03) |
| Croatia | 0.00 (0.00 to 0.00) | 0.00 (0.00 to 0.00) | -0.01 (-0.02 to -0.01) |
| Cuba | 0.00 (0.00 to 0.00) | 0.00 (0.00 to 0.00) | -0.01 (-0.02 to -0.01) |
| Cyprus | 0.01 (0.01 to 0.01) | 0.01 (0.01 to 0.01) | 0.01 (0.00 to 0.01) |
| Czechia | 0.00 (0.00 to 0.00) | 0.00 (0.00 to 0.00) | 0.00 (-0.03 to 0.04) |
| Democratic People's Republic of Korea | -0.02 (-0.02 to -0.02) | -0.02 (-0.02 to -0.02) | -0.02 (-0.05 to 0.00) |
| Democratic Republic of the Congo | 0.00 (0.00 to 0.00) | 0.00 (0.00 to 0.00) | 0.01 (-0.01 to 0.03) |
| Denmark | 0.08 (0.00 to 0.16) | 0.04 (0.00 to 0.09) | 0.06 (-0.03 to 0.16) |
| Djibouti | 0.00 (0.00 to 0.00) | 0.00 (0.00 to 0.00) | 0.01 (-0.01 to 0.03) |
| Dominica | 0.00 (0.00 to 0.00) | 0.00 (0.00 to 0.00) | -0.02 (-0.03 to 0.00) |
| Dominican Republic | 0.00 (0.00 to 0.00) | 0.00 (0.00 to 0.00) | -0.02 (-0.03 to -0.01) |
| Ecuador | 0.00 (0.00 to 0.00) | 0.00 (0.00 to 0.00) | 0.00 (-0.01 to 0.01) |
| Egypt | -0.01 (-0.01 to -0.01) | -0.01 (-0.01 to -0.01) | -0.02 (-0.04 to 0.00) |
| El Salvador | 0.00 (0.00 to 0.00) | 0.00 (0.00 to 0.00) | 0.00 (-0.01 to 0.01) |
| Equatorial Guinea | -0.02 (-0.03 to -0.02) | -0.02 (-0.02 to -0.02) | -0.03 (-0.04 to -0.01) |
| Eritrea | -0.01 (-0.01 to -0.01) | -0.01 (-0.01 to -0.01) | 0.02 (0.00 to 0.04) |
| Estonia | -0.02 (-0.03 to -0.02) | -0.02 (-0.02 to -0.02) | -0.03 (-0.04 to -0.01) |
| Eswatini | 0.00 (0.00 to 0.00) | 0.00 (-0.01 to 0.00) | -0.01 (-0.02 to -0.01) |
| Ethiopia | 0.00 (0.00 to 0.00) | 0.00 (0.00 to 0.00) | 0.01 (0.00 to 0.03) |
| Fiji | 0.00 (0.00 to 0.00) | 0.00 (0.00 to 0.00) | -0.02 (-0.03 to 0.00) |
| Finland | 0.08 (-0.39 to 0.56) | -0.01 (-0.25 to 0.24) | 0.09 (-0.39 to 0.57) |
| France | -0.11 (-0.21 to 0.00) | -0.06 (-0.12 to 0.00) | -0.12 (-0.19 to -0.04) |
| Gabon | 0.01 (0.01 to 0.01) | 0.01 (0.01 to 0.01) | 0.01 (-0.01 to 0.03) |
| Gambia | 0.00 (0.00 to 0.00) | 0.00 (0.00 to 0.00) | 0.02 (-0.01 to 0.05) |
| Georgia | -0.01 (-0.01 to -0.01) | -0.01 (-0.01 to -0.01) | 0.00 (-0.01 to 0.00) |
| Germany | -0.17 (-0.33 to -0.01) | -0.11 (-0.21 to -0.01) | -0.18 (-0.35 to -0.02) |
| Ghana | 0.01 (0.01 to 0.01) | 0.00 (0.00 to 0.01) | 0.02 (0.01 to 0.03) |
| Greece | 0.00 (0.00 to 0.01) | 0.00 (0.00 to 0.01) | -0.01 (-0.03 to 0.00) |
| Greenland | 0.01 (0.01 to 0.01) | 0.01 (0.01 to 0.02) | 0.00 (-0.02 to 0.02) |
| Grenada | -0.01 (-0.01 to -0.01) | -0.01 (-0.01 to -0.01) | -0.02 (-0.04 to 0.00) |
| Guam | -0.01 (-0.01 to -0.01) | -0.01 (-0.01 to -0.01) | -0.03 (-0.06 to -0.01) |
| Guatemala | 0.00 (0.00 to 0.00) | 0.00 (0.00 to 0.00) | 0.00 (0.00 to 0.01) |
| Guinea | 0.00 (0.00 to 0.00) | 0.00 (0.00 to 0.00) | 0.01 (0.00 to 0.02) |
| Guinea-Bissau | 0.00 (0.00 to 0.00) | 0.00 (0.00 to 0.00) | 0.00 (-0.01 to 0.01) |
| Guyana | 0.00 (0.00 to 0.00) | 0.00 (-0.01 to 0.00) | -0.01 (-0.01 to 0.00) |
| Haiti | 0.00 (0.00 to 0.00) | 0.00 (0.00 to 0.00) | -0.01 (-0.03 to 0.00) |
| Honduras | 0.00 (0.00 to 0.00) | 0.00 (0.00 to 0.00) | -0.01 (-0.03 to 0.01) |
| Hungary | 0.01 (-0.01 to 0.03) | -0.01 (-0.01 to -0.01) | -0.01 (-0.04 to 0.03) |
| Iceland | -0.02 (-0.02 to -0.01) | -0.02 (-0.02 to -0.01) | -0.03 (-0.05 to -0.02) |
| India | -0.18 (-0.35 to 0.00) | -0.10 (-0.19 to 0.00) | -0.17 (-0.34 to 0.00) |
| Indonesia | 0.00 (0.00 to 0.00) | 0.00 (0.00 to 0.00) | -0.01 (-0.01 to 0.00) |
| Iran (Islamic Republic of) | -0.01 (-0.01 to -0.01) | -0.01 (-0.01 to -0.01) | -0.01 (-0.01 to -0.01) |
| Iraq | 0.00 (0.00 to 0.01) | 0.00 (0.00 to 0.01) | 0.00 (-0.01 to 0.02) |
| Ireland | 0.00 (0.00 to 0.00) | 0.00 (0.00 to 0.00) | -0.01 (-0.04 to 0.03) |
| Israel | 0.00 (0.00 to 0.00) | 0.00 (0.00 to 0.00) | 0.00 (-0.01 to 0.01) |
| Italy | -0.20 (-0.40 to 0.01) | -0.14 (-0.29 to 0.01) | -0.23 (-0.43 to -0.03) |
| Jamaica | 0.00 (0.00 to 0.00) | 0.00 (0.00 to 0.00) | -0.03 (-0.04 to -0.02) |
| Japan | 0.02 (0.02 to 0.02) | 0.01 (0.01 to 0.02) | 0.02 (0.00 to 0.04) |
| Jordan | -0.03 (-0.04 to -0.03) | -0.03 (-0.03 to -0.03) | -0.04 (-0.05 to -0.02) |
| Kazakhstan | -0.01 (-0.01 to -0.01) | -0.01 (-0.01 to -0.01) | 0.01 (-0.01 to 0.02) |
| Kenya | 0.00 (0.00 to 0.00) | 0.00 (0.00 to 0.00) | 0.00 (0.00 to 0.00) |
| Kiribati | 0.00 (0.00 to 0.01) | 0.00 (0.00 to 0.00) | 0.00 (-0.02 to 0.02) |
| Kuwait | 0.10 (0.09 to 0.11) | 0.09 (0.08 to 0.09) | 0.07 (0.05 to 0.09) |
| Kyrgyzstan | 0.00 (0.00 to 0.00) | 0.00 (0.00 to 0.00) | -0.01 (-0.02 to -0.01) |
| Lao People's Democratic Republic | 0.00 (0.00 to 0.00) | 0.00 (0.00 to 0.00) | -0.02 (-0.03 to -0.01) |
| Latvia | -0.02 (-0.02 to -0.02) | -0.02 (-0.02 to -0.02) | -0.02 (-0.04 to 0.00) |
| Lebanon | 0.01 (0.01 to 0.01) | 0.01 (0.01 to 0.01) | 0.00 (-0.02 to 0.02) |
| Lesotho | -0.01 (-0.01 to 0.00) | 0.00 (0.00 to 0.00) | 0.00 (-0.02 to 0.01) |
| Liberia | 0.00 (0.00 to 0.00) | 0.00 (0.00 to 0.00) | 0.03 (0.02 to 0.04) |
| Libya | 0.01 (0.00 to 0.01) | 0.00 (0.00 to 0.01) | -0.02 (-0.03 to -0.01) |
| Lithuania | -0.01 (-0.01 to -0.01) | -0.01 (-0.01 to -0.01) | 0.01 (-0.01 to 0.03) |
| Luxembourg | -0.01 (-0.01 to 0.00) | 0.00 (-0.01 to 0.00) | -0.02 (-0.03 to -0.01) |
| Madagascar | 0.00 (0.00 to 0.00) | 0.00 (0.00 to 0.00) | 0.00 (-0.01 to 0.01) |
| Malawi | 0.00 (0.00 to 0.00) | 0.00 (0.00 to 0.00) | 0.00 (-0.03 to 0.03) |
| Malaysia | 0.00 (0.00 to 0.00) | 0.00 (0.00 to 0.00) | 0.01 (-0.03 to 0.04) |
| Maldives | -0.06 (-0.06 to -0.06) | -0.06 (-0.07 to -0.06) | -0.07 (-0.08 to -0.06) |
| Mali | 0.00 (0.00 to 0.00) | 0.00 (0.00 to 0.00) | 0.00 (-0.01 to 0.01) |
| Malta | -0.01 (-0.01 to -0.01) | -0.01 (-0.01 to 0.00) | 0.00 (-0.03 to 0.02) |
| Marshall Islands | 0.01 (0.01 to 0.01) | 0.01 (0.01 to 0.01) | -0.01 (-0.03 to 0.01) |
| Mauritania | 0.00 (0.00 to 0.00) | 0.00 (0.00 to 0.00) | -0.01 (-0.03 to 0.01) |
| Mauritius | -0.01 (-0.01 to 0.00) | 0.00 (-0.01 to 0.00) | -0.01 (-0.03 to 0.00) |
| Mexico | 0.00 (0.00 to 0.00) | 0.00 (0.00 to 0.00) | -0.03 (-0.07 to 0.02) |
| Micronesia (Federated States of) | 0.00 (0.00 to 0.00) | 0.00 (0.00 to 0.00) | -0.03 (-0.04 to -0.03) |
| Monaco | 0.00 (0.00 to 0.00) | 0.00 (0.00 to 0.01) | -0.02 (-0.03 to 0.00) |
| Mongolia | 0.00 (0.00 to 0.00) | 0.00 (0.00 to 0.00) | 0.00 (-0.01 to 0.00) |
| Montenegro | 0.00 (0.00 to 0.00) | 0.00 (0.00 to 0.00) | -0.02 (-0.04 to 0.00) |
| Morocco | -0.01 (-0.01 to -0.01) | -0.01 (-0.01 to -0.01) | -0.02 (-0.03 to 0.00) |
| Mozambique | 0.00 (0.00 to 0.00) | 0.00 (0.00 to 0.00) | -0.01 (-0.03 to 0.00) |
| Myanmar | 0.01 (0.00 to 0.01) | 0.01 (0.00 to 0.01) | -0.01 (-0.02 to 0.01) |
| Namibia | 0.00 (0.00 to 0.00) | 0.00 (0.00 to 0.00) | 0.00 (-0.02 to 0.02) |
| Nauru | 0.02 (0.02 to 0.03) | 0.02 (0.01 to 0.02) | -0.02 (-0.04 to 0.00) |
| Nepal | 0.01 (0.01 to 0.01) | 0.01 (0.01 to 0.01) | 0.00 (-0.02 to 0.01) |
| Netherlands | 0.06 (0.00 to 0.13) | 0.04 (0.00 to 0.08) | 0.05 (-0.03 to 0.14) |
| New Zealand | 0.00 (0.00 to 0.00) | 0.00 (0.00 to 0.00) | -0.01 (-0.02 to 0.00) |
| Nicaragua | 0.00 (0.00 to 0.00) | 0.00 (0.00 to 0.00) | -0.01 (-0.03 to 0.01) |
| Niger | 0.00 (0.00 to 0.00) | 0.00 (0.00 to 0.00) | -0.01 (-0.03 to 0.02) |
| Nigeria | 0.02 (0.02 to 0.02) | 0.02 (0.02 to 0.02) | 0.03 (0.02 to 0.03) |
| Niue | 0.00 (0.00 to 0.00) | 0.00 (0.00 to 0.00) | -0.02 (-0.03 to -0.01) |
| North Macedonia | 0.00 (0.00 to 0.00) | 0.00 (0.00 to 0.00) | 0.00 (-0.02 to 0.03) |
| Northern Mariana Islands | -0.02 (-0.03 to -0.01) | -0.03 (-0.03 to -0.02) | -0.03 (-0.06 to -0.01) |
| Norway | 0.00 (0.00 to 0.00) | 0.00 (0.00 to 0.00) | -0.05 (-0.06 to -0.04) |
| Oman | 0.06 (-0.04 to 0.17) | 0.05 (-0.04 to 0.14) | 0.06 (-0.04 to 0.16) |
| Pakistan | 0.00 (0.00 to 0.00) | 0.00 (0.00 to 0.00) | 0.00 (-0.01 to 0.01) |
| Palau | -0.03 (-0.03 to -0.02) | -0.03 (-0.03 to -0.02) | -0.04 (-0.07 to -0.01) |
| Palestine | -0.01 (-0.01 to -0.01) | -0.01 (-0.01 to -0.01) | -0.02 (-0.03 to -0.01) |
| Panama | 0.00 (0.00 to 0.00) | 0.00 (-0.01 to 0.00) | -0.01 (-0.02 to 0.00) |
| Papua New Guinea | 0.00 (0.00 to 0.00) | 0.00 (0.00 to 0.00) | -0.01 (-0.03 to 0.02) |
| Paraguay | 0.00 (0.00 to 0.00) | 0.00 (0.00 to 0.00) | -0.01 (-0.02 to 0.01) |
| Peru | 0.00 (0.00 to 0.00) | 0.00 (-0.01 to 0.00) | -0.01 (-0.02 to 0.00) |
| Philippines | 0.00 (0.00 to 0.00) | 0.00 (0.00 to 0.00) | -0.01 (-0.01 to 0.00) |
| Poland | -0.06 (-0.11 to -0.01) | -0.01 (-0.01 to 0.00) | -0.07 (-0.12 to -0.02) |
| Portugal | -0.04 (-0.09 to 0.00) | -0.03 (-0.07 to 0.00) | -0.05 (-0.09 to -0.01) |
| Puerto Rico | 0.00 (0.00 to 0.01) | 0.00 (0.00 to 0.01) | -0.01 (-0.03 to 0.01) |
| Qatar | 0.03 (0.03 to 0.03) | 0.03 (0.03 to 0.03) | 0.05 (0.01 to 0.09) |
| Republic of Korea | -2.56 (-5.50 to 0.48) | -1.85 (-3.99 to 0.34) | -2.53 (-5.48 to 0.51) |
| Republic of Moldova | -0.01 (-0.01 to -0.01) | -0.01 (-0.01 to -0.01) | -0.01 (-0.01 to 0.00) |
| Romania | 0.00 (0.00 to 0.00) | 0.00 (0.00 to 0.00) | -0.01 (-0.02 to 0.00) |
| Russian Federation | 2.62 (0.03 to 5.27) | 2.11 (0.03 to 4.24) | 2.61 (0.04 to 5.25) |
| Rwanda | -0.01 (-0.01 to -0.01) | -0.01 (-0.01 to -0.01) | 0.00 (-0.01 to 0.02) |
| Saint Kitts and Nevis | 0.00 (0.00 to 0.00) | 0.00 (0.00 to 0.00) | -0.01 (-0.03 to 0.01) |
| Saint Lucia | 0.00 (0.00 to 0.00) | 0.00 (0.00 to 0.00) | -0.01 (-0.02 to 0.00) |
| Saint Vincent and the Grenadines | 0.00 (0.00 to 0.00) | 0.00 (0.00 to 0.00) | -0.01 (-0.03 to 0.01) |
| Samoa | 0.00 (0.00 to 0.00) | 0.00 (0.00 to 0.00) | -0.02 (-0.05 to 0.01) |
| San Marino | 0.01 (0.01 to 0.01) | 0.01 (0.01 to 0.01) | -0.01 (-0.04 to 0.02) |
| Sao Tome and Principe | -0.01 (-0.01 to -0.01) | 0.00 (-0.01 to 0.00) | -0.01 (-0.02 to 0.01) |
| Saudi Arabia | -0.01 (-0.01 to -0.01) | -0.01 (-0.01 to -0.01) | -0.01 (-0.03 to 0.01) |
| Senegal | 0.00 (0.00 to 0.00) | 0.00 (0.00 to 0.00) | 0.01 (-0.02 to 0.04) |
| Serbia | 0.00 (0.00 to 0.00) | 0.00 (0.00 to 0.00) | -0.02 (-0.02 to -0.02) |
| Seychelles | -0.02 (-0.02 to -0.01) | -0.01 (-0.02 to -0.01) | -0.03 (-0.05 to -0.01) |
| Sierra Leone | -0.01 (-0.01 to -0.01) | -0.01 (-0.01 to -0.01) | 0.00 (-0.02 to 0.01) |
| Singapore | 0.06 (0.04 to 0.08) | 0.04 (0.03 to 0.06) | 0.07 (0.05 to 0.09) |
| Slovakia | -0.01 (-0.01 to -0.01) | 0.00 (0.00 to 0.00) | -0.01 (-0.02 to 0.00) |
| Slovenia | 0.00 (0.00 to 0.00) | 0.00 (0.00 to 0.00) | 0.01 (-0.02 to 0.04) |
| Solomon Islands | 0.01 (0.01 to 0.01) | 0.01 (0.01 to 0.01) | 0.00 (-0.02 to 0.02) |
| Somalia | 0.00 (0.00 to 0.00) | 0.00 (0.00 to 0.00) | 0.00 (-0.01 to 0.01) |
| South Africa | -0.01 (-0.01 to -0.01) | -0.01 (-0.01 to -0.01) | -0.02 (-0.03 to -0.02) |
| South Sudan | 0.02 (0.01 to 0.04) | 0.02 (0.00 to 0.03) | 0.02 (0.00 to 0.05) |
| Spain | -0.31 (-0.60 to -0.01) | -0.23 (-0.45 to 0.00) | -0.31 (-0.59 to -0.03) |
| Sri Lanka | 0.00 (0.00 to 0.00) | 0.00 (0.00 to 0.00) | 0.00 (-0.03 to 0.04) |
| Sudan | 0.00 (0.00 to 0.01) | 0.00 (0.00 to 0.01) | 0.00 (-0.02 to 0.02) |
| Suriname | 0.00 (0.00 to 0.01) | 0.00 (0.00 to 0.00) | -0.01 (-0.01 to 0.00) |
| Sweden | 0.01 (0.01 to 0.01) | 0.00 (0.00 to 0.00) | 0.00 (-0.02 to 0.01) |
| Switzerland | -0.20 (-0.39 to -0.02) | -0.18 (-0.35 to -0.01) | -0.17 (-0.38 to 0.04) |
| Syrian Arab Republic | 0.06 (0.03 to 0.08) | 0.05 (0.02 to 0.07) | -0.01 (-0.04 to 0.02) |
| Taiwan (Province of China) | -0.16 (-0.35 to 0.03) | -0.16 (-0.35 to 0.03) | -0.13 (-0.30 to 0.04) |
| Tajikistan | 0.00 (0.00 to 0.01) | 0.00 (0.00 to 0.00) | -0.02 (-0.04 to 0.00) |
| Thailand | 0.00 (0.00 to 0.00) | 0.00 (0.00 to 0.00) | -0.01 (-0.04 to 0.02) |
| Timor-Leste | 0.01 (0.01 to 0.01) | 0.01 (0.01 to 0.01) | -0.01 (-0.05 to 0.02) |
| Togo | 0.00 (0.00 to 0.00) | 0.00 (0.00 to 0.00) | 0.01 (-0.01 to 0.03) |
| Tokelau | -0.01 (-0.03 to 0.00) | -0.01 (-0.03 to 0.00) | -0.03 (-0.07 to 0.01) |
| Tonga | 0.01 (0.01 to 0.01) | 0.01 (0.01 to 0.01) | -0.01 (-0.03 to 0.00) |
| Trinidad and Tobago | 0.00 (0.00 to 0.00) | 0.00 (0.00 to 0.00) | -0.02 (-0.03 to -0.01) |
| Tunisia | 0.00 (0.00 to 0.00) | 0.00 (0.00 to 0.00) | -0.02 (-0.04 to 0.00) |
| Turkey | -0.28 (-0.54 to -0.01) | -0.14 (-0.27 to -0.01) | -0.28 (-0.54 to -0.02) |
| Turkmenistan | -0.01 (-0.01 to -0.01) | -0.01 (-0.01 to -0.01) | -0.02 (-0.03 to 0.00) |
| Tuvalu | -0.02 (-0.02 to -0.02) | -0.02 (-0.02 to -0.02) | -0.05 (-0.06 to -0.03) |
| Uganda | 0.00 (0.00 to 0.00) | 0.00 (0.00 to 0.00) | 0.01 (0.00 to 0.03) |
| Ukraine | 0.00 (0.00 to 0.00) | 0.00 (0.00 to 0.00) | -0.02 (-0.03 to -0.01) |
| United Arab Emirates | 0.06 (0.06 to 0.07) | 0.06 (0.06 to 0.06) | 0.05 (0.04 to 0.07) |
| United Kingdom | 0.00 (0.00 to 0.00) | 0.00 (0.00 to 0.00) | -0.02 (-0.03 to -0.01) |
| United Republic of Tanzania | 0.00 (0.00 to 0.00) | 0.00 (0.00 to 0.00) | 0.00 (-0.01 to 0.02) |
| United States of America | 1.32 (-0.22 to 2.89) | 1.07 (-0.18 to 2.33) | 1.26 (-0.26 to 2.80) |
| United States Virgin Islands | 0.00 (0.00 to 0.00) | 0.00 (0.00 to 0.00) | -0.01 (-0.01 to -0.01) |
| Uruguay | 0.00 (0.00 to 0.00) | 0.00 (0.00 to 0.00) | -0.03 (-0.03 to -0.02) |
| Uzbekistan | 0.00 (0.00 to 0.00) | 0.00 (0.00 to 0.00) | -0.01 (-0.03 to 0.01) |
| Vanuatu | 0.00 (0.00 to 0.00) | 0.00 (0.00 to 0.00) | -0.02 (-0.04 to 0.00) |
| Venezuela (Bolivarian Republic of) | 0.01 (0.01 to 0.02) | 0.02 (0.01 to 0.02) | 0.01 (0.00 to 0.02) |
| Viet Nam | -0.01 (-0.01 to -0.01) | -0.01 (-0.01 to -0.01) | -0.01 (-0.05 to 0.02) |
| Yemen | 0.01 (0.01 to 0.01) | 0.01 (0.01 to 0.01) | -0.01 (-0.02 to 0.00) |
| Zambia | 0.00 (0.00 to 0.00) | 0.00 (0.00 to 0.00) | 0.01 (0.00 to 0.02) |
| Zimbabwe | 0.00 (0.00 to 0.00) | 0.00 (0.00 to 0.00) | 0.01 (-0.01 to 0.03) |

Abbreviations: ASIR, age-standardised incidence rate; ASPR, age-standardised prevalence rate; ASYR, age-standardised YLD rate; CI, confidence interval; EAPC, estimated annual percentage change; YLD, year of life lived with disability.

**Supplementary Table 7** SDI quintile in 2019

| SDI quintile | Lower bound | Upper bound |
| --- | --- | --- |
| Low SDI | 0 | 0.454743 |
| Low-middle SDI | 0.454743 | 0.607679 |
| Middle SDI | 0.607679 | 0.689504 |
| High-middle SDI | 0.689504 | 0.805129 |
| High SDI | 0.805129 | 1 |

Abbreviations: SDI, socio-demographic index.
